# A randomised, placebo-controlled phase 3 study to evaluate the efficacy and safety of ASP0113, a DNA-based CMV vaccine, in seropositive allogeneic haematopoietic cell transplant recipients

**DOI:** 10.1016/j.eclinm.2021.100787

**Published:** 2021-03-19

**Authors:** Per Ljungman, Arancha Bermudez, Aaron C. Logan, Mohamed A. Kharfan-Dabaja, Patrice Chevallier, Rodrigo Martino, Gerald Wulf, Dominik Selleslag, Kazuhiko Kakihana, Amelia Langston, Dong-Gun Lee, Carlos Solano, Shinichiro Okamoto, Larry R. Smith, Michael Boeckh, John R. Wingard, Beth Cywin, Christine Fredericks, Christopher Lademacher, Xuegong Wang, James Young, Johan Maertens

**Affiliations:** a Department of Cellular Therapy and Allogeneic Stem Cell Transplantation, Karolinska University Hospital and Division of Hematology, Department of Medicine Huddinge, Karolinska Institutet, SE-14186, Stockholm, Sweden; b Department of Hematology, Hospital Universitario Marqués de Valdecilla, Instituto de Formación e Investigación Marqués de Valdecilla, 39008, Santander, Spain; c Division of Hematology and Blood and Marrow Transplantation, Department of Medicine, University of California, San Francisco, 94143, San Francisco, United States; d Department of Blood and Marrow Transplantation, H. Lee Moffitt Cancer Center and Research Institute and University of South Florida, 33612, Tampa, United States; e Service d'Hématologie Clinique, CHU de Nantes, 44093, Nantes, France and CRCINA / INSERM UMR1232 / CNRS ERL6001 IRS UN – 8 Quai Moncousu – BP 70721, 44007 Nantes cedex 1, France; f Division of Clinical Hematology, Hospital de la Santa Creu I Sant Pau, 08041, Barcelona, Spain; g Department of Hematology and Medical Oncology, University Medical Center Göttingen, D-37075, Göttingen, Germany; h Department of Hematology, AZ Sint-Jan Brugge-Oostende, 8000, Brugge, Belgium; i Hematology Division, Tokyo Metropolitan Komagome Hospital, 113-8677, Tokyo, Japan; j Winship Cancer Institute, Emory University, 30322, Atlanta, United States; k Division of Infectious Diseases, Department of Internal Medicine, Catholic Hematology Hospital and Seoul St. Mary's Hospital, College of Medicine, The Catholic University of Korea, 06591, Seoul, Republic of Korea; l Hematology Service, Hospital Clínico Universitario, Institute of Research INCLIVA and University of Valencia, 46010, Valencia, Spain; m Division of Hematology, Department of Medicine, Kelo University School of Medicine, 160-8582, Tokyo, Japan; n Vical Inc., 92121, San Diego, United States; o Vaccine and Infectious Disease Division, Fred Hutchinson Cancer Research Center and University of Washington, 98109, Seattle, United States; p Division of Hematology & Oncology, Department of Medicine, University of Florida, 32610, Gainesville, United States; q Astellas Pharma Global Development, Inc., 60062, Northbrook, United States; r Department of Microbiology and Immunology, K.U. Leuven and Department of Hematology, UZ Leuven, Leuven, Belgium

## Abstract

**Background:**

Cytomegalovirus (CMV) is a complication of allogeneic haematopoietic cell transplantation (allo-HCT). ASP0113, a DNA-based vaccine, contains two plasmids encoding human CMV glycoprotein B and phosphoprotein 65 (pp65). We assessed ASP0113 in CMV-seropositive allo-HCT recipients.

**Methods:**

In this phase 3, randomised, placebo-controlled study, CMV-seropositive allo-HCT recipients were randomly assigned (1:1) via interactive response technology to receive five injections of 1 mL of 5 mg/mL ASP0113 or placebo. The pharmacist and designated staff were unblinded. Masked syringes maintained the blind for patients and study personnel. Efficacy and safety analyses included patients who received ≥1 dose of ASP0113/placebo. The primary efficacy endpoint was the proportion of allo-HCT recipients with composite all-cause mortality and adjudicated CMV end-organ disease (EOD) by 1 year post-transplant. ClinicalTrials.gov: NCT01877655 (not recruiting).

**Findings:**

Patients were recruited between Sept 11, 2013 and Sept 21, 2016. Overall, 501 patients received ≥1 dose of ASP0113 (*n* = 246) or placebo (*n* = 255). The proportion of patients with composite all-cause mortality and adjudicated CMV EOD by 1 year post-transplant was 35.4% (*n* = 87) with ASP0113 and 30•2% (*n* = 77) with placebo (odds ratio 1.27; 95% confidence interval: 0.87 to 1.85; *p* = 0.205). Incidence of injection site-related treatment-emergent adverse events (TEAEs) was higher with ASP0113 than placebo. Overall incidence and severity of other TEAEs was similar between groups. T-cell response to pp65 increased over time and was greater with placebo than ASP0113 (*p* = 0.027).

**Interpretation:**

ASP0113 did not reduce overall mortality or CMV EOD by 1 year post-transplant. Safety findings were similar between groups.

**Funding:**

Astellas Pharma Global Development, Inc .

**Research in Context**

**Evidence before this study**

There is an unmet need to develop treatments with novel mechanisms of action that are associated with favourable safety profiles compared with antiviral therapies for the prevention of cytomegalovirus (CMV) end-organ disease in allogeneic haematopoietic cell transplant (allo-HCT) recipients. ASP0113 is a DNA-based vaccine containing two plasmids encoding glycoprotein B and phosphoprotein 65 from human CMV. In a phase 2 study of allo-HCT recipients, ASP0113 significantly reduced the occurrence and recurrence of CMV viraemia compared with placebo; it was well tolerated but was not significantly more immunogenic than placebo.

**Added value of this study**

The results of this phase 3 study show that ASP0113 did not reduce overall mortality and CMV end-organ disease through 1 year post-transplant. These findings reiterate the need to confirm promising phase 2 data with larger phase 3 studies, and highlight the difficulty of inducing immune responses against CMV in the setting of highly immunosuppressed allo-HCT recipients.

**Implications of all the available evidence**

ASP0113 was not effective for inducing immune responses to CMV when administered pre- and post-allo-HCT. Given that letermovir has demonstrated efficacy for the prophylaxis of CMV infection with limited toxicity, the development of vaccines with improved immunogenicity and novel vaccination strategies targeting the prevention of late or recurrent CMV infection/reactivation may be appropriate.

Alt-text: Unlabelled box

## 1 Introduction

Severely immunocompromised patients, including patients undergoing allogeneic haematopoietic cell transplantation (allo-HCT), are particularly vulnerable to cytomegalovirus (CMV) infection or reactivation [1]. CMV seropositivity (or prior exposure) is a known risk factor for CMV end-organ disease (EOD), bacterial and fungal infections, and increased mortality [2].

Pre-emptive therapies with ganciclovir or its valine ester valganciclovir are effective methods of preventing the development of CMV disease in allo-HCT recipients [2], although both are associated with toxicity. Treatment with ganciclovir or valganciclovir is associated with myelosuppression, as well as bacterial and fungal infections [3], [4], [5]. In view of these toxicities, the risk–benefit of administering potentially toxic agents as a prophylactic or pre-emptive treatment to patients who may be at low risk of developing CMV disease, should be carefully considered. Letermovir, a CMV DNA terminase complex inhibitor, has recently been approved by the US Food and Drug Administration and the European Medicines Agency for the prophylaxis of CMV infection and disease in adult CMV-seropositive allo-HCT recipients [6], [7], [8]. In a phase 3, double-blind, placebo-controlled study, letermovir significantly reduced the number of patients with clinically significant CMV infection by week 24 post-transplant (*p*<0.001), while maintaining a similar safety profile to placebo [6]. Furthermore, letermovir seemed to be associated with decreased all-cause mortality at week 24; however, late CMV reactivation occurred after discontinuation of letermovir, indicating a need for novel strategies to stimulate anti-CMV immunity. [6]

Despite recent advances and developments in the field of CMV infection and disease prophylaxis, an unmet need remains for therapies with novel mechanisms of action that are not associated with safety concerns. [9] A number of vaccines have been investigated for the prevention of CMV infection and/or disease in solid-organ transplant recipients.

A study of the immunogenicity of a CMV glycoprotein B (gB) vaccine combined with MF59 adjuvant, administered pre-transplant in kidney or liver transplant recipients, demonstrated significantly increased gB antibody titres compared with placebo in both CMV-seronegative (geometric mean titre: 12,537 [95% confidence interval (CI): 6593 to 23,840] versus 86 [95% CI: 63 to 118]; *p* < 0.0001) and CMV-seropositive (geometric mean titre: 118,395 [95% CI: 64,503 to 217,272] versus 24,682 [95% CI: 17,909 to 34,017]; *p* < 0.0001) recipients of the vaccine [10]. This study also demonstrated a significantly decreased duration of viraemia (*p* = 0.048) and reduced number of days of ganciclovir treatment (*p* = 0.0287) in CMV-seronegative recipients [10]. The modified vaccinia virus Ankara vector, currently being investigated, has been demonstrated to elicit potent humoral and cellular immune responses against multiple immunodominant CMV antigens in preclinical and *in vitro* studies [11]. These data support the development of a multi-antigenic vaccine candidate for controlling CMV in transplant recipients.

ASP0113, a DNA-based therapeutic vaccine, contains two plasmids, VCL-6365 and VCL-6368, encoding human CMV gB and phosphoprotein 65 (pp65), respectively, and is formulated with a CRL1005 poloxamer and benzalkonium chloride delivery system designed to enhance plasmid expression [12]. In a phase 1 study of healthy individuals, ASP0113 was well tolerated and immunogenic [13]. In a phase 2 study of allo-HCT recipients, ASP0113 was well tolerated and significantly reduced the occurrence and recurrence of CMV viraemia compared with placebo, but was not statistically significantly more immunogenic than placebo [14]. In another phase 2 study, despite not demonstrating superior efficacy or immunogenicity in the prevention of CMV viraemia in CMV-seronegative transplant recipients receiving a kidney from a CMV-seropositive donor, ASP0113 did demonstrate a safety profile that was generally similar to placebo [15].

Our study aimed to evaluate the efficacy, safety, and immunogenicity of ASP0113 in CMV-seropositive allo-HCT recipients.

## 2 Methods

### 2.1 Study design and participants

This was a randomised, double-blind, placebo-controlled, multicentre phase 3 study conducted at 95 sites in 11 countries (Australia [4], Belgium [4], Canada [4], France [5], Germany [13], Japan [10], South Korea [4], Spain [11], Sweden [5], Taiwan [3] and the USA [32]; appendix 1). Eligible patients were CMV-seropositive allo-HCT recipients aged ≥18 years who were planning to undergo one of the following: a sibling donor transplant with a 7/8 human leucocyte antigen (HLA)-A, -B, -C, -DRβ1 match utilising high-resolution typing, or an 8/8 HLA-A, -B, -C, -DRβ1 match utilising low- or high-resolution typing, or an unrelated donor transplant with a 7/8 or 8/8 HLA-A, -B, -C, -DRβ1 match utilising high-resolution typing, as a minimum requirement. Patients were excluded if they had active CMV infection or disease, had received treatment for active CMV infection or disease within 3 months before transplant, had planned CMV prophylactic antiviral therapy or CMV-specific immunoglobulins from randomisation through primary study period completion (day 365), or had a modified HCT-specific comorbidity index score ≥4 [16]. Patients were also excluded if they were known to be positive for human immunodeficiency virus, hepatitis B virus, or hepatitis C virus; had unknown CMV serostatus; had received alemtuzumab within 60 days before transplant; had T-cell depletion of a donor cell product; had received a CMV vaccine (including any prior exposure to ASP0113); had received an allo-HCT within 1 year before transplant; had residual chronic graft-versus-host disease (GVHD) from a previous HCT; or had a platelet count of <50,000/mm^3^ within 3 days before randomisation. Use of anti-thymocyte globulin (ATG) during transplant conditioning was permitted as per institutional preference.

An Independent Ethics Committee (IEC) or Institutional Review Board (IRB) reviewed the ethical appropriateness of the study before it was conducted, and their approval of the study protocol was obtained before authorisation of a drug shipment to a study site. An IEC or IRB provided approval of all written informed consent as per national regulations. The study was conducted in accordance with the study protocol, Good Clinical Practice requirements, International Council for Harmonisation of Technical Requirements for Human Use guidelines, and other applicable regulations and guidelines for conducting clinical studies and ethical principles that have their origin in the Declaration of Helsinki.

### 2.2 Randomisation and masking

Patients were randomly assigned in a 1:1 ratio to receive ASP0113 or placebo. Randomisation was performed via interactive response technology after the completion of the screening period and at least 3–14 days before the anticipated transplant day/donor cell infusion (Day 0). Randomisation was stratified by donor–recipient relatedness and by donor CMV serostatus. Patients were assigned to a double-blinded treatment group in the order in which they were entered into the interactive response technology system. Only the pharmacist and designated staff, including the person(s) administering the vaccine, were unblinded to treatment. The syringes were masked before dosing to maintain the blind for patients and other study personnel. Randomisation was blinded, so allocation to treatment group could not influence the likelihood of receiving treatment.

### 2.3 Procedures

Patients were randomly assigned to receive 1 mL of 5 mg/mL ASP0113 (manufactured by Vical, San Diego, CA, USA) or placebo via intramuscular injection (appendix 2). Patients received five intramuscular doses of either ASP0113 or placebo on Days −14 to −3, 14 to 40, 60 ± 5, 90 ± 10, and 180 ± 10. CMV viral load plasma samples were collected weekly (±2 days) from Day 0 to Day 100, every other week (±5 days) from Day 101 to Day 180, and every 30 days (±5 days) from Day 181 to Day 365. All CMV viral load plasma testing was performed by the central laboratory (Abbott Molecular Inc., Des Plaines, IL, USA; although they could be tested at a local laboratory if approved by the sponsor and at the discretion of the investigator). Pre-emptive therapy could then be started based on the central or local laboratory assessment and the patient's clinical condition.

Allo-HCT recipients were followed for 1 year post-transplant for overall mortality and adjudicated CMV EOD, CMV viraemia, and adjudicated CMV-specific antiviral therapy use. Allo-HCT recipients were evaluated for local and systemic reactogenicity 15 min and 60 min (±10 min) after each study drug injection and for 7 days after each study drug injection by patient reporting in a diary.

Treatment-emergent adverse events (TEAEs) and serious adverse events (SAEs) were collected for 30 days after the last study drug injection. All events requiring adjudication, grade ≥3 TEAEs, and SAEs were collected from 31 days after the last dose of study drug through to 1 year post-transplant.

Immunogenicity was measured by T-cell responses to pp65- and gB-specific antibody levels through 1 year after the first study drug injection. T-cell responses to pp65 were measured in peripheral blood mononuclear cells using an *ex vivo* pp65-specific IFN-γ enzyme-linked ImmunoSpot® assay. Antibody responses to gB were measured in serum samples using a gB-specific serum immunoglobulin G-binding enzyme-linked immunosorbent assay-based platform. For both T-cell responses to pp65 and antibody responses to gB, six measurements were taken during the study on Day −14 to −3, Day 14 to 40, Day 70 ± 5 to 74 ± 5, Day 90 ± 10, Day 180 ± 10 and Day 365 + 14. Samples for T-cell assays were not collected if the absolute lymphocyte count was confirmed to be <500 mm^3^ by a local or central laboratory.

### 2.4 Outcomes

The primary efficacy endpoint was the proportion of allo-HCT recipients with composite all-cause mortality and adjudicated CMV EOD by 1 year post-transplant. Secondary efficacy endpoints were: time to first protocol-defined CMV viraemia (CMV plasma viral load ≥1000 IU/mL) through 1 year post-transplant; time to first use of adjudicated CMV-specific antiviral therapy after the first study drug injection through 1 year post-transplant; the proportion of patients with a composite endpoint of protocol-defined CMV viraemia and CMV-specific antiviral therapy use per the adjudication committee; time to first occurrence of either use of adjudicated CMV-specific antiviral therapy or adjudicated CMV EOD rate; and all-cause mortality at 1 year post-transplant. Exploratory endpoints included, but were not limited to, the proportion of all-cause mortality and adjudicated CMV EOD at 6 months, the proportion of adjudicated CMV EOD, the incidence of grade 3–4 acute GVHD (aGVHD), the proportion of graft rejection, and the incidence of engraftment. A subgroup analysis of the primary endpoint stratified by whether ATG was included in the patients’ conditioning regimen and whether the patients received a myeloablative conditioning regimen was conducted.

Safety endpoints included adverse events classified by the Medical Dictionary for Regulatory Activities (MedDRA) version 16.0 [17] and reported using the National Cancer Institute's Common Terminology Criteria for Adverse Events version 4.03 grading scale, [18] vital signs, physical examinations, local reactogenicity signs, and clinical laboratory assessments. A data monitoring committee monitored the safety of patients.

The immunogenicity endpoints through 1 year after the first study drug injection were T-cell responses to pp65 and gB-specific antibody levels.

### 2.5 Statistical analyses

The sample size was estimated based on a phase 2 study of the efficacy of ASP0113 in HCT recipients; [14] to detect the estimated difference in mortality and CMV EOD from the phase 2 study (35.3% vs 22.5%), the study needed a sample size of at least 424 (212 per arm) to have 80% power at the two-sided significance level of 0.050. A sample size of 500 (250 per arm) was expected to have 86% power for the composite endpoint. A comparison between ASP0113 and placebo with respect to the primary endpoint included data from the time of the first dose of the study drug through 1 year post-transplant in the follow-up period.

The primary analysis was performed using the Cochran–Mantel–Haenszel test, which was stratified by factors for randomisation (donor–recipient relatedness and donor CMV serostatus) and a supportive analysis of time to first occurrence of death or CMV EOD using a Cox proportional hazards model, [19] which included treatment and stratification factors for randomisation.

The key secondary endpoints of time to first protocol-defined CMV viraemia through 1 year post-transplant and time to first use of adjudicated CMV-specific antiviral therapy use through 1 year post-transplant were both compared between treatments using a Cox proportional subdistributional hazards model [19] with factors for treatment, donor–recipient relatedness, and donor CMV serostatus. This model accounts for the competing risk of death, after which these key secondary endpoints can no longer be observed.

Efficacy analyses were conducted on the full analysis set (FAS), which consisted of all randomised patients who received at least one dose of the study drug. The safety analysis set (SAF) consisted of all randomised patients who received at least one dose of the study drug. The immunogenicity analysis set consisted of all patients who received at least one dose of the study drug and for whom at least one post-transplant immunogenicity measurement was available. All statistical analyses were conducted using SAS® version 9.4 or higher on Unix.

This study is registered on ClinicalTrials.gov (NCT01877655).

### 2.6 Role of the funding source

The study sponsor, Astellas Pharma Global Development, Inc., was involved in the design or conception of the study, the analysis and interpretation of the data and drafting and critically reviewing the publication. The authors who are employees of the study sponsor were Beth Cywin, Christine Fredericks, Christopher Lademacher, Xuegong Wang and James Young. The corresponding author had full access to all the data in the study and had final responsibility for the decision to submit this final version for publication.

## 3 Results

**Fig. 1:**
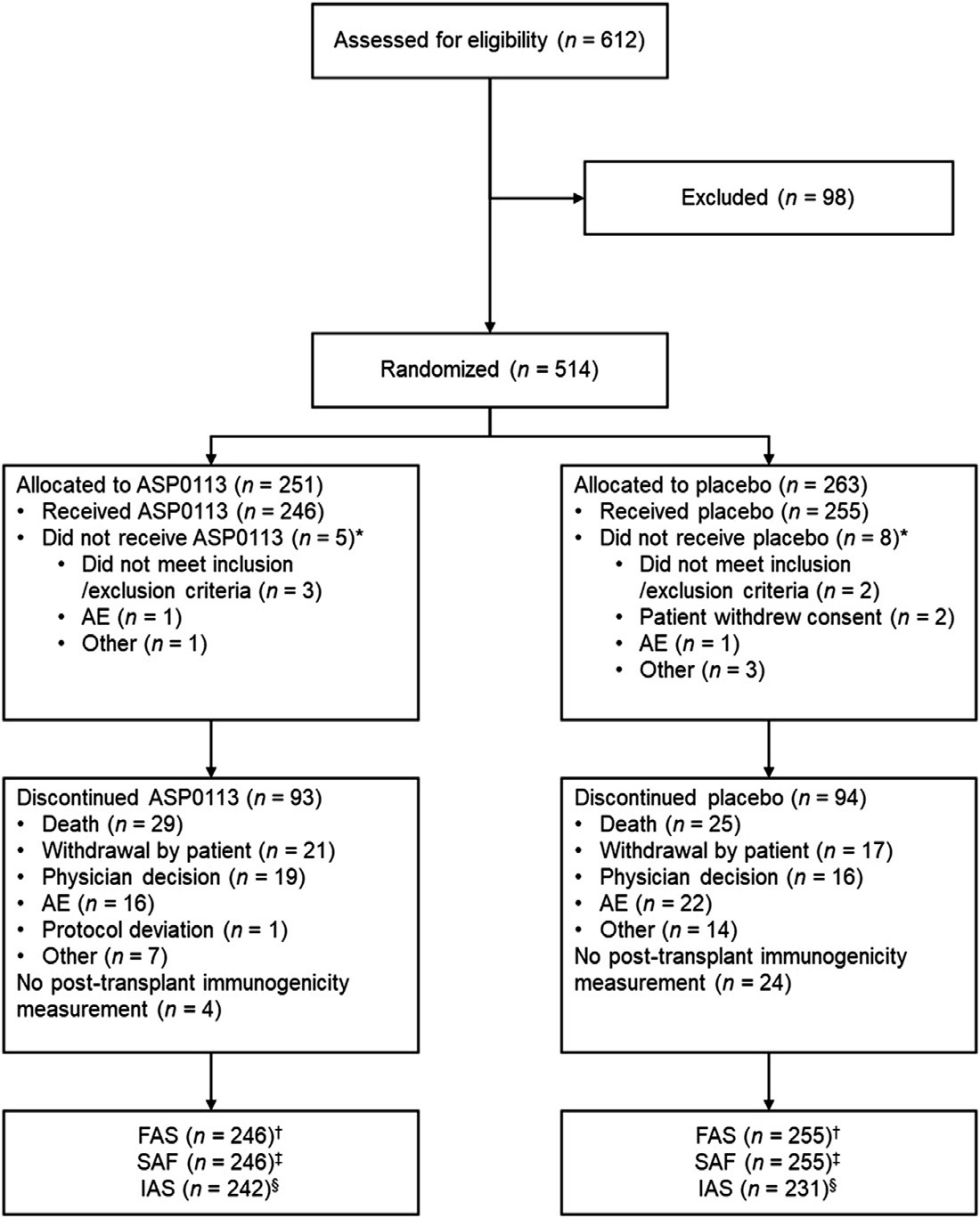
Trial profile *Randomisation was blinded, so allocation to treatment group could not influence the likelihood of receiving treatment. †The FAS consisted of all randomised patients who received at least one dose of randomly assigned study drug. ‡The SAF consisted of all randomised patients who received at least one dose of study drug. §The IAS included all patients who received at least one dose of study drug and for whom at least one post-transplant immunogenicity measurement was available. AE=adverse event; FAS=full analysis set; IAS=immunogenicity analysis set; SAF=safety analysis set.

Patients were recruited between Sept 11, 2013 and Sept 21, 2016. Overall, 612 patients were assessed for eligibility. Of 514 randomly assigned patients, 501 received at least one dose of ASP0113 (*n* = 246) or placebo (*n* = 255) and were included in the FAS and the SAF (Fig. 1). The number of patients who discontinued treatment for any reason was 93 in the ASP0113 group (18.6%) and 94 in the placebo group (18.8%). Most patients were white (71.3%) and male (57.9%), with a median age (interquartile range [IQR]) of 54 years (44–62). For donors, more were white (42.9%) than any other ethnicity; most were male (61.5%), and the median age (IQR) was 38 years (27–51; Table 1). More donors were CMV seropositive than CMV seronegative in both the ASP0113 (58.9%) and placebo (61.2%) groups. The number of patients who received each of the five study drug injections is provided in Table 2.

**Table 1:** Demographics and baseline characteristics (full analysis set).

	ASP0113 (*n* = 246)	Placebo (*n* = 255)	Total (*N* = 501)
**Recipient characteristics**			
Male sex	137 (55.7)	153 (60.0)	290 (57.9)
Median age (IQR), years	54 (43–61)	54 (44–62)	54 (44–62)
Race			
White	174 (70.7)	183 (71.8)	357 (71.3)
Asian	50 (20.3)	45 (17.6)	95 (19.0)
Black or African American	7 (2.8)	6 (2.4)	13 (2.6)
Other	15 (6.1)	21 (8.2)	36 (7.2)
Strata			
Related seropositive donor	72 (29.3)	63 (24.7)	135 (26.9)
Related seronegative donor	26 (10.6)	28 (11.0)	54 (10.8)
Unrelated seropositive donor	73 (29.7)	93 (36.5)	166 (33.1)
Unrelated seronegative donor	75 (30.5)	71 (27.8)	146 (29.1)
Most frequent indications for transplant			
Acute myelogenous leukaemia	96 (39.0)	123 (48.2)	219 (43.7)
Myelodysplastic syndrome	49 (19.9)	45 (17.6)	94 (18.8)
Acute lymphocytic leukaemia	34 (13.8)	31 (12.2)	65 (13.0)
Lymphoma	31 (12.6)	32 (12.5)	63 (12.6)
Conditioning regimen			
Myeloablative	120 (48.8)	122 (47.8)	242 (48.3)
Non-myeloablative	124 (50.4)	131 (51.4)	255 (50.9)
Missing	2 (0.8)	2 (0.8)	4 (0.8)
ATG			
Yes	43 (17.5)	45 (17.6)	88 (17.6)
No	203 (82.5)	210 (82.4)	413 (82.4)
**Donor characteristics**			
Male	147 (59.8)	161 (63.1)	308 (61.5)
Median age (IQR), years	39 (27–51)	37 (27–50)	38 (27–51)
Race			
White	103 (41.9)	112 (43.9)	215 (42.9)
Asian	49 (19.9)	41 (16.1)	90 (18.0)
Black or African American	5 (2.0)	5 (2.0)	10 (2.0)
American Indian or Alaska native	1 (0.4)	1 (0.4)	2 (0.4)
Other	71 (28.9)	86 (33.7)	157 (31.3)
Unknown	17 (6.9)	10 (3.9)	27 (5.4)

Data are *n* (%) unless otherwise specified. ATG=anti-thymocyte globulin; IQR, interquartile range.

**Table 2:** Number of patients who received each study drug injection (full analysis set).

Study drug injection, n (%)	ASP0113 (*n* = 246)	Placebo (*n* = 255)	Total (*n* = 501)
First injection	246 (100)	254 (99.6)	500 (99.8)
Second injection	207 (84.1)	213 (83.5)	420 (83.8)
Third injection	182 (74.0)	192 (75.3)	374 (74.7)
Fourth injection	173 (70.3)	189 (74.1)	362 (72.3)
Fifth injection	148 (60.2)	155 (60.8)	303 (60.5)

Patients received intramuscular doses of either ASP0113 or placebo on Days −14 to −3, 14 to 40, 60±5, 90±10, and 180±10.

**Fig. 2:**
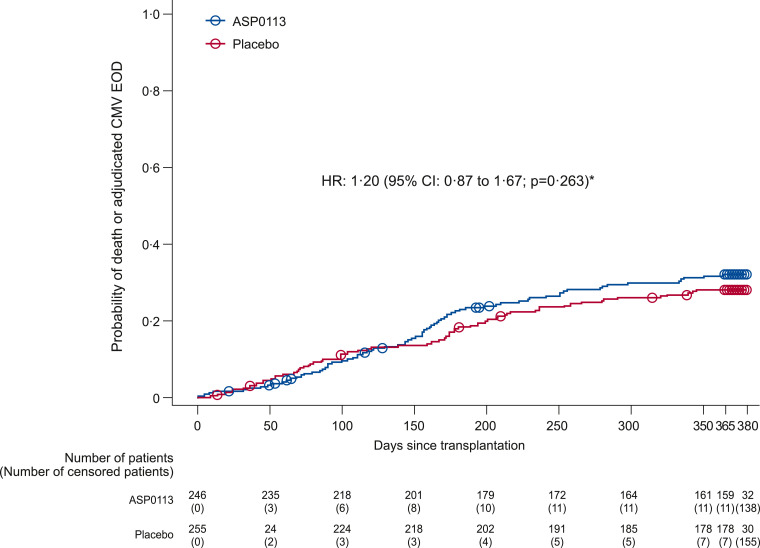
Time to composite of all-cause mortality and adjudicated CMV EOD (full analysis set) *Parameter estimate from a Cox proportional hazards model with treatment and randomisation strata. *p*-value based on Cox proportional hazards model parameter estimate for the treatment effect. Circles indicate censored observations. CMV=cytomegalovirus; EOD=end-organ disease; HR=hazard ratio.

The proportion of allo-HCT recipients with composite of all-cause mortality and adjudicated CMV EOD through 1 year post-transplant was 35.4% (*n* = 87) in the ASP0113 group and 30.2% (*n* = 77) in the placebo group (odds ratio [OR] 1.27; 95% CI: 0.87 to 1.85; *p* = 0.205; Table 3). There was also no statistically significant difference for the time to occurrence of the primary endpoint between the ASP0113 and placebo groups (hazard ratio 1.20; 95% CI: 0.87 to 1.67; *p* = 0.263; Fig. 2).

**Table 3:** Primary efficacy composite endpoint of all-cause mortality and adjudicated cytomegalovirus end-organ disease (full analysis set).

Parameter, n (%)	ASP0113 (*n* = 246)	Placebo (*n* = 255)	Odds ratio⁎ (95% CI) p-value
Total composite	87 (35.4)	77 (30.2)	1.27 (0.87 to 1.85) *p* = 0.205
All-cause mortality	78 (31.7)	72 (28.2)	
Known deaths†	65 (26.4)	65 (25.5)	
Unknown survival status due to withdrawal of consent	13 (5.3)	7 (2.7)	
Adjudicated CMV EOD	15 (6.1)	9 (3.5)	

⁎ ASP0113 vs placebo.

† Deaths listed here are from transplant through Day 365. Analysis was performed using the Cochran–Mantel–Haenszel test stratified by factors for randomisation (donor–recipient relatedness and donor CMV serostatus). CI=confidence interval; CMV=cytomegalovirus; EOD=end-organ disease.

**Fig. 3:**
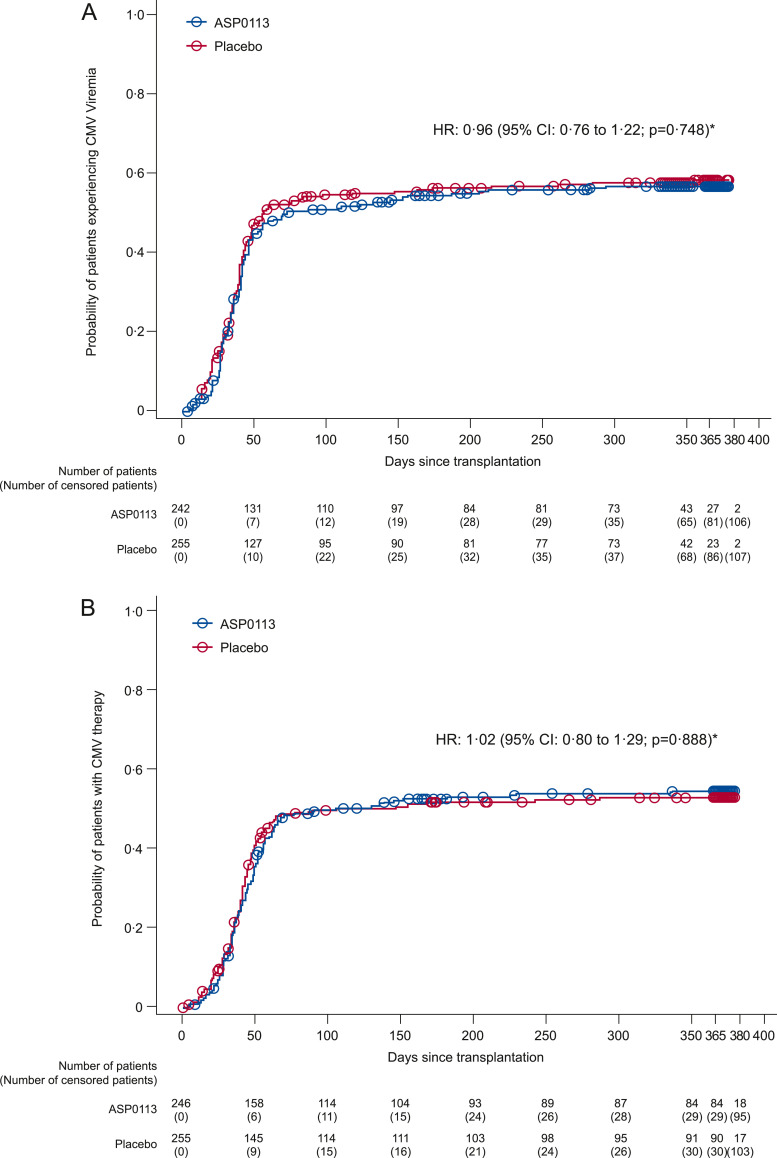
(A) Time to first protocol-defined CMV viraemia through 1 year post-transplant. (B) Time to first use of adjudicated CMV-specific antiviral therapy after first study vaccine injection through 1 year post-transplant *Parameter estimate from a Cox proportional hazards model with treatment and randomisation strata, adjusting for death as a competing risk. *p*-value based on Cox proportional hazards model parameter estimate for the treatment effect. Circles indicate censored observations. CMV=cytomegalovirus; HR=hazard ratio.

No statistically significant differences were found between ASP0113 and placebo for any of the secondary endpoints (Table 4) or exploratory efficacy endpoints (Table 5). There were no statistically significant differences for the time to first protocol-defined CMV viraemia (1-hazard ratio [1-HR] 0.04; 95% CI: −0.22 to 0.24; *p* = 0.748) or for the time to first use of adjudicated CMV-specific antiviral therapy (1-HR −0.02; 95% CI: −0.29 to 0.20; *p* = 0.888) between the ASP0113 and placebo groups (Fig. 3). In subgroup analyses of the primary endpoint, there were no statistically significant differences between the ASP0113 and placebo groups when patients were stratified by whether ATG was included in their conditioning regimen (*p* = 0.382) or whether they received a myeloablative conditioning regimen (*p* = 0.563; Table 6).

**Table 4:** Secondary endpoints (full analysis set).

	ASP0113 (*n* = 246)	Placebo (*n* = 255)	HR/1-HR/OR (95% CI) *p*-value
CMV viraemia rate, % (95% CI)	56.7 (50.1 to 62.8)	58.6 (52.0 to 64.6)	HR 0.96 (0.76 to 1.22) *p* = 0.748
Adjudicated CMV-specific antiviral therapy rate, % (95% CI)	54.6 (48.1 to 60.6)	53.2 (46.8 to 59.1)	HR 1.02 (0.80 to 1.29) *p* = 0.888
A composite of protocol-defined CMV viraemia and adjudicated CMV-specific antiviral therapy use, n (%)	150 (61.0)	155 (60.8)	OR 1.05 (0.73 to 1.51) *p* = 0.802
First occurrence of adjudicated CMV-specific antiviral therapy or adjudicated CMV EOD rate, % (95% CI)	55.4 (48.9 to 61.5)	54.4 (48.0 to 60.3)	1-HR –0.01 (–0.28 to 0.20) *p* = 0.928
Mortality, n (%)	78 (31.7)	72 (28.2)	OR 1.18 (0.81 to 1.73) *p* = 0.393

1-HR=1-hazard ratio; CI=confidence interval; CMV=cytomegalovirus; EOD=end-organ disease; HR=hazard ratio; OR=odds ratio.

**Table 5:** Exploratory efficacy endpoints (full analysis set).

n (%)	ASP0113 (*n* = 246)	Placebo (*n* = 255)	Odds ratio (95% CI) *p*-value
Composite endpoint of all-cause mortality and adjudicated CMV EOD at 6 months post-transplant, n (%)	54 (22.0)	45 (17.6)	1.36 (0.87 to 2.13) *p* = 0.184
Incidence of adjudicated CMV EOD, n (%)	15 (6.1)	9 (3.5)	1.81 (0.78 to 4.19) *p* = 0.162
Incidence of grade 3–4 aGVHD, n (%)	26/239 (10.9)	30/252 (11.9)	0.89 (0.51 to 1.55) *p* = NR
Incidence of graft rejection/poor graft function, n (%)	5/239 (2.1)	7/252 (2.8)	0.75 (0.23 to 2.42) *p* = NR
Incidence of engraftment, n (%)	238/239 (99.6)	248/252 (98.4)	3.82 (0.41 to 35.39) *p* = NR

aGVHD=acute graft-versus-host disease; CI=confidence interval; CMV=cytomegalovirus; EOD=end-organ disease; NR=not reported.

**Table 6:** Subgroup analyses of the primary endpoint (full analysis set).

n/N (%)	ASP0113	Placebo	Odds ratio (95% CI)	*p*-value
ATG included in conditioning regimen	16/43 (37.2)	11/45 (24.4)	1.83 (0.73 to 4.59)	*p* = 0.382
ATG not included in conditioning regimen	71/203 (35.0)	66/210 (31.4)	1.17 (0.78 to 1.77)	
Myeloablative conditioning regimen	35/120 (29.2)	34/122 (27.9)	1.07 (0.61 to 1.86)	*p* = 0.563
Non-myeloablative conditioning regimen	50/124 (40.3)	43/131 (32.8)	1.38 (0.83 to 2.31)	

ATG=anti-thymocyte globulin; CI=confidence interval.

All patients in the SAF experienced at least one TEAE (Table 7 and supplementary table S1). Minimal differences were observed between the safety profiles of the ASP0113 and placebo groups, with the exception of a higher proportion of patients in the ASP0113 group experiencing drug-related TEAEs versus the placebo group (78.9% [*n* = 194] vs 29.0% [*n* = 74]). The difference observed was mostly due to a higher number of mild to moderate injection-site-related TEAEs (eg, injection-site pain, injection-site erythema, injection-site induration, and injection-site swelling) in the ASP0113 group compared with the placebo group (Table 8).

**Table 7:** Summary of treatment-emergent adverse events and deaths (safety analysis set).

Parameter, n (%)	ASP0113 (*n* = 246)	Placebo (*n* = 255)
TEAEs	246 (100)	255 (100)
Drug-related⁎ TEAEs	194 (78.9)	74 (29.0)
SAEs	221 (89.8)	221 (86.7)
Drug-related⁎ serious TEAEs	22 (8.9)	13 (5.1)
TEAEs leading to death	61 (24.8)	63 (24.7)
Drug-related⁎ TEAEs leading to death	4 (1.6)	1 (0.4)
TEAEs leading to permanent discontinuation of the study drug	48 (19.5)	45 (17.6)
Drug-related TEAEs leading to permanent discontinuation of the study drug	5 (2.0)	4 (1.6)
Deaths†	67 (27.2)	68 (26.7)

⁎ Possible or probable, as assessed by the investigator, or records where relationship is missing.

† Deaths listed here are those from transplant through to the day after Day 380. AE=adverse event; SAE=serious adverse event; TEAE=treatment-emergent adverse event.

**Table 8:** Drug-related treatment-emergent adverse events experienced by >5% of patients (safety analysis set).

Drug-related TEAE occurring in >5% of patients, n (%)	ASP0113 (*n* = 246)				Placebo (*n* = 255)			
	Total	Mild	Moderate	Severe	Total	Mild	Moderate	Severe
Injection-site pain	183 (74.4)	120 (48.8)	62 (25.2)	1 (0.4)	39 (15.3)	38 (14.9)	1 (0.4)	0
Injection-site erythema	45 (18.3)	39 (15.9)	6 (2.4)	0	4 (1.6)	4 (1.6)	0	0
Injection-site induration	36 (14.6)	32 (13.0)	4 (1.6)	0	2 (0.8)	2 (0.8)	0	0
Injection-site swelling	24 (9.8)	20 (8.1)	4 (1.6)	0	4 (1.6)	4 (1.6)	0	0

TEAE severity grading was according to MedDRA version 16.0, mild: grade 0–1; moderate: grade 2; and severe: grade 3. MedDRA=Medical Dictionary for Regulatory Activities; TEAE=treatment-emergent adverse event.

SAEs were reported in 221 (89.8%) patients in the ASP0113 group and 221 (86.7%) patients in the placebo group. TEAEs leading to death that were considered by investigators to be study drug-related occurred in four (1.6%) patients in the ASP0113 group (haemolytic anaemia, organising pneumonia, recurrent acute myeloid leukaemia, and aGVHD in the intestine and liver) and one (0.4%) patient in the placebo group (acute respiratory distress syndrome). TEAEs resulting in the permanent discontinuation of the study drug were reported in 48 (19.5%) and 45 (17.6%) patients in the ASP0113 and placebo groups, respectively. Overall, 135 (26.9%) patients died during the study (67 [27.2%] in the ASP0113 group and 68 [26.7%] in the placebo group).

**Fig. 4:**
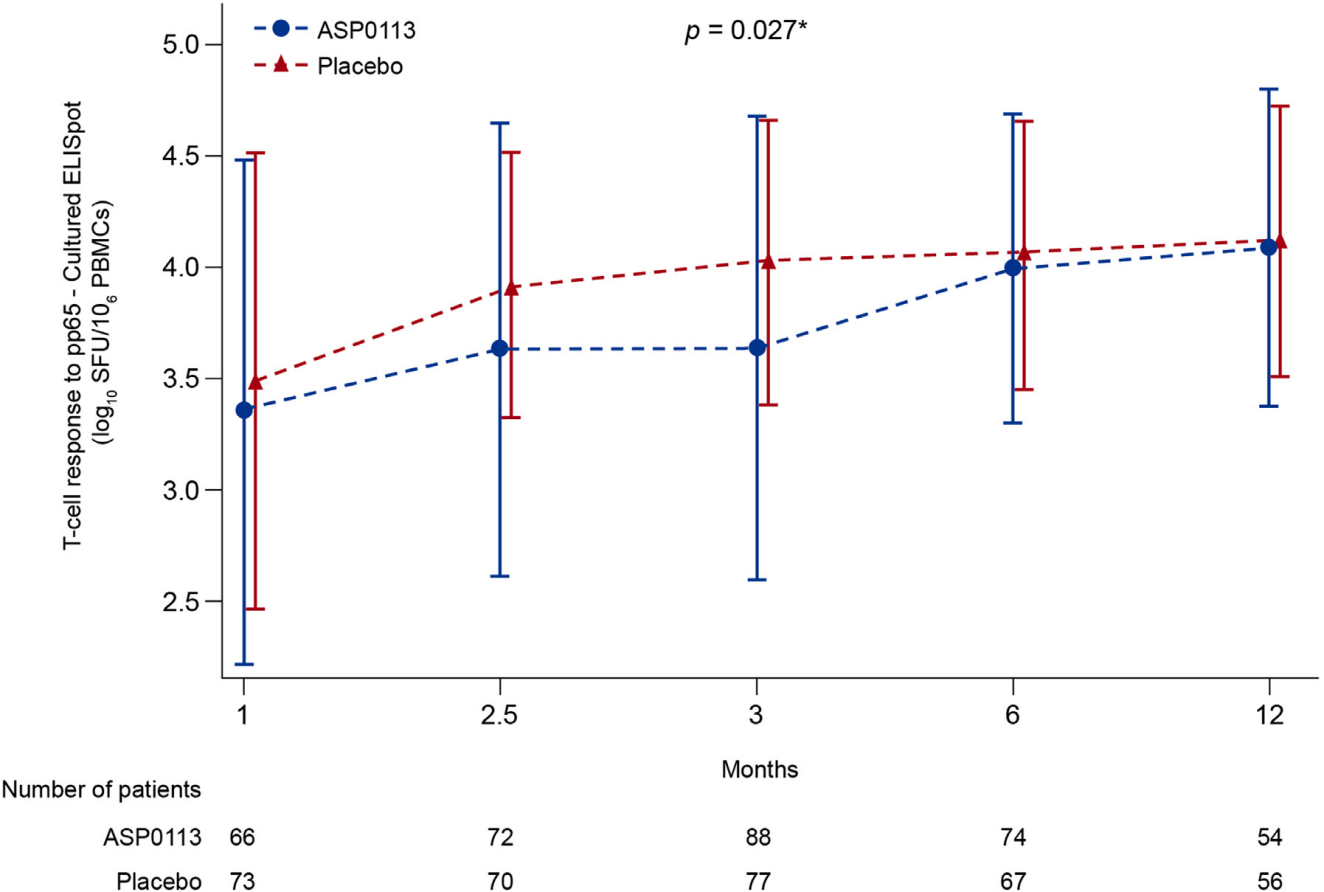
T-cell response to pp65 (immunogenicity analysis set) Data are mean and standard deviation. Interaction and overall *p*-value are based on the repeated measure model with post-baseline T-cell response to pp65 values on the 10 based log scale as the outcome variable, randomisation stratum, treatment, time, and treatment by time interaction as the explanatory variables. The self-consistent sandwich correlation structure was used. As zero values are possible these were replaced by 1 before taking the log transform of the pp65 values. *p-value is based on Wilcoxon rank sum test at each visit. Circles indicate censored observations. Time intervals on the x-axis are not equal. ELISpot=enzyme-linked immune absorbent spot; PBMC=peripheral blood mononuclear cell; pp65=phosphoprotein 65; SFU=spot-forming units.

**Fig. 5:**
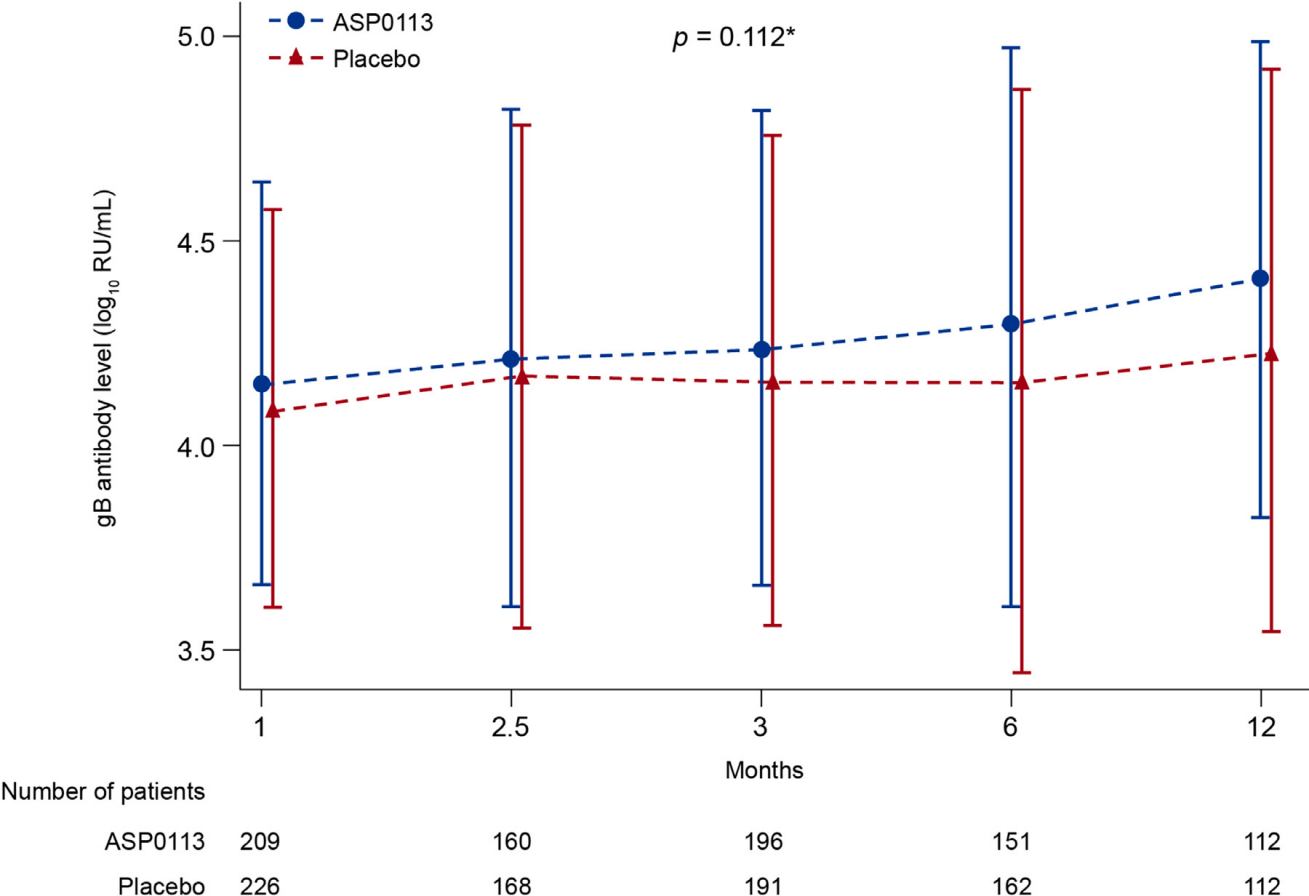
gB antibody levels (immunogenicity analysis set) Data are mean and standard deviation. Interaction and overall *p*-value are based on the repeated measure model with post-baseline gB antibody level values on the 10 based log scale as the outcome variable, randomisation stratum, treatment, time, and treatment by time interaction as the explanatory variables. The self-consistent sandwich correlation structure was used. Time intervals on the x-axis are not equal. *p-value is based on Wilcoxon rank sum test at each visit. Circles indicate censored observations. gB=glycoprotein B; RU=relative units.

The mean T-cell response to pp65 increased over time; the increase was greater with placebo than with ASP0113 (*p* = 0.027; Fig. 4). The mean gB-specific antibody response increased over time during the study (Fig. 5). Although there was no statistically significant difference in mean gB-specific antibody response between ASP0113 and placebo overall (*p* = 0.112), the mean gB-specific antibody response was significantly greater in the ASP0113 group compared with the placebo group at Month 12 (*p* = 0.036).

## 4 Discussion

This phase 3 study investigating ASP0113 in allo-HCT recipients did not achieve its primary endpoint, which was largely unexpected given the results from the phase 2 study but illustrates the need for phase 3 studies to validate new treatments in large cohorts of patients. [14] Several possible reasons can be given to explain the results of this study. The primary endpoint was chosen for this study as it would capture the ability of ASP0113 to reduce both direct (CMV disease) and indirect effects of CMV as well as possible adverse effects associated with the use of pre-emptive therapy, both of which may result in a reduction of mortality. The primary endpoint may have been unsuitable for determining the efficacy of ASP0113 in this patient population, however. Instead, one might speculate that an endpoint such as clinically significant CMV infections may have been more appropriate; this has recently been used in two studies of prophylactic antiviral therapies. [6,20] In the analysis of the secondary endpoints in this study, however, this alternative endpoint would not have altered the outcome of the study. Interestingly, ASP0113 did not induce a potent CMV-specific immune response, and the immune response to pp65 in the ASP0113 group was inferior to that with placebo, which contrasts with the results of the phase 2 study and other studies investigating vaccines for the prevention of CMV infection. [14,21] The explanation for this finding is unknown as both our study and the phase 2 study used ASP0113 that was produced via the same manufacturing process and met the same release criteria. In addition, all laboratories included in our study adhered to standard quality control measures, so it is unlikely that there were differences in testing methodologies between the central and local laboratories. The patient populations in this phase 3 study and the phase 2 study met similar inclusion and exclusion criteria; the main difference between the studies was that this phase 3 study enrolled patients from numerous countries, whereas the phase 2 study enrolled patients from 16 US centres. [14] In addition, the pre-transplant dose of ASP0113 was administered earlier in the phase 2 study (Days –21 to –2) compared with this phase 3 study (Days –14 to –3), and this phase 3 study contained an additional dose of ASP0113 at Day 60±5.

In theory, ASP0113 could have limited the risk of late CMV infection, but these data were not collected, which is a limitation of the study. However, considering the absence of a potent CMV-specific immune response, this possibility is unlikely. The development of an effective CMV vaccine was viewed as a promising therapeutic option to prevent CMV EOD, but the failure of this study has underlined the difficulty of producing an effective vaccine for this purpose. Other vaccines against CMV are in development using different mechanisms of action and it remains to be seen if these are more effective for the prevention of CMV EOD.

Although this was a global, multicentre study of CMV-seropositive allo-HCT recipients, we recognise that the proportion of African and/or Afro-American allo-HCT recipients was low. However, we do not believe that the results of our study would have been substantially different if there was a larger African and/or Afro-American population. In addition, no site contributed more than 5.4% of the total number of patients in our study. Overall, 46% of patients were randomised at EU sites, 33% at US sites, 18% at Asian sites and 3% at Canadian sites. These data suggest that clustering in each of the participating countries was not a limitation of our study.

Overall, safety findings were similar between the ASP0113 and placebo groups, with the exception of a higher percentage of drug-related TEAEs in the ASP0113 group, which were mostly due to mild to moderate injection-site reactions that are commonly associated with vaccines [10,14].

In conclusion, ASP0113 was not efficacious in the reduction of overall mortality and CMV EOD, and did not appear to be effective at reducing CMV viraemia through 1 year post-transplant in allo-HCT recipients. In addition, ASP0113 did not elicit a potent T-cell or B-cell response compared with placebo, but was associated with a similar safety profile to that of placebo with the exception of injection-site-related TEAEs, which were more frequent in the ASP0113 group. Owing to a lack of efficacy, the development of ASP0113 has been terminated. Participants in this study will continue to be followed up for 5.5 years post-transplant for long-term safety assessments.

## 5 Contributors

All authors met the International Committee of Medical Journal Editors authorship criteria. All authors were equally involved in the design or conception of the study, the analysis and interpretation of the data and drafting and critically reviewing the publication. All authors provided written approval for the manuscript to be submitted to *EClinicalMedicine*. Dr Ljungman, Dr Bermudez, Dr Logan, Dr Kharfan-Dabaja, Dr Chevallier, Dr Martino, Dr Wulf, Dr Selleslag, Dr Kakihana, Dr Langston, Dr Lee, Dr Solano, Dr Okamoto, Dr Boeckh, Dr Wingard and Dr Maertens were equally involved in the acquisition of the data as investigators.

## Data sharing

Access to anonymised individual participant level data collected during the trial, in addition to supporting clinical documentation, is planned for trials conducted with approved product indications and formulations, as well as compounds terminated during development. Conditions and exceptions are described under the Sponsor Specific Details for Astellas on www.clinicalstudydatarequest.com. Study-related supporting documentation is redacted and provided if available, such as the protocol and amendments, statistical analysis plan, and clinical study report. Access to participant-level data is offered to researchers after publication of the primary manuscript (if applicable) and is available as long as Astellas has legal authority to provide the data. Researchers must submit a proposal to conduct a scientifically relevant analysis of the study data. The research proposal is reviewed by an Independent Research Panel. If the proposal is approved, access to the study data is provided in a secure data-sharing environment after receipt of a signed Data Sharing Agreement.

## Funding

The study sponsor, Astellas Pharma Global Development Inc., was involved in the design or conception of the study, the analysis and interpretation of the data and drafting and critically reviewing the publication. The corresponding author had full access to all the data in the study and had final responsibility for the decision to submit this final version for publication.

**Appendix**

**Appendix 1. Study protocol**

**Appendix 2. Study design**

CMV=cytomegalovirus; MA=myeloablative conditioning; pp65=phosphoprotein 65; RIC=reduced-intensity conditioning; rtPCR=reverse transcription polymerase chain reaction; SOC=standard of care.

## Declaration of Competing Interest

Dr Ljungman reports grants, personal fees and non-financial support from 10.13039/501100004948Astellas personal fees from Vical, during the conduct of the study; personal fees from AiCuris, grants from Merck, grants from Shire, outside the submitted work.

Dr Bermudez reports other from Astellas, during the conduct of the study.

Dr Logan reports other from Astellas, during the conduct of the study; other from Novartis, outside the submitted work.

Dr Kharfan-Dabaja reports other from Seattle Genetics, other from Alexion Pharmaceuticals, other from Incyte, personal fees from Daiichi Sankyo, personal fees from Pharmacyclics, outside the submitted work.

Dr Chevallier has nothing to disclose.

Dr Martino reports other from Astellas, during the conduct of the study.

Dr Wulf reports personal fees from Astellas, during the conduct of the study.

Dr Selleslag reports personal fees from Astellas Pharma Global Development, Inc., personal fees from GlaxoSmithKline, personal fees from MSD, personal fees from Pfizer, during the conduct of the study.

Dr Kakihana reports personal fees from Chugai Pharmaceutical Co. Ltd, personal fees from Kyowa Hakko Kirin, personal fees from Bristol-Myers Squibb, personal fees from Takeda Pharmaceutical Co., personal fees from Dainippon Sumitomo Pharma, outside the submitted work.

Dr Langston has nothing to disclose.

Dr Lee reports grants from Astellas, during the conduct of the study; grants from GSK, personal fees from MSD, personal fees from Pfizer, personal fees from Gilead, personal fees from SL Vaxigen, outside the submitted work.

Dr Solano reports grants from Astellas Pharma Global Development, Inc., during the conduct of the study; personal fees from Gilead, personal fees from Mitsubishi Tanabe Pharma, outside the submitted work.

Dr Okamoto reports grants, personal fees and other from Astellas Pharma, during the conduct of the study.

Dr Smith reports personal fees from Astellas Pharma, during the conduct of the study.

Dr Boeckh reports grants from Astellas Pharma Global Development Inc, during the conduct of the study; grants and personal fees from Gilead, grants and personal fees from Merck, grants and personal fees from Takeda, grants and personal fees from Vir Bio, personal fees from Allovir, personal fees from GlaxoSmithKline, personal fees from Moderna, personal fees from Oxford Immunotec, personal fees from Evrys Bio, personal fees from Helocyte, grants from Lophius Biosciences, outside the submitted work.

Dr Wingard reports personal fees from Astellas Pharma Global Development, Inc., during the conduct of the study; personal fees from Allovir, personal fees from Ansun, personal fees from Celgene, personal fees from Cidara, personal fees from Merck, personal fees from Pluristem, personal fees from Shire, outside the submitted work.

Dr Cywin reports personal fees from Astellas, during the conduct of the study, and is an employee of Astellas.

Dr Fredericks reports personal fees from Astellas, during the conduct of the study; personal fees from Astellas, outside the submitted work; and is an employee of Astellas.

Dr Lademacher reports personal fees from Astellas, during the conduct of the study, and is an employee of Astellas.

Dr Wang reports other from Astellas, during the conduct of the study; other from Astellas Pharma global Development, outside the submitted work; and employment by Astellas.

Dr Young reports personal fees from Astellas, during the conduct of the study, and is an employee of Astellas.

Dr Maertens reports personal fees and non-financial support from 10.13039/501100004948Astellas, grants, personal fees and non-financial support from 10.13039/100004334Merck, personal fees and non-financial support from 10.13039/100004319Pfizer, personal fees and non-financial support from Basilea, personal fees and non-financial support from F2G, personal fees and non-financial support from 10.13039/100010643Cidara, grants, personal fees and non-financial support from 10.13039/100005564Gilead, outside the submitted work.

## Appendix Supplementary materials

Image, application 1

Image, image 2
