# Supplementary material for: A randomised, placebo-controlled phase 3 study to evaluate the efficacy and safety of ASP0113, a DNA-based CMV vaccine, in seropositive allogeneic haematopoietic cell transplant recipients
Source: eClinicalMedicine. 2021 Mar 19;33:100787. doi: 10.1016/j.eclinm.2021.100787 (PMC8020145; doi:10.1016/j.eclinm.2021.100787)
Supplement: Supplementary file 1 [file mmc1.pdf]

A Randomized, Double-Blind, Placebo-Controlled, Phase 3 Trial to Evaluate the Protective Efficacy and Safety of a Therapeutic Vaccine, ASP0113, in Cytomegalovirus (CMV)-Seropositive Recipients Undergoing Allogeneic, Hematopoietic Cell Transplant (HCT)

ISN/Protocol 0113-CL-1004

ClinicalTrials.gov Identifier: NCT01877655

Date of Protocol v6.2 [JP]: 12 Mar 2018

Sponsor: Astellas Pharma Global Development, Inc. (APGD)

Research & Development
1 Astellas Way
Northbrook, IL 60062

A Randomized, Double-Blind, Placebo-Controlled, Phase 3 Trial to Evaluate the Protective Efficacy and Safety of a Therapeutic Vaccine, ASP0113, in Cytomegalovirus (CMV)-Seropositive Recipients Undergoing Allogeneic, Hematopoietic Cell Transplant (HCT)

Protocol for Phase 3 Study of ASP0113

ISN/Protocol 0113-CL-1004

Version 6.2 [JP]

Incorporating Country-specific NonSubstantial Amendment 2

[See Attachment 1]

12 March 2018

IND 11,381

EudraCT 2013-000903-18

Sponsor:

Astellas Pharma Global Development, Inc. (APGD)
Research & Development
1 Astellas Way
Northbrook, IL 60062

Protocol History:

Version 1.0 Original [27Mar2013]
Version 2.0 [JP] Incorporating Country-Specific Substantial Amendment 1 [01Oct2013]
Version 3.0 (Global) Incorporating Substantial Amendment 2 [28Mar2014]
Version 4.0 [JP] Incorporating Country-Specific Substantial Amendment 3 [20May2014]
Version 5.0 (Global) Incorporating Substantial Amendment 4 [04Dec2015]
Version 6.0 (Global) Incorporating Substantial Amendment 5 [04Aug2016]
Version 6.1 (Global) Incorporating Non-Substantial Amendment 6 [28Oct2016]

This confidential document is the property of the Sponsor. No unpublished information contained in this document may be disclosed without prior written approval of the Sponsor.

Table of Contents

I. SIGNATURES **8**
II. CONTACT DETAILS OF KEY SPONSOR PERSONNEL **11**
III. LIST OF ABBREVIATIONS AND KEY TERMS **13**
IV. SYNOPSIS **18**
V. FLOW CHART AND SCHEDULE OF ASSESSMENTS **28**
1 INTRODUCTION **35**
1.1 Background 35
1.2 Product Composition 36
1.3 Nonclinical and Clinical Data 36
1.3.1 Nonclinical Data 36
1.3.2 Clinical Data 37
1.3.2.1 Phase 1 (VCL-CB01-101) Results 37
1.3.2.2 Phase 2 (CB01-202) Results 38
1.4 Summary of Key Safety Information for Study Drugs 40
1.5 Risk-Benefit Assessment 40
2 STUDY OBJECTIVES, DESIGN AND VARIABLES **41**
2.1 Study Objectives 41
2.2 Study Design and Dose Rationale 41
2.2.1 Study Design 41
2.2.2 Dose Rationale 44
2.3 Variables 45
2.3.1 Primary Variable 45
2.3.2 Secondary Variables 45
2.3.3 Exploratory Variables 45
2.3.4 Safety Assessments 46
2.3.5 Pharmacogenomics (PGx) Assessment (Optional) 46
3 STUDY POPULATION **46**
3.1 Selection of Study Population 46
3.2 Inclusion Criteria 47
3.3 Exclusion Criteria 49
3.4 Contraception and Pregnancy Avoidance 50
3.5 Discontinuation Criteria for Individual Subjects 50

4 STUDY PRODUCTS 52
4.1 Description of Study Products 52
4.1.1 Test Product 52
4.1.2 Comparative Product 52
4.2 Packaging and Labeling 52
4.3 Study Drug Handling 52
4.4 Blinding 54
4.4.1 Blinding Method 54
4.4.2 Retention of the Assignment Schedule and Procedures for Treatment Code Breaking 54
4.4.3 Breaking the Treatment Code for Emergency 55
4.4.4 Breaking the Treatment Code by the Sponsor 55
4.5 Assignment and Allocation 55
5 TREATMENTS AND EVALUATION 56
5.1 Dosing and Administration of Study Drugs and Other Medications 56
5.1.1 Dose/Dose Regimen and Administration Period 56
5.1.2 Increase or Reduction in Dose of the Study Drugs 56
5.1.3 Treatment Compliance 57
5.1.4 Emergency Procedures and Management of Overdose 57
5.1.5 Previous, Concomitant and Prohibited Medication (Drugs and Therapies) 57
5.1.5.1 Previous Medication (Drugs and Therapies) 57
5.1.5.2 Concomitant Medication (Drugs and Therapies) 57
5.1.5.3 Prohibited Concomitant Medications (Drugs and Therapies) 58
5.2 Demographics and Baseline Characteristics 58
5.2.1 Demographics 58
5.2.2 Medical History 58
5.2.3 Transplant Information 59
5.2.4 Modified HCT-CI Score 59
5.3 Efficacy and Immunogenicity Assessments 59
5.3.1 CMV Plasma Viral Load Assessment 59
5.3.2 Graft-Versus-Host Disease 60
5.3.3 CMV EOD 61
5.3.4 Infection 61
5.3.5 Cause of Death 61
5.3.6 Engraftment 61

5.3.6.1 Failure to Engraft ... 61
5.3.6.2 Rejection/poor Graft Function ... 61
5.3.6.3 Graft Failure ... 61
5.3.6.4 Platelet Recovery ... 61
5.3.6.5 Incidence of Relapse of Primary Disease Requiring Therapy ... 61
5.3.7 Immunogenicity ... 62
5.3.8 Resource Utilization and Patient Reported Outcomes ... 62
5.3.8.1 EQ-5D ... 63
5.3.8.2 FACT-BMT ... 63
5.3.8.3 Work Productivity and Activity Impairment: General Health Questionnaire ... 63
5.3.8.4 Health Economic Assessment ... 63
5.3.9 Optional Donor Sub-Study (Not Applicable to Sites in Japan) ... 63
5.4 Safety Assessment ... 64
5.4.1 Vital Signs ... 64
5.4.2 Adverse Events ... 64
5.4.2.1 Local Reactogenicity Assessments ... 64
5.4.3 Laboratory Assessments ... 65
5.4.3.1 Abnormal LFTs ... 66
5.4.3.2 Bronchoalveolar Lavage Fluid ... 66
5.4.4 Physical Examination ... 66
5.4.5 Long-Term Follow-up ... 66
5.5 Adverse Events and Other Safety Aspects ... 67
5.5.1 Definition of Adverse Events ... 67
5.5.2 Adverse Events of Special Interest ... 67
5.5.2.1 Malignancy ... 67
5.5.2.2 Sinusoidal Obstruction Syndrome (SOS) [Hepatic Veno-occlusive Disease (VOD)] ... 67
5.5.2.3 Adverse Events Commonly Associated with Vaccines ... 68
5.5.3 Definition of SAEs ... 68
5.5.4 Criteria for Causal Relationship to the Study Drug ... 69
5.5.5 Criteria for Defining the Severity of an Adverse Event ... 69
5.5.6 Reporting of SAEs ... 70
5.5.7 Follow-up to Adverse Events ... 71
5.5.8 Monitoring of Common Serious Adverse Events and Endpoint Events ... 71
5.5.9 Procedure in Case of Pregnancy ... 72

5.5.10 Supply of New Information Affecting the Conduct of the Study 73
5.5.11 Deviations from the Protocol and Other Actions Taken to Avoid Life-Threatening Risks to Subjects (FOR SITES IN JAPAN ONLY) 73
5.6 Test Drug Concentration 73
5.7 Other Measurements, Assessments, or Methods 74
5.7.1 Pharmacogenomics (PGx) 74
5.8 Total Amount of Blood 74
6 DISCONTINUATION 74
6.1 Discontinuation of Individual Subject(s) 74
6.2 Discontinuation of the Study 74
7 STATISTICAL METHODOLOGY 75
7.1 Sample Size 76
7.2 Analysis Set 76
7.2.1 Safety Analysis Set 76
7.2.2 Full Analysis Set 76
7.2.3 Per Protocol Set 76
7.2.4 Pharmacokinetic Analysis Set 77
7.2.5 Immunogenicity Analysis Set 77
7.3 Demographics and Other Baseline Characteristics 77
7.4 Analysis of Efficacy 77
7.4.1 Analysis of Primary Variable 77
7.4.1.1 Primary Analysis 77
7.4.1.2 Secondary Analysis for the Primary Endpoint 78
7.4.1.3 Subgroup Analysis 78
7.4.2 Analysis of Secondary Variables 78
7.4.2.1 Key Secondary Variables 78
7.4.2.2 Other Secondary Variable 79
7.4.3 Analysis of Other Variables 79
7.5 Analysis of Safety 80
7.6 Analysis of Pharmacokinetics 82
7.7 Analysis of Pharmacogenomics 82
7.8 Protocol Deviations and Other Analysis 82
7.9 Interim Analyses and Early Discontinuation of the Clinical Study 82
7.9.1 DMC 82

7.10 Handling of Missing Data, Outliers, Visit Windows, and Other Information ········83
8 OPERATIONAL AND ADMINISTRATIVE CONSIDERATIONS ··················83
8.1 Procedure for Clinical Study Quality Control ·······································83
8.1.1 Data Collection ···83
8.1.2 Specification of Source Documents ··83
8.1.3 Clinical Study Monitoring ··84
8.1.4 Direct Access to Source Data/Documents ·····································84
8.1.5 Data Management ···84
8.1.6 Protocol Deviations ···84
8.1.7 End of Trial in All Participating Countries ··································85
8.2 Ethics and Protection of Subject Confidentiality ·································85
8.2.1 IRB / IEC / CA ··85
8.2.2 Ethical Conduct of the Study ··86
8.2.3 Informed Consent of Subjects ··86
8.2.3.1 Subject Information and Consent ··86
8.2.3.2 Supply of New and Important Information Influencing the Subject's Consent and Revision of the Written Information ···························86
8.2.4 Subject Confidentiality ···87
8.3 Administrative Matters ··88
8.3.1 Arrangement for Use of Information and Publication of the Clinical Study ·····88
8.3.2 Documents and Records Related to the Clinical Study ··························88
8.3.3 Protocol Amendment and/or Revision ··90
8.3.4 Insurance of Subjects and Others (FOR JAPAN SITES ONLY) ·················90
8.3.5 Signatory Investigator for Clinical Study Report ·····························91
9 QUALITY ASSURANCE ··91
10 STUDY ORGANIZATION ··91
10.1 Data Monitoring Committee (DMC) ··91
10.2 Adjudication Committee ···91
10.3 Other Study Organization ··92
10.3.1 Home Healthcare ···92
11 REFERENCES ··93
12 APPENDICES ··96
12.1 Appendix 1: Laboratory Tests ··96
12.2 Appendix 2: Definition of CMV Disease ··98

12.3 Appendix 3: Common Serious Adverse Events and Endpoint Events ... 99
12.4 Appendix 4: Protocol Defined Definitions of Comorbidities for the HCT-Comorbidity Index ... 100
12.5 Appendix 5: Dynamic International Prognostic Scoring System for Survival in Primary Myelofibrosis ... 103
12.6 Appendix 6: Definition of Morphologic Complete Remission ... 104
12.7 Appendix 7: Grading of Reactogenicity ... 105
12.8 Appendix 8: Severity Grading Table and Recurrence Interval Definitions for Infections ... 106
12.9 Appendix 9: Staging and Grading of Acute Graft versus Host Disease ... 110
12.10 Appendix 10: Rule of Nines Burn Chart ... 112
12.11 Appendix 11: Criteria for the Diagnosis of Chronic Graft Versus Host Disease ... 113
12.12 Appendix 12: Grading of Chronic Graft versus Host Disease ... 117
12.13 Appendix 13: Organ Scoring and Global Scoring of Chronic Graft versus Host Disease ... 118
12.14 Appendix 14: EQ-5D Questionnaire ... 121
12.15 Appendix 15: FACT-BMT (Version 4) ... 123
12.16 Appendix 16: Work Productivity and Activity Impairment Questionnaire: General Health Version 2.0 ... 126
12.17 Appendix 17: Definitions of Conditioning Regimens ... 128
12.18 Appendix 18: Liver Safety Monitoring and Assessment ... 129
12.19 Appendix 19: Pharmacogenomics (PGx) Sub-Study ... 131
12.20 Appendix 20: Long-term Follow-up Questionnaire ... 133
12.21 Appendix 21: Health Economic Assessment (Inpatient and Outpatient Utilization) ... 134
12.22 Appendix 22: Karnofsky Performance Scale ... 135
13 ATTACHMENT 1: COUNTRY-SPECIFIC NONSUBSTANTIAL AMENDMENT 2 [JP] ... 136
14 SPONSOR’S SIGNATURES ... 139

I. SIGNATURES

1a. SPONSOR'S SIGNATURE

Required signatures (e.g., Protocol authors, Sponsor's reviewers and contributors, etc.) are located in Section 14 Sponsor's Signatures; e-signatures (when applicable) are located at the end of this document.

1b. AGREEMENT BETWEEN THE SPONSOR'S RESPONSIBLE PERSON AND THE INVESTIGATOR (for sites in Japan)

This clinical study will be conducted in adherence to GCP, ICH Guidelines and applicable laws and regulatory requirements, as well as this study protocol. As the evidence of the agreement, the Investigator (CHIKEN SEKININ ISHI) and responsible person of the Sponsor (CHIKEN IRAI SEKININSHA) inscribe in the bipartite agreement.

2. COORDINATING INVESTIGATOR'S SIGNATURE

A Randomized, Double-Blind, Placebo-Controlled, Phase 3 Trial to Evaluate the Protective Efficacy and Safety of a Therapeutic Vaccine, ASP0113, in Cytomegalovirus (CMV)-Seropositive Recipients Undergoing Allogeneic, Hematopoietic Cell Transplant (HCT)

ISN/Protocol 0113-CL-1004

Version 6.2 [JP] / Incorporating Country-specific Nonsubstantial Amendment 2

12 March 2018

I have read all pages of this clinical study protocol for which Astellas is the Sponsor. I agree that it contains all the information required to conduct this study.<br>**Coordinating Investigator:**
Signature: ______________________ ______________ Date<br>Printed Name: ______________________<br>Address: ______________________<br>______________________<br>______________________

3. INVESTIGATOR'S SIGNATURE

A Randomized, Double-Blind, Placebo-Controlled, Phase 3 Trial to Evaluate the Protective Efficacy and Safety of a Therapeutic Vaccine, ASP0113, in Cytomegalovirus (CMV)-Seropositive Recipients Undergoing Allogeneic, Hematopoietic Cell Transplant (HCT)

ISN/Protocol 0113-CL-1004

Version 6.2 [JP] / Incorporating Country-specific Nonsubstantial Amendment 2

12 March 2018

I have read all pages of this clinical study protocol for which Astellas is the Sponsor. I agree to conduct the study as outlined in the protocol and to comply with all the terms and conditions set out therein. I confirm that I will conduct the study in accordance with ICH GCP guidelines and applicable local regulations. I will also ensure that sub-Investigator(s) and other relevant members of my staff have access to copies of this protocol and the ICH GCP guidelines to enable them to work in accordance with the provisions of these documents.
**Principal Investigator:**<br><br>Signature: --<br>Date<br>Printed Name: --<br>Address: --<br>--<br>--

II. CONTACT DETAILS OF KEY SPONSOR PERSONNEL

**24h-Contact for Serious Adverse Events (SAEs)**<br><br>**See [Section 5.5.6]**	PPD **MD**<br>PPD<br>PPD<br><br>*Country specific toll free numbers will be available.*<br><br>**All countries except Japan:**<br>**Please fax or email the SAE Worksheet to:**<br>**Astellas Pharma Global Development, Inc.**<br>**Product Safety & Pharmacovigilance**<br>**Fax number: +1 847-317-1241**<br>**International Fax: +44 800-471-5263**<br>**Email: Safety-US@astellas.com**<br><br>**For sites in Japan only:**<br>**Please fax the JUTOKUNA YUUGAIJISHOU HOUKOKUSHO to the delegated CRO:**<br>PPD
Medical Monitor/Medical Expert	PPD MD, PhD<br>PPD, Medical & Development<br>Astellas Pharma Global Development, Inc.<br>PPD
Clinical Research Contacts:	PPD<br>PPD<br>Global Clinical Science,<br>Astellas Pharma Global Development, Inc.<br>PPD<br><br>PPD<br>PPD, Clinical Program Management,<br>Global Clinical Science,<br>Astellas Pharma Global Development, Inc.<br>PPD

	PPD<br>For sites in Japan only:<br>PPD<br>PPD<br>Japan/Asia Clinical Development 1,<br>Astellas Pharma Inc.<br>PPD

For Study Sites in Japan:

Contact Information for the Sponsor

Corporate Name: Japan/Asia Clinical Development 1,

Global Development, Astellas Pharma Inc.

Location: 2-5-1, Nihonbashi-Honcho, Chuo-ku, Tokyo

PPD

III. LIST OF ABBREVIATIONS AND KEY TERMS

List of Abbreviations

Abbreviations	Description of Abbreviations
AC	Adjudication Committee
ADL	Activities of Daily Living
ADR	Adverse Drug Reactions
AE	Adverse Event
aGVHD	Acute Graft-versus-host Disease
ALC	Absolute Lymphocyte Count
ALL	Acute Lymphocytic Leukemia
ALT	Alanine Aminotransferase (GPT)
AML	Acute Myelogenous Leukemia
ANC	Absolute Neutrophil Count
AST	Aspartate Aminotransferase (GOT)
ASP0113	DNA vaccine being tested in this study
AUL	Acute Undifferentiated Leukemia
AUST	Astellas US Technologies, Inc.
AVT	Antiviral Therapy
BAK	Benzalkonium Chloride
BAL	Bronchoalveolar Lavage
BMT	Bone Marrow Transplant
BMTS	Bone Marrow Transplantation Subscale
CA	Competent Authorities
cGVHD	Chronic Graft-versus-host Disease
CIOMS	Council for International Organizations of Medical Sciences
CMH	Cochran-Mantel-Haenszel
CMV	Cytomegalovirus
CPK	Creatine Phosphokinase
CR	Complete Remission
CRO	Contract Research Organization
DL_{CO}	Diffusing Lung Capacity of Carbon Monoxide
DMC	Data Monitoring Committee
EBV	Epstein-Barr Virus
ECG	Electrocardiogram
eCRF	Electronic Case Report Form
EOD	End Organ Disease
EQ-5D	EuroQol-5D
ER	Emergency Room
FACT-BMT	Functional Assessment of Cancer Therapy – Bone Marrow Transplant
FAS	Full Analysis Set
FEV1	Forced Expiratory Volume
gB	Glycoprotein B

Abbreviations	Description of Abbreviations
G-CSF	Granulocyte colony stimulating factor
GCP	Good Clinical Practice
GVHD	Graft-versus-host Disease
H_0	Null Hypothesis
H_1	Alternative Hypothesis
HCT	Hematopoietic Cell Transplant
HCT-CI	Hematopoietic Cell Transplant Comorbidity Index
HEA	Health Economics Assessment
HHC	Home Healthcare
HIPAA	Health Insurance Portability and Accountability Act
HIV	Human Immunodeficiency Virus
HLA	Human Leukocyte Antigen
IAS	Immunogenicity Analysis Set
IB	Investigator’s Brochure
ICF	Informed Consent Form
ICH	International Conference on Harmonization of Technical Requirements for Registration of Pharmaceuticals for Human Use
ICS	Intracellular Cytokine Staining
IEC	Independent Ethics Committee
IFN-γ	Interferon γ
IgG	Immunoglobulin G
IND	Investigational New Drug
IRB	Institutional Review Board
ISN	International Study Number
ITT	Intent-to-treat
IU	International Unit
KPS	Karnofsky Performance Scale
LFT	Liver Function Tests
MDS	Myelodysplastic Syndrome
MHC	Major Histocompatibility Complex
MUGA	Multi gated Acquisition Scan
NCI-CTCAE	National Cancer Institute Common Terminology Criteria for Adverse Events
PB	Privacy Board
PBS	Phosphate-buffered Saline
PCR	Polymerase Chain Reaction
pDNA	Plasmid DNA
PE	Physical Examination
PFT	Pulmonary Function Test
PGx	Pharmacogenomics
PHI	Protected Health Information
PP	Per Protocol

Abbreviations	Description of Abbreviations
pp65	Phosphoprotein 65
PPS	Per Protocol Set
QOL	Quality of Life
RBC	Red Blood Cell
SAE	Serious Adverse Event
SAF	Safety Analysis Set
SAP	Statistical Analysis Plan
SOP	Standard Operating Procedure
SUSAR	Suspected Unexpected Serious Adverse Reaction
TEAE	Treatment-emergent Adverse Event
ULN	Upper Limit of Normal
VE	Vaccine Efficacy
WPAI:GH	Work Productivity and Activity Impairment Questionnaire: General Health

List of Key Study Terms

Terms	Definition of terms
Adverse event	An adverse event is any untoward medical occurrence in a subject administered a study drug or has undergone study procedures and which does not necessarily have a causal relationship with the treatment.
Adverse reaction	An adverse reaction means any adverse event caused by a drug.
Baseline	Baseline is the time point prior to the first dose of randomized study drug. Visit 2 is considered the baseline visit in this study. The last measurement / evaluation prior to the first dose of randomized therapy are considered the baseline measure/evaluation.
CMV infection	Replication of CMV (isolation of the virus or detection of viral protein or nucleic acid in any body fluid or tissue)
CMV end organ disease (EOD)	As defined in [Appendix 2]
CMV viremia	Presence of CMV in the blood
End of study	The point in time when the last protocol-defined assessment has been completed (end of the long-term follow-up period)
End of treatment	The point in time when the subject receives the last dose of study drug
Enrollment	The point in time when the subject signs the informed consent
Evaluable subject	A subject who meets all inclusion criteria and does not meet any exclusion criteria and who has received at least 1 injection of study drug
Long-term Follow-up Period	The period of time from the day 365 visit (V14) through the completion of the 4½ year additional safety follow-up
Long-term follow-up withdrawal	A subject who completes the primary study period but does not complete the long-term follow-up period for any reason
Preemptive therapy	A therapeutic treatment regimen where treatment for an infection/disease is initiated only after it is detected/confirmed
Primary follow-up period	The period of time from the end of the study drug treatment through the day 365 visit (V14)
Primary study period	The period of time from enrollment (signing of informed consent) through the day 365 visit (V14)
Primary study withdrawal	A subject who is randomized but does not complete the primary study period for any reason
Principal investigator	A physician responsible for the conduct of the clinical trial at a trial site. If a trial is conducted by a team of individuals at a trial site, the Principal Investigator is the responsible leader of the team.
Prophylactic therapy	A treatment regimen where treatment is administered prior to the detectable presence of infection or disease as a preventative measure

Terms	Definition of terms
Randomization	The action to allocate a subject to a treatment group. In this study, randomization occurs after the subject has met all inclusion and exclusion criteria between days -3 to -14 prior to the transplant date.
Screening	The process for identifying a candidate for the study and evaluating their eligibility to participate
Screen failure	A subject who signs the informed consent and undergoes the protocol-specific screening procedures, but does not fulfill the protocol inclusion and/or exclusion criteria. This subject is not to be randomized.
Serious adverse event	An adverse event is considered "serious" if, in the view of either the Investigator or Sponsor, it results in any of the following outcomes: results in death, is life threatening, results in persistent or significant disability / incapacity or substantial disruption of the ability to conduct normal life functions, results in congenital anomaly or birth defect, requires inpatient hospitalization or leads to prolongation of hospitalization, or is a medically important event.
Source data	All information in original records and certified copies of original records of clinical findings, observations, or other activities in a clinical trial necessary for the reconstruction and evaluation of the trial. Source data are contained in source documents.
Source documents	Original documents, data and records including source data
Subject	An individual who participates in a clinical trial
Treatment period	The period of time from the first dose of study drug through the last dose of study drug

IV. SYNOPSIS

**Title of Study**	A Randomized, Double-Blind, Placebo-Controlled, Phase 3 Trial to Evaluate the Protective Efficacy and Safety of a Therapeutic Vaccine, ASP0113, in Cytomegalovirus (CMV)-Seropositive Recipients Undergoing Allogeneic, Hematopoietic Cell Transplant (HCT)
**Planned Study Period:**	Primary Study Period: From 3Q2013 to 3Q2017<br>Long-Term Follow-up Period: until 1Q2022
**Study Objectives**	• To evaluate the efficacy of ASP0113 compared with placebo as measured by a primary composite endpoint of overall mortality and CMV end organ disease (EOD) through 1 year post-transplant.<br>• To evaluate the safety of ASP0113 in subjects undergoing allogeneic HCT.
**Planned Total Number of Study Centers and Location**	Approximately 90 sites globally<br>Countries may include, but are not limited to, Australia, Belgium, Canada, France, Germany, Japan, South Korea, Spain, Sweden, Taiwan and United States
**Design and Methodology**	This is a randomized, double-blind, placebo-controlled trial of approximately 500 cytomegalovirus (CMV)-seropositive recipients undergoing allogeneic, hematopoietic cell transplant (HCT). Subjects will be randomized in a 1:1 ratio, ASP0113 or placebo, and stratified by donor-recipient relatedness and donor CMV serostatus. At least 30% of subjects enrolled will be donor CMV-seronegative.<br>The test compound will be a vaccine (ASP0113) which contains 2 plasmids encoding glycoprotein B (gB) and phosphoprotein 65 (pp65), each at 2.5 mg/mL, formulated with CRL1005 poloxamer and benzalkonium chloride (BAK). The placebo control will be phosphate-buffered saline (PBS).<br>Subjects will receive either 5 doses of ASP0113 or placebo on days -14 to -3, 14-40, 60 ±5, 90 ±10 and 180 ±10 in relation to the day of transplant (donor cell infusion).<br>The first injection must be given within 72 hours prior to the start of chemo-therapy and radiation therapy for conditioning and the second injection is to be given as close as possible to, but not prior to, day 14.<br>The platelet count must be ≥ 50000 mm^3 (spontaneously or after platelet transfusion) performed by the local laboratory within 3 days prior to all study drug injections. Prior to all study drug injections, confirmation must be made that there is no medical contraindication to an IM injection. Results received in an equivalent local unit of measure must be converted to SI units. Syringes will be masked prior to dosing to blind the subjects and all other personnel who need to remain blinded to the treatment assignment (i.e., site staff other than pharmacy personnel and staff designated to administer study drug injections).<br>CMV plasma viral load will be monitored per the following schedule during the primary study period (through day 365/V14): weekly (±2 days) during days 0-100, every other week (± 5 days) during days 101-180, every 30 days (±5 days) during days 181-365. The CMV plasma viral loads will be performed by the central laboratory. CMV plasma viral load will also be performed by the

**Design and Methodology** continued	central laboratory at the initiation of CMV-specific antiviral therapy (AVT). Upon initiation of CMV-specific AVT, plasma viral load testing will be performed at least weekly until the CMV-specific AVT is discontinued, at which point, the regularly scheduled viral load assessments will resume.<br><br>**Note:** CMV plasma viral loads may be performed at a local laboratory approved by the Sponsor and at the discretion of the Investigator. However, every time a viral load sample is sent to the local laboratory a sample will also be sent to the central laboratory for confirmatory testing. Pre-emptive therapy may be started based on the central or local laboratory assessment and the subject's clinical condition.<br><br>Adjudicated CMV-specific AVT will be assessed for subjects up to1 year following transplant through the primary study period (through day 365/V14).<br><br>Immunologic response will be assessed in all subjects at V2, V5, V8, V9, V11 and V14. If the visit coincides with a study drug administration, the sample will be drawn prior to injection.<br><br>In addition, if a CMV seropositive donor has consented and is participating in the optional donor cell sub-study, a 20 mL peripheral blood sample from the donor will be obtained prior to the stem cell donation for recipients participating in the main trial to test for CMV-specific T-cells. For those donating peripheral stem cells, the donor blood sample is to be collected prior to the initiation of mobilization therapy (e.g., G-CSF). This process is not applicable to sites in Japan.<br><br>Subjects will be evaluated for local reactogenicity and systemic signs for 60 minutes after each injection. Subjects will be directly observed for 15 minutes following the injection for any injection-related side effects, then remain in a designated area at the study site for 45 minutes so that side effects can be noted and treated, if necessary. Local reactogenicity will also be evaluated for each of the 7 consecutive days following each injection beginning approximately 24 hours after the dose (day 1-7 post dose). The assessments for each of the 7 consecutive days following an injection will be done by the subject and reported to the site via diary. When reactogenicity is reported as an AE, it must be followed until resolution or medically stable. All local reactions ≥ grade 3 [Appendix 7] require confirmation by a health care professional.<br><br>Subjects will be contacted after the last injection of vaccine through 5.5 years post-transplant for long-term safety related to the DNA vaccine. Items to be assessed include mortality, development of any new/ recurrent cancer, development of infection requiring hospitalization or resulting in death and erythema and/or induration at the injection sites [Appendix 20].<br><br>All AEs and SAEs will be collected from signing of the informed consent through 30 days post last dose of study drug. All events requiring adjudication, grade 3 and higher grade AEs as well as SAEs will be collected from 31 days post last dose of study drug through 1 year post-transplant. A change in medical status or medical history from time of signing of informed consent through first dose of study drug is to be reported as an AE or SAE, as appropriate.<br><br>An independent Data Monitoring Committee (DMC) will be chartered to oversee safety issues and futility analysis.

**Design and Methodology** continued	Futility analysis will be performed when the first 100 subjects have been randomized, and every 3 months thereafter until the total enrollment reaches 400 subjects. Futility analyses are based on the rate of CMV viremia defined as CMV plasma load ≥1000 IU/mL as assessed by the central laboratory. At each futility analysis, a Bayesian posterior probability that the hazard ratio for viremia of the ASP0113 treatment group to the placebo treatment group will be less than 0.75 will be calculated. The study may stop for futility if this posterior probability is less than 0.15.<br><br>An Adjudication Committee (AC) will be chartered to adjudicate all cases of CMV EOD, initiation of CMV-specific AVT, and cause of death.
**Number of Subjects Planned**	Approximately 500 subjects will be enrolled and receive study drug in the study.
**Selection Criteria**	*Inclusion Criteria:*<br><br>A subject is eligible for the study if all of the following apply:<br>1. Institutional Review Board (IRB)/Independent Ethics Committee (IEC)-approved written informed consent and privacy language as per national regulations (e.g., Health Insurance Portability and Accountability Act (HIPAA) Authorization for U.S. sites) must be obtained from the subject or legally authorized representative prior to any study-specific related procedures (including withdrawal of prohibited medication, if applicable).<br>2. Subject is a male or female subject who is at least 18 years old or the legal age of consent (whichever is greater).<br>3. Subject is a CMV-seropositive HCT recipient as confirmed by local laboratory at screening.<br>4. Subject is planned to undergo either of the following:<br>a. Sibling Donor Transplant – 7/8 Human Leukocyte Antigen (HLA)-A, -B, -C, -DRß1 match utilizing high resolution typing or 8/8 (HLA)-A, -B, -C, -DRß1 match utilizing low or high resolution typing.*<br>b. Unrelated Donor Transplant - 7/8 or 8/8 HLA-A, -B, -C, -DRß1 match utilizing high resolution typing.*<br>* A minimum of 8 allele matching is required. Subjects with matches performed at more than 8 alleles are included if they have 7/8 or 8/8 match at the HLA-A, -B, -C, –DRß1 alleles.<br>5. Subject is scheduled to receive an allogeneic peripheral blood stem cell or bone marrow transplant (BMT) for the treatment of hematologic disorders as indicated in Inclusion Criterion 6.<br>6. Subject has 1 of the following underlying diseases:<br>a. Acute myeloid leukemia (AML), with or without a history of myelodysplastic syndrome (MDS), in first or second complete remission [Appendix 6] or in early relapse (< 20% blasts in bone marrow with no circulating blasts in peripheral blood and no extramedullary leukemia).<br>b. Acute lymphoblastic leukemia (ALL), in first or second complete remission [Appendix 6]<br>c. Acute undifferentiated leukemia (AUL) in first or second complete remission [Appendix 6]

**Selection Criteria** continued	*Inclusion Criteria continued:*<br>d. Acute biphenotypic leukemia in first or second complete remission [Appendix 6]<br>e. Chronic myelogenous leukemia (CML) in either chronic or accelerated phase<br>f. Chronic lymphocytic leukemia.<br>g. One of the following MDS defined by the following:<br>i. Refractory anemia with evidence of dysplasia<br>ii. Refractory anemia with ringed sideroblasts<br>iii. Refractory cytopenia with multilineage dysplasia<br>iv. Refractory cytopenia with multilineage dysplasia and ringed sidero-blasts<br>v. Refractory anemia with excess blasts-1 (5%-10% blasts)<br>vi. Refractory anemia with excess blasts-2 (10%-20% blasts)<br>vii. MDS, unclassified<br>viii. MDS associated with isolated deletion (5q)<br>ix. Chronic myelomonocytic leukemia<br>h. Primary or secondary myelofibrosis without leukemic transformation except if Dynamic International Prognostic Scoring System category of high or intermediate -2 [Appendix 5]<br>i. Lymphoma (including Hodgkin's) with chemosensitive disease (≥ 50% response to chemotherapy)<br>7. Female subject must be either:<br>• Of non-childbearing potential:<br>o Postmenopausal (defined as at least 1 year without any menses) prior to screening, and if < 50 years of age and not documented to be surgically sterile must have a negative urine or serum pregnancy test at screening, or<br>o Documented surgically sterile or status post-hysterectomy (at least 1 month prior to screening)<br>• Or, if of childbearing potential:<br>o Must have a negative urine or serum pregnancy test at screening, and<br>If heterosexually active, must use at least 1 form of birth control* (which must be a barrier method) starting at screening and through the primary study period.<br>8. Female subject must not be breastfeeding at screening, through the treatment period and through the primary study period.<br>9. Female subject must not donate ova starting at screening, through the treatment period and through the primary study.<br>10. Male subject and their female spouse/partners who are of childbearing potential must be using highly effective contraception consisting of 2 forms of birth control* (1 of which must be a barrier method) starting at screening, through the treatment period and through the primary study period.

**Selection Criteria** continued	*Inclusion Criteria continued:*<br>* Acceptable forms include:<br>▪ Consistent and correct usage of established oral contraception.<br>▪ Established intrauterine device or intrauterine system.<br>▪ Barrier methods of contraception: condom or occlusive cap (diaphragm or cervical/vault caps) with spermicidal foam/gel/film/cream/suppository <Not applicable for sites in Japan: with spermicidal foam/gel/film/cream/suppository><br>▪ For sites in Japan: calendar-based contraceptive methods (Knaus-Ogino or rhythm method)<br>11. Male subject must not donate sperm starting at screening, through the treatment period and through the primary study period.<br>12. Subject is willing to comply with the protocol.<br>13. Subject agrees not to participate in another interventional study while on treatment. New regimens of approved chemotherapeutic drugs or antibodies, conditioning drugs, irradiation and T-cell depleting antibodies, except alemtuzumab, are allowed.<br>Waivers to the inclusion criteria will NOT be allowed.<br><br>*Exclusion Criteria:*<br>A subject will be excluded from participation if any of the following apply:<br>1. Subject has active CMV disease or infection or has received treatment for active CMV disease or infection within 3 months (90 days) prior to transplant.<br>2. Subject has planned CMV prophylactic therapy with antiviral drugs or CMV-specific immunoglobulins from randomization through the primary study period completion/day 365 visit (V14).<br>3. Subject has a modified hematopoietic cell transplant comorbidity index (HCT-CI) score ≥ 4 [Appendix 4].<br>4. Subject is known to be positive for human immunodeficiency virus (HIV), hepatitis B surface antigen or hepatitis C ribonucleic acid (RNA) polymerase chain reaction (PCR).<br>5. Donor CMV serostatus is unknown.<br>6. Subject has received any of the following substances or treatments:<br>• Alemtuzumab within 60 days prior to transplant, including conditioning regimen. Subjects for whom treatment with alemtuzumab is planned at any time from 60 days prior to through 1 year post-transplant must not be enrolled in the trial.<br>• T cell depletion of donor cell product.<br>• Administration of a CMV vaccine, including any prior exposure to ASP0113.<br>7. Subject has received an allogeneic stem cell transplant within 1 year prior to transplant (subjects who have received a prior autologous transplant are allowed).

**Selection Criteria** continued	8. Subject has a current malignancy in addition to the malignancy being treated for the study or the subject has a history of any other malignancy (within the past 5 years prior to screening) except non-metastatic basal or squamous cell carcinoma of the skin that has been treated successfully or cancer in situ of the cervix uteri that has been handled by local surgery.<br>9. Subject has an unstable medical or psychiatric condition, including a history of illicit drug(s) or alcohol abuse that the Investigator believes will interfere with protocol requirements.<br>10. Subject has had an allergic reaction to any component of the vaccine. Subject has had an allergic reaction to aminoglycosides as kanamycin is used in the manufacturing process of the vaccine.<br>11. Subject has participated in any interventional clinical study or has been treated with any investigational research products within 30 days or 5 half-lives, whichever is longer, prior to the initiation of screening. (Investigational research products are considered those products that have not been approved for any indication in the country where the subject is enrolled. New regimens of approved chemotherapeutic drugs or antibodies, conditioning drugs, irradiation and T-cell depleting antibodies, except alemtuzumab, are allowed.)<br>12. Subject has received a prior HCT and has residual cGVHD.<br>13. Subject who is scheduled to have a cord blood transplant or a haploidentical transplant.<br>14. Subject has a platelet count of less than 50000 mm^3 within 3 days prior to randomization (platelet transfusions are allowed). Results received in an equivalent local unit of measure must be converted to SI units.<br>15. Subject has aplastic anemia or multiple myeloma.<br>16. For sites in Japan only: Other subjects considered ineligible by the Investigator/sub-Investigator.<br>Waivers to the exclusion criteria will NOT be allowed.
**Primary Study Period Discontinuation Criteria**	<u>Study Discontinuation</u><br>Subjects will be withdrawn from study and will receive no further follow-up except for mortality (at day 365 post-transplant) through public record /sources if the following occurs:<br>• Withdrawal of consent by subject<br>Subjects will be withdrawn from the study and will receive no further follow-up if the following occurs:<br>• Subject did not receive first dose of study drug<br><u>Treatment Only Discontinuation</u><br>Subjects will be withdrawn from study drug treatment if any of the following occur; however, they are to continue to be followed according to the protocol Schedule of Assessments [Table 1] with exception of the dose follow-up visits (i.e., V3, V6, V8, V10, and V12).<br>• Investigator's decision that further treatment is not in the best interest of the subject.<br>• Pregnancy (female subjects only).<br>• Failure to engraft.

	• Local reactogenicity with a grade ≥ 3 based on the criteria defined in [Appendix 7] and confirmed by a health care professional.<br>• Anaphylaxis with a grade ≥ 3 based on National Cancer Institute Common Terminology Criteria for Adverse Events (NCI-CTCAE) Version 4.03.<br>• Seizure considered possibly or probably related to study drug treatment.<br>• Participation in another interventional trial with an investigational research product (investigational research products are considered those products that have not been approved for any indication in the country where the subject is enrolled).<br>If a subject is withdrawn from study drug treatment and is unable to comply with study procedures as defined in the Schedule of Assessments, the subject must be asked if they are willing to be followed for SAEs through 30 days of last dose of study drug treatment, to attend the scheduled final study visit (V14), to be assessed for CMV EOD, and/or to be followed for mortality. They must also be asked if they are willing to be followed through the long term follow-up period.<br>Subjects who discontinue from the study after one or more doses of study drug will not be replaced. Subjects who are randomized but never receive study drug will be replaced, to a total of approximately 500 dosed subjects.
**Test Drug Dose**<br>**Mode of Administration**<br>**Duration of Treatment**	ASP0113 will be dosed as 1 mL administered IM in the deltoid muscle with a needle and syringe, alternating sides with each dose, if possible.<br>Subjects will receive 5 injections of ASP0113 over an approximate 6 month period.
**Reference Drug Dose**<br>**Mode of Administration**<br>**Duration of Treatment**	Placebo is PBS and will be dosed as 1 mL administered intramuscularly in the deltoid muscle with a needle and syringe, alternating sides with each dose, if possible.<br>Subjects will receive 5 injections of placebo over an approximate 6 month period.
**Concomitant & Prohibited Medications & Therapies**	<u>Concomitant Medications & Therapies</u><br>*Conditioning Regimens*<br>Subjects are to undergo a conditioning regimen that is standard of care for the institution at which they are enrolled. Both myeloablative and non-myeloablative (reduced intensity) conditioning regimens are allowed. Note that alemtuzumab is not allowed within 60 days prior to transplant.<br>*Viral Prophylaxis*<br>Viral prophylaxis for infection other than CMV is to be administered according to standard institutional protocols. Prophylactic use of aciclovir (acyclovir), valaciclovir (valacyclovir), or famciclovir must not exceed the following doses following transplant (day 0) through 1 year post-transplant (day 365/V14) [Kumar, 2011; Tomblyn, 2009].<br>*Aciclovir:*<br>1600 mg orally (total daily dose), or 500 mg/m^2/day IV* (total daily dose).<br>*If dose is IV it may be rounded up to the nearest 100 mg.<br>*Valaciclovir:* 1000 mg po (total daily dose).

<table>
<tr><td></td><td>Famciclovir: 500 mg po (total daily dose).
These doses may NOT be exceeded for prophylaxis but may be exceeded if necessary to treat active infections post-transplant.</td></tr>
<tr><td></td><td><u>Prohibited Concomitant Medications (Drugs and Therapies)</u>
The following medications and therapies are prohibited throughout the study [through the primary study period completion/day 365 visit (V14)]:
● Investigational research products that have not been approved for any indication in the country where the subject is enrolled.
o New regimens of approved chemotherapeutic drugs or antibodies, conditioning drugs, irradiation and T-cell depleting antibodies, except alemtuzumab, are allowed.
● Prophylactic use of antiviral drugs for CMV viremia from time of Randomization through completion of the primary study period completion/ day 365 visit (V14).
● Prophylactic use of CMV specific immunoglobulin.
● Alemtuzumab within 60 days prior to transplant (including conditioning regimen) through completion of the primary study period completion/day 365 visit (V14) unless required for disease relapse post-transplant.</td></tr>
<tr><td>Primary Variables</td><td>The primary endpoint of the study is the composite of overall mortality and CMV EOD through one year post-transplant.</td></tr>
<tr><td>Secondary Variables</td><td>Key secondary endpoints:
● Time to first protocol-defined CMV viremia [CMV plasma viral load ≥ 1000 IU/mL as assessed by the central laboratory] through 1 year post-transplant.
● Time to first adjudicated CMV-specific AVT through 1 year post-transplant.
Additional secondary endpoint:
● Time to first CMV-specific AVT for protocol-defined CMV viremia[CMV plasma viral load ≥ 1000 IU/mL as assessed by the central laboratory] or CMV EOD through one year post-transplant.</td></tr>
<tr><td>Exploratory Variables</td><td>● Clinical outcomes:
➢ Overall mortality
➢ Incidence of CMV EOD
➢ Incidence of grade 3-4 aGVHD
➢ Incidence of severe cGVHD
➢ Incidence of grade 3 treatment-emergent infections other than CMV
➢ Maximum grade of aGVHD
➢ Maximum cGVHD score
➢ Relapse mortality (death due to relapse of the subject's primary disease)
➢ Non-relapse mortality (death due to causes unrelated to the subject's primary disease)
➢ Incidence of graft rejection
➢ Incidence of failure to engraft
➢ Time to engraftment
➢ Time to platelet recovery</td></tr>
</table>

	➢ Incidence of relapse of primary disease requiring therapy<br>➢ Number of episodes of protocol-defined CMV viremia [CMV plasma viral load ≥ 1000 IU/mL as assessed by the central laboratory]<br>➢ Time to grade 3 treatment-emergent viral, bacterial, or fungal infections other than CMV<br>• Duration of hospitalization(s)<br>• Health economic/resource utilization<br>• Subjects' quality of life (EQ-5D, FACT-BMT, and Work Productivity and Activity Impairment Questionnaire: General Health [WPAI: GH])<br>• Immunogenicity Variables<br>➢ T-cell responses to pp65<br>➢ gB-specific antibody levels
**Safety Assessments**	• Vital signs<br>• Adverse events<br>• Local reactogenicity signs and symptoms using both the NCI-CTCAE Version 4.03 grading scale and the protocol-specified reactogenicity scale [Appendix 7]<br>• Clinical laboratory assessments as defined in [Appendix 1]<br>• Physical examination (PE)
**Statistical Methods**	This is a randomized, double-blind, placebo-controlled trial of approximately 500 subjects. In the primary analysis set of a phase 2 study of the efficacy of ASP0113 in HCT, 7 subjects died and 2 additional subjects experienced CMV EOD out of 40 treated with ASP0113, for an overall failure rate of 22.5%, while among the placebo subjects 11 subjects died and 1 additional subject experienced CMV EOD out of 34, for an overall failure rate of 35.3%.<br>To detect the estimated difference in mortality and CMV EOD from the phase 2 study (35.3% vs. 22.5%), the study needs a sample size of at least 424 (212 per arm) to have 80% power at the 2-sided significance level of 0.050. A trial of size 500 (250 per arm) is expected to have 86% power for the composite endpoint.<br>The full analysis set (FAS) consists of all randomized subjects who receive at least 1 dose of randomized study drug.<br>Efficacy analyses will be limited to the data obtained from time first of dose of study drug through the 1 year post-transplant follow-up period. Data collected after the 1 year post-transplant follow-up period for the long-term follow-up will not be included for the primary analysis of efficacy variables.<br>Comparison between ASP0113 and placebo with respect to the primary endpoint will be performed using the Cochran-Mantel-Haenszel test stratified by factors for randomization (donor-recipient relatedness and donor CMV serostatus).<br>In a supportive analysis, time to first occurrence of a primary endpoint event will be analyzed in a Cox proportional hazards model where the time-to-event will be calculated in days from transplant to the earliest of death or CMV EOD. Subjects not experiencing the primary endpoint event will be censored at the time of their last follow-up visit. The Cox model will include treatment and the stratification factors for randomization.<br>Key secondary endpoints of time to first protocol defined CMV viremia

	through 1 year post-transplant and time to first adjudicated CMV-specific AVT through 1 year will each be compared between treatments using a Cox proportional hazards model with factors for treatment, donor-recipient relatedness, and donor CMV serostatus.<br>The endpoint of time to first CMV-specific AVT for protocol-defined CMV viremia or CMV EOD through 1 year post-transplant will be analyzed using a Cox proportional hazards model as described above.<br>Incidence of overall mortality, CMV EOD, grade 3-4 aGVHD, severe cGVHD, grade 3 infections other than CMV, relapse mortality, non-relapse mortality, graft rejection, failure to engraft, and relapse of primary disease requiring therapy will each be tested using a stratified analysis of the binary response using a CMH test to account for the stratification variables used as part of the randomization.<br>Number of episodes of protocol-defined CMV viremia will be compared between treatments using a stratum-adjusted Kruskal-Wallis test, adjusting for donor-recipient relatedness and donor CMV status.
	To estimate the association between CMV viremia and mortality, a multivariable Cox proportional hazards model will be constructed in a time-dependent manner. The model will be adjusted for the factors used to stratify the randomization. CMV viremia will be treated as a time-dependent variable, starting at the time of the first occurrence of protocol-defined CMV viremia.<br>The maximum grade of aGVHD, maximum cGVHD global score, and duration of hospitalizations will each be tested using a stratum-adjusted Kruskal-Wallis test.<br>The treatment effect on time to engraftment and time to platelet recovery will each be analyzed in a Cox proportional hazards model including the factors used to stratify the randomization.<br>Treatments will be compared with respect to each domain of the EQ-5D, the FACT-BMT, and productivity outcomes from the WPAI:GH using analysis of covariance (ANCOVA) of change from baseline to final visit with treatment, donor-recipient relatedness, and donor CMV serostatus as factors and baseline value as covariate.
**Safety Analysis**	The SAF will consist of all randomized subjects who have received at least 1 study drug injection. This analysis set will be used for all summaries of AEs, reactogenicity, clinical lab data, physical exams, vital signs and subject survival through 1 year post first study drug injection. All recorded AEs will be listed, including duration, outcome, toxicity grade, and association with use of study drug.
**Pharmaco-genomics (PGx)**	Subjects who consent to participate in the optional pharmacogenomics (PGx) sub-study will have a saliva sample collected during the baseline visit (V2), after randomization, but prior to the first dose of study drug. These samples will be stored for exploratory retrospective PGx analyses.

V. FLOW CHART AND SCHEDULE OF ASSESSMENTS

Flow Chart

Primary Study Period: Screening/ICF through 12-months post-transplant (Day 365/Visit 14)

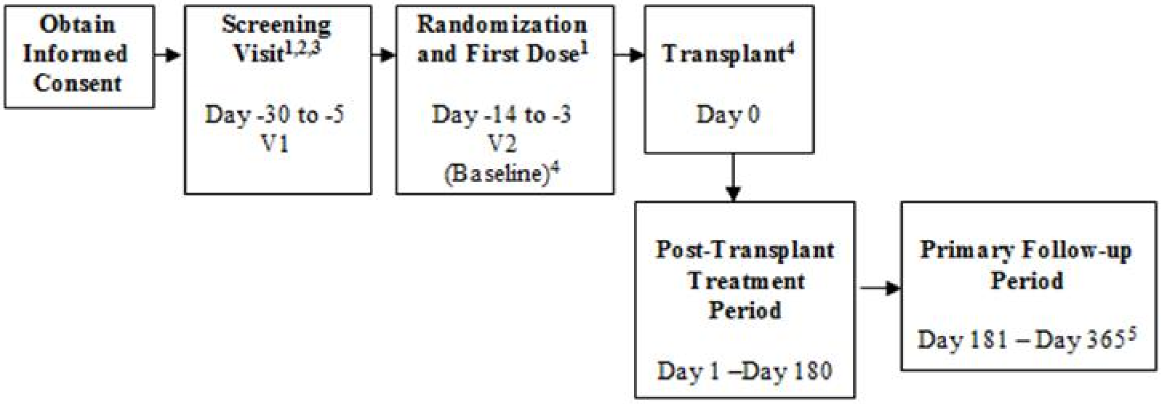

1. Screening visit and V2 (first dose) may occur either separately or on the same day. Eligibility will be determined using local laboratory results collected at the screening visit. The first dose of study drug must be administered prior to conditioning therapy. The dosing interval in relation to transplant/conditioning must be maintained.
2. Local laboratory assessments may be repeated 1 time if abnormal within the 25 day screening period.
3. The subject may be rescreened 1 time if the transplant is delayed and it will be outside the 25 day screening window. If outside the 25 day screening period, all screening procedures must be repeated.
4. Baseline is date of first dose (V2). The day of transplant (donor cell infusion) is day 0 and all visit days are relative to day 0.
5. Note that the day 365 visit must occur on day 365 +14 days (between day 365 to 379); it cannot occur prior to day 365.

Long-term Follow-up Period: Primary Study Period Completion through Long-term Follow-up Completion (Day 365/Visit 14 through 5.5 years Post-Transplant)

Long-term Follow-up Contact* 1.5 Years Post-Transplant	Long-term Follow-up Contact* 2 Years Post-Transplant	Long-term Follow-up Contact* 3 Years Post-Transplant	Long-term Follow-up Contact* 4 Years Post-Transplant	Long-term Follow-up Contact* 5 Years Post-Transplant	Long-term Follow-up Contact* 5.5 Years Post-Transplant
18 Months	24 Months	36 Month	48 Months	60 Months	66 Months

* Subjects will be contacted after the last injection of vaccine through 5.5 years post-transplant for long-term safety related to the DNA vaccine. Items to be assessed by telephone interview, and available patient records include; mortality, development of any new/recurrent cancer, development of infection requiring hospitalization or resulting in death, and erythema and induration at the injection site [Appendix 20].

Table 1 Schedule of Assessments – Primary Study Period Visit 1 (Screening) – Day 365

	**Screening**[2]	**Dose** [17] **(Baseline)**		**Transplant**[20]	**Dose 2**		**Dose 3**		**Dose 4**			**Dose 5**			**Primary StudyPeriod Completion / Early Termination Visit25**
Visit Number	V1[4]	V2[4]	V3[20]	V4	V5[24]	V6[24*]	V7	V8	V9	V10*		V11	V12*	V13	V14
Day Relative to Transplant or Prior Dose	-30 to -5	-14 to -3	Follow-up Dose 1 +10 to 14 days	0	14-40	Follow-up Dose 2 +10 to 14 days	60	Follow-up Dose 3 +10 to 14 days	90	Follow-up Dose 4 +10 to 14 days	100	180	Follow-up Dose 5 +10 to 14 days	270	365
Visit Window							±5 days		±10 days			±10 days		±10 days	+ 14 days (between Day 365 to 379)
Month	-1			0	1		2		3			6		9	12
Assessments															
Informed Consent [1]	X														
Subject Number Assignment[3]	X														
Inclusion/Exclusion Assessment	X	X^P													
Medical History & Demographics	X	X^P													
Clinical Evaluation (Physical Exam & Vitals)[5]	X	X^P			X^P		X^P		X^P			X^P		X	X
Concomitant Medication Review [6]	→	→	→	→	→	→	→	→	→	→	→	→	→		
Safety Laboratory[7]	X	X			X^P	X	X^P	X	X^P	X		X^P	X		
CMV Screening Serology[8]	X														
Modified HCT-CI Score[9]	X														
Pregnancy Test (females)[10]	X	X^P			X^P		X^P		X^P			X^P		X	X
Adverse Event and SAE Review[11]	→	→	→	→	→	→	→	→	→	→	→	→	→		
Randomization		X^P													
Health Economic Assessment (HEA)	→	→	→	→	→	→	→	→	→	→	→	→	→		
EQ-5D, FACT-BMT, WPAI [12]		X^P			X^P		X^P		X^P			X^P		X	X
Immunogenicity Laboratory[13]		X^P			X^P			X	X^P			X^P			X

Table continued on next page

	Screening[2]	Dose [17] (Baseline)		Transplant[20]	Dose 2		Dose 3		Dose 4			Dose 5			Primary StudyPeriod Completion / Early Termination Visit25
Visit Number	V1[4]	V2[4]	V3[20]	V4	V5[24]	V6[24*]	V7	V8	V9	V10*		V11	V12*	V13	V14
Day Relative to Transplant or Prior Dose	-30 to -5	-14 to -3	Follow-up Dose 1 +10 to 14 days	0	14-40	Follow-up Dose 2 +10 to 14 days	60	Follow-up Dose 3 +10 to 14 days	90	Follow-up Dose 4 +10 to 14 days	100	180	Follow-up Dose 5 +10 to 14 days	270	365
Visit Window							±5 days		±10 days			±10 days		±10 days	+ 14 days (between Day 365 to 379)
Month	-1			0	1		2		3			6		9	12
Platelet count[14]		X^P			X^P		X^P		X^P			X^P			
Pharmacogenomics (PGx) – (Optional)[15]		X^P													
CMV Plasma Viral Load Testing[16]				Start Weekly Testing; See Table 1a							Every Other Week		Every 30 days		
**Study Drug Injection**[17]		X[17]			X		X		X			X			
Local Reactogenicity Assessment[18, 19]		X			X		X		X			X			
Transplant Information[21]				X											
DONOR Blood Sample (Optional)[22]				X											
Karnofsky Performance Scale		X			X		X		X			X		X	X
aGVHD and cGVHD[23] (See Table 1a)					X		X		X			X		X	X

P = procedure to be completed prior to dosing *= Visit procedures may be performed by Home Health Care (HHC), if applicable in the country. Note: When CMV Plasma Viral Loads fall outside of a regular clinic Visit, the blood draw may be performed by HHC.

Table 1a CMV Plasma Viral Load Testing (Central Laboratory) and GVHD Assessments

Assessment Number	V4 Transplant	A1	A2	A3	A4	A5	A6	A7	A8	A9	A10	A11	A12	A13	A14	A15	A16	A17	A18	A19	A20	A21	A22	A23	A24	A25
	Day 0	Day 7 Week 1	Day 14 Week 2	Day 21 Week 3	Day 28 Week 4	Day 35 Week 5	Day 42 Week 6	Day 49 Week 7	Day 56 Week 8	Day 63 Week 9	Day 70 Week 10	Day 77 Week 11	Day 84 Week 12	Day 91 Week 13	Day 98 Week 14	Day 112 Week 16	Day 126 Week 18	Day 140 Week 20	Day 154 Week 22	Day 168 Week 24	Day 198	Day 228	Day 258	Day 288	Day 318	Day 348
Visit Window (days)		±4	±2	±2	±2	±2	±2	±2	±2	±2	±2	±2	±2	±2	±2	±5	±5	±5	±5	±5	±5	±5	±5	±5	±5	±5
CMV[16]		X	X	X	X	X	X	X	X	X	X	X	X	X	X	X	X	X	X	X	X	X	X	X	X	X
aGVHD[23]		X	X	X	X	X	X	X	X	X	X	X	X	X	X											

Note: When CMV Plasma Viral Loads fall outside of a regular clinic visit, the blood draw may be performed by HHC.

1. Informed consent must be obtained prior to the performance of any study-related procedure and before randomization.
2. Screening procedures may be completed -30 to -5 days prior to the day of transplant. Local laboratory results are to be used for screening purposes. The screening laboratory results may be repeated 1 time throughout the noted period. The subject may be rescreened 1 time if the transplant is delayed and it will be outside the 25 day screening window. If outside the 25 day screening period, all screening procedures must be repeated.
3. Subjects will be assigned a subject number for use throughout the study at the screening visit via the Interactive Response Technology (IRT) system.
4. Screening visit and V2 (first dose of study drug) may occur either separately or on the same day.
5. A complete physical examination (PE) will be conducted at the screening (V1) and at the primary study period completion/day 365 visit (V14). At all other visits where a PE is performed, symptom-directed PEs may be done. A detailed description of the subject's current disease status and conditioning regimen (including doses) will be recorded at the baseline visit (V2). The evaluations conducted at each visit are to be performed by the Investigator or qualified medical personnel who routinely perform these evaluations in this subject population. Vital signs will be collected immediately prior to injection and 60 (±10) minutes post injection (except weight) at study visits accompanied by injection and once at screening (V1), and at V13 & 14. Vital signs include blood pressure, pulse rate, respiratory rate, temperature and weight. Height will be collected at the screening visit. Performance status will be assessed using the Karnofsky Performance Scale (KPS) at baseline visit (V2).
6. All concomitant medications and therapies administered from 30 days prior to transplant through 30 days post last dose of study drug will be collected on the electronic case report form; Concomitant medications and therapies associated with Grade 3 and higher grade adverse events (AEs) as well as all serious adverse events (SAEs) will be collected from 31 days post last dose of drug through 1 year post-transplant. Over-the-counter medications, and herbal remedies do not need to be captured beginning 30 days after the last dose of study drug.

Footnotes continued on next page

7. Safety Laboratory will be drawn at the screening visit using the local laboratory. Safety laboratory will be done by a central laboratory at baseline, before doses 2-5, and at each follow-up time point for doses 2-5, and will include hematology and biochemistry, including a hepatic profile. Please refer to [Protocol Appendix 1] for a full list of routine safety parameters that will be tested, and to [Section 5.4.3] and the Laboratory Manual for further instruction on laboratory collection and processing.
8. Screening for cytomegalovirus (CMV) immunoglobulin G (IgG) seropositivity by the local laboratory may be performed up to 30 days prior to transplant and before randomization.
9. The modified hematopoietic cell transplant-comorbidity index (HCT-CI) score are to be completed during screening with history and laboratory from the screening visit. Local laboratory results are to be used in the assessment. Local laboratories may be repeated once to score the HCT-CI. Pulmonary function tests (PFTs) including forced expiratory volume (FEV1) and diffusing lung capacity of carbon monoxide (DLco), multi gated acquisition scan (MUGA), and/or echocardiograms performed within 6 months (180 days) prior to screening are to be used for scoring pulmonary and cardiac comorbidities. PFTs (FEV1 and DLco), MUGAs /or echocardiograms not performed within 6 months of screening will have to be repeated for scoring purposes.
10. For all females of childbearing potential and all females who are of non-childbearing potential but are < 50 years of age and not documented to be surgically sterile, a urine or serum pregnancy test will be performed at screening, on the same day and prior to each dose, at visit 13, and at the primary study period completion/day 365 visit (V14). All pregnancy tests will be done locally. For subjects who receive mycophenolate mofetil, additional pregnancy testing should be done in accordance with local regulatory requirements.
11. All AEs and SAEs will be collected from signing of the informed consent through 30 days post last dose of study drug. All events requiring adjudication (CMV end organ disease [EOD], initiation of CMV-specific antiviral therapy [AVT], and cause of death), grade 3 and higher grade AEs as well as all SAEs will be collected from 31 days post last dose of drug through 1 year post-transplant. A change in medical status or medical history from time of signing of informed consent through first dose of study drug is to be reported as an AE or SAE as applicable. In this study, the day 0 HCT is not considered an AE.
12. The EuroQol – 5D (EQ-5D), Functional Assessment of Cancer Therapy – Bone Marrow Transplant (FACT-BMT), and Work Productivity and Activity Impairment Questionnaire (WPAI) are to be completed by the subject prior to any other study assessments or visit procedures. Subjects are to complete these assessments in the following order: EQ-5D first, FACT-BMT second, WPAI:GH last. For all 3 patient-reported outcome measures, i.e. EQ-5D, FACT-BMT, WPAI, the answers must come from the subject. If the subject is unable to fill out the questionnaire, the assessment should not be performed. If the subject is unable to answer a question, the rest of the questionnaire still needs to be completed.
13. Immunogenicity laboratories include collection of blood for the glycoprotein B (gB) antibody and phosphoprotein 65 (pp65) T-cell assays. Samples are to be drawn on visits V2, V5, V8, V9, V11 and V14. Samples for T-cell assays will not be collected if the absolute lymphocyte count is known to be < 500 mm^3 by local or central laboratory measurement. If the visit coincides with a study drug administration, the sample will be drawn prior to injection.
14. The platelet count must be at least ≥ 50000 mm^3 (spontaneously or after platelet transfusion) performed by the local laboratory within 3 days prior to all study drug injections. Prior to all study drug injections, confirmation must be made that there is no medical contraindication to an IM injection. Results received in an equivalent local unit of measure must be converted to SI units. If the subject's platelet count does not meet this threshold at the time of the visit, the subject may return for the study drug injection and local reactogenicity assessment at a later time within the allowable window for the visit. If the subject's platelet count does not meet this threshold during the visit window, the dose must be skipped and the corresponding follow-up safety laboratory and reactogenicity assessment should not be done. The second injection should be given as close to, but not prior to, day 14 as possible.
15. Subjects who consent to participate in the pharmacogenomics (PGx) substudy will have a saliva sample collected during the baseline visit (V2), after randomization but prior to the first dose of study drug.
16. Plasma viral load by the central laboratory will be monitored weekly (± 2 days) during days 0-100, every other week (±5 days) during days 101-180, every 30 days (±5 days) during days 181-365, at the initiation of CMV-specific AVT, when clinically indicated, and every time a viral load sample is sent to the local laboratory. When CMV-specific AVT is initiated, a central CMV viral load will be obtained a minimum of weekly until CMV-specific AVT is discontinued. Thereafter, the regularly scheduled viral load assessments will resume according to the scheduled protocol assessments.

Footnotes continued on next page

17. The first dose is to be given at day -14 to -3 prior to transplant and within 72 hours prior to the start of chemotherapy and radiation therapy for conditioning.
18. The subject must be monitored directly for the first 15 minutes of the injection. The subject must be in the clinic, but does not require direct monitoring, from 15 minutes through 1 hour after the injection.
19. Local reactogenicity will be evaluated 1 hour (± 10 minutes) after each injection and for each of the 7 consecutive days following each injection beginning approximately 24 hours following each dose (day 1-7 postdose). The assessments for each of the 7 consecutive days following an injection will be done by the subject and reported to the site via diary. When reactogenicity is reported as an AE, it is to be followed until resolution or medically stable. All local reactions ≥ grade 3 (Appendix 7) require confirmation by a health care professional.
20. The date of transplant (donor cell infusion) defines day 0 and all visit days are relative to day 0. Based on this schedule, V3 may occur prior to or after the day of transplant, day 0.
21. Transplant information includes recipient transplant volume infused, HLA typing of the donor and recipient, HLA cross match at time of transplant (as determined by the site's standard method of determination and, if available), type of transplant, the number of CD34+ stem cells/kg infused if available, donor and recipient CMV serostatus, if available the hepatitis B virus (HBV) serostatus, hepatitis C virus (HCV) serostatus, and Epstein Barr Virus (EBV) serostatus of the donor and recipient; blood type of the donor and recipients. ABO cross match (if available), donor-recipient relatedness, donor gender, age, race (if available) and ethnicity (for the USA only and if available); donor product plasma, volume and RBC depletion status; and if available donor cell product CD3+, CD4+, CD8+ and CD56+ cell counts. For bone marrow recipients, the number of mononuclear cells transfused must also be included, if possible.
22. *Optional*: A 20 mL peripheral blood sample from the CMV seropositive donors will be obtained prior to the stem cell donation for recipients participating in the main trial to test for CMV-specific T-cells (requires donors' consent). For those donating peripheral stem cells, the donor blood sample is to be collected prior to the initiation of mobilization therapy (e.g., G-CSF). Not applicable to sites in Japan.
23. Acute and chronic GVHD is to be assessed and graded/globally scored and the KPS will be used to assess performance status at baseline and V5, V7, V9, V11, and V13 following transplant (day 0) through the primary study period completion/day 365 visit (V14) except when the visit is conducted by HHC. In addition, aGVHD needs to be assessed weekly through day 100. When a diagnosis of aGVHD is made for a subject, assessments are to continue weekly until resolution for maximal grade and stage by key organs. Local laboratories may be used for staging. When a diagnosis of cGVHD or aGVHD is made by a health care professional for a subject, the subject should return to the study center for evaluation and data collection, and assessments are to be done at each regularly scheduled protocol visit until resolution; the maximum score during the interval from the last assessment to the current assessment is to be recorded for assessments performed at the study site.
24. If the subject does not meet the criteria for dosing by day 40, V5 procedures (except study drug injection and local reactogenicity assessment) need to be completed within 5 days of day 40 (day 40 to 45) and V6 should be skipped.
25. The day 365 visit must occur on day 365 +14 days (between day 365 to 379); it cannot occur prior to day 365 for subjects who do not prematurely withdraw from both treatment and continued follow-up. For a subject who prematurely withdraws from the study (discontinues treatment with no continued follow-up), the study completion visit is to be completed within 14 days of study withdrawal.

Table 2 Schedule of Assessments – Long-term Follow-up Period

Month (post-transplant)	18	24	36	48	60	66
**Year (relative to transplant/Day 0)**	1.5	2	3	4	5	5.5
**Window (months)**	± 1	± 1.5	± 1.5	± 1.5	± 1.5	±1.5
Mortality, including primary cause of death and date	X	X	X	X	X	X
Development of any new/recurrent cancer	X	X	X	X	X	X
Development of infection requiring hospitalization or resulting in death	X	X	X	X	X	X
Erythema or induration at sites of study drug injections	X	X	X	X	X	X

Subjects will be contacted after the primary study period (day 365) through 5.5 years post-transplant for long-term safety related to the DNA vaccine. Items to be assessed by telephone interview, and available patient records include; mortality, development of any new/recurrent cancer, development of infection requiring hospitalization or resulting in death, and erythema or induration at sites of study drug injection (see [Appendix 20]). During the long-term follow-up period, if an SAE is identified and deemed possibly or probably related to the study medication, an SAE report on the SAE worksheet must be sent to the Sponsor per [Section 5.5.6], for all sites except those in Japan. For sites in Japan only: an SAE report on the JUTOKUNA YUUGAIJISHOU HOUKOKUSHO must be sent to the delegated CRO per [Section 5.5.6].

1 INTRODUCTION

Cytomegalovirus (CMV) is a member of the Herpesviridae family that establishes latency in host cells after acute infection. Immunocompromised patients, including patients undergoing hematopoietic cell transplant (HCT), are particularly vulnerable to CMV infection and reactivation. CMV infection is a risk factor for CMV end organ disease (EOD), as well as a risk factor for acute graft-versus-host disease (aGVHD), chronic graft-versus-host disease (cGVHD), bacterial and fungal infections, and increased mortality. ASP0113 is a DNA vaccine designed to prevent CMV infection in immunocompromised hosts. It contains 2 plasmids which encode the antigens gB and pp65 to induce humoral and cellular immunity to the virus. A phase 1 and phase 2 study suggest that the vaccine is safe, immunogenic and effective in preventing CMV viremia in CMV seropositive subjects undergoing hematopoietic transplant. This phase 3 pivotal, randomized, double-blind, placebo-controlled trial is designed to assess the incidence of mortality through 1 year post-transplant in the ASP0113 group as compared to the placebo group.

1.1 Background

Members of the Herpesviridae family are a group of viruses that includes the CMV, Epstein-Barr virus (EBV), herpes simplex viruses and varicella zoster virus. Herpesviridae viruses are double-stranded DNA viruses that become latent in host cells after acute infection. CMV infection is ubiquitous and evidence of seroconversion has been found in all populations. CMV infection is usually harmless, except in the fetus and immunocompromised hosts. In the immunocompromised host, primary infection, re-infection, and reactivation can cause significant morbidity and mortality.

Persons undergoing HCT are particularly susceptible to both primary infection and reactivation of CMV as a result of the interventions and drugs that ablate and suppress their immune systems. Patients with viremia that results from primary infection or reactivation of CMV are at greatly increased risk of CMV tissue-invasive disease and may also be at risk for mortality not resulting from direct tissue invasion of CMV, aGVHD, cGVHD and non-CMV infections due to the immunomodulatory effects of CMV [Cantoni et al, 2010; Boeckh & Ljungman, 2009].

CMV tissue-invasive disease was the leading cause of death due to infectious disease among CMV-seropositive recipients of HCT prior to the introduction of effective antiviral therapy (AVT) in the 1990s. Since the introduction of effective AVT, the mortality from CMV disease during the first 3 months after HCT has been reduced from 20-30% to less than 5% in most studies [Boeckh and Nichols, 2004]. However, despite the significant decrease in CMV tissue-invasive disease related mortality, many studies indicate that survival continues to be decreased in CMV-seropositive patients, suggesting an indirect effect of subclinical CMV infection on mortality [Craddock et al, 2011; Yakoub-Agha et al, 2006; Boeckh & Nichols, 2004]. Mortality related to CMV seropositivity may partially result from an increase in aGVHD, cGVHD and infections other than CMV as these outcomes result in decreased survival and are associated with CMV infection and CMV seropositivity in the recipient or donor [Srinivasin et al, 2012; Cantoni et al, 2010; Larsson et al, 2004; Nichols et al, 2002].

Two strategies currently exist to prevent CMV disease in transplant recipients. In the preemptive approach, viremia is monitored on a regular schedule and when a certain threshold of viremia is detected, AVT is initiated. Alternatively, in the prophylactic approach, AVT is administered for an arbitrary period of time to prevent reactivation. In HCT, preemptive therapy is used more commonly than prophylaxis because of the toxicities associated with CMV-specific AVT, including myelosuppression.

Given the side effect profile of current AVT used to prevent CMV disease, and the inability of current strategies to prevent CMV infection, alternate strategies to prevent CMV disease and infection are needed. ASP0113 is a first-in-class therapeutic vaccine that is designed for the prevention of CMV infection in HCT recipients through the stimulation of both humoral and cell-mediated immune responses. It contains 2 plasmids which encode CMV glycoprotein B (gB) and tegument phosphoprotein 65 (pp65). gB is the major neutralizing target for antibodies stimulated by CMV in healthy humans [Britt et al, 1990]; pp65 is 1 of the proteins most frequently recognized by $CD8^+$ and $CD4^+$ cells in healthy humans [Kern et al, 2002; Gyulai et al, 2000; Wills et al, 1996]. ASP0113 appears to be both effective and safe and therefore warrants further study.

1.2 Product Composition

Plasmid DNA (pDNA) is covalently closed, circular, double-stranded DNA molecules that are engineered to encode protective antigens from infectious disease pathogens. This platform technology provides an ideal approach for treating immunocompromised subjects because it eliminates the safety concerns engendered by the use of live viral vectors yet preserves the ability to induce both cellular and humoral responses. In addition, a pDNA vaccine is delivered without other viral genes that may be involved in evasion of the immune system; ASP0113 does not include CMV-encoded genes involved in the downregulation of major histo-compatibility complex (MHC) molecules, production of chemokine receptors, or mimics of cytokines [Mocarski, 2002].

ASP0113 is a bivalent product that consists of 2 separate pDNAs each containing a codon-optimized CMV gene. Phosphoprotein 65 (pp65) is included to induce CD4+ and CD8+ T-cell responses; gB is included to induce antibodies. The plasmids are formulated with CRL1005 poloxamer and BAK, a cationic surfactant, in PBS. The combination of CRL1005 and BAK produces a thermodynamically stable self-assembled particulate system at room temperature and is used as a gene delivery system. The addition of CRL1005 and BAK has been shown to enhance immunogenicity in mice vaccinated with ASP0113.

1.3 Nonclinical and Clinical Data

1.3.1 Nonclinical Data

Vaccination of mice with ASP0113 led to production of antibodies against gB and, to a lesser extent, against pp65. Vaccination of mice with A0113 did result in a robust T-cell response to pp65. Plasmid copy number of ASP0113 was not detectable or quantifiable in the gonads, spleen, liver, heart, lymph node, kidney, brain or blood 15 days post injection. On day 62 post injection, plasmid was present at the bone marrow and the injection site, however,

their copy numbers were reduced by approximately 596-fold and 76-fold, respectively, compared to day 3. No genomic integration of the pDNA was observed 2 months after vaccination.

Administration of ASP0113 as a single dose is well-tolerated and results in no demonstrable clinical signs of toxicity due to injection of vaccine. Repeat dose administration of ASP0113 in rabbits is well-tolerated, with no drug-related changes in clinical signs. However, vaccination with ASP0113 did lead to a significant, but reversible, increase in creatine phosphokinase (CPK) and minimal to moderate inflammation of the muscle, skin and subcutis, that persisted to a slight degree in the recovery period. Based on these nonclinical animal studies, ASP0113 is considered safe and well-tolerated, is not anticipated to present a toxicity risk in humans when delivered by IM injection, and immunogenicity data suggest that vaccination can induce immune responses to human CMV. For a detailed summary of the nonclinical studies please refer to the Investigator's Brochure (IB).

1.3.2 Clinical Data

To date, ASP0113 has been evaluated in a phase 1 study in healthy volunteers and a phase 2 study of matched related donor/recipient pairs and related or unrelated recipients undergoing allogeneic HCT for hematologic disorders.

1.3.2.1 Phase 1 (VCL-CB01-101) Results

The first clinical study of ASP0113 was a multicenter, randomized, open-label phase 1 clinical study (VCL-CB01-101) designed to evaluate the safety and immunogenicity of ASP0113 in 44 healthy adult CMV-seropositive and CMV-seronegative volunteers at doses of either 1 mg or 5 mg IM administered on weeks 0, 2, and 8 in a dose escalation format and at a dose of 5 mg IM administered on days 0, 3, 7 and 28 (accelerated schedule)[Wloch et al, 2008].

Overall ASP0113 was safe and well-tolerated. Volunteers receiving the higher dose or accelerated vaccine schedule tended to experience more local reactogenicity, but the severity of the treatment-emergent adverse events (TEAEs) did not increase with increasing dose and a more compressed schedule. The incidence of local reactogenicity, predominantly injection site pain, was 62.5% for the lowest weekly dose group, 87.5% for the highest weekly dose group, and 100% for the accelerated schedule group. Several cutaneous TEAEs were considered related to ASP0113 by the Investigator and included erythema, rash and pruritus. One volunteer did experience a transient increase in CPK value greater than 5 x the upper limit of normal (ULN), but less than 10 x normal.

Immune responses to the 2 immunogens encoded in the vaccine, gB and pp65, were assessed by the use of an indirect gB binding enzyme-linked immunosorbent assay for antibody responses to gB and a pp65 direct ex-vivo interferon γ (IFN-γ) enzyme-linked immunosorbent spot assay for T-cell responses to pp65. At 16 weeks, antibody and/or cell responses were elicited in 38% and 50% of the CMV-seronegative volunteers administered 1 mg and 5 mg ASP0113, respectively. CMV-seropositive volunteers had increases in pp65 T-cell responses of 12.5% and 37.5% across all groups, but no increase in gB antibody levels were detected for CMV-seropositive volunteers.

1.3.2.2 Phase 2 (CB01-202) Results

The second clinical study of ASP0113 was a multicenter, randomized, double-blind phase 2 clinical study designed to evaluate the efficacy, immunogenicity and safety of ASP0113 in donors and CMV-seropositive recipients undergoing 5 of 6 or 6 of 6 HLA-allele-matched allogeneic HCT for the treatment of hematologic disorders [Kharfan-Dabaja, 2012]. A total of 108 subjects were enrolled in the study, including 14 donor/recipient pairs, and received at least 1 dose of study drug [5 mg/mL (1 mL dose)] or placebo. Recruitment of donor/ recipient pairs was found to be impractical early in the study so that arm was discontinued; the results are not discussed here. The intent-to-treat (ITT) and per protocol (PP) populations comprise 80 and 74 transplant recipients, respectively. Recipients received injections at approximately days -5 to -3, 21 to 42 (1 injection in this window, health permitting), on day 84, and on day 196 relative to transplantation. Results shown below are for the PP population.

The primary efficacy endpoint of the study was the occurrence of clinically significant CMV viremia, defined as detection of CMV viremia that resulted in the initiation of CMV-specific antiviral therapy during the first year of transplant. The clinical decision to treat at the sites was based on the most current local or central CMV assay results. CMV-specific antiviral therapy was initiated for a lower percentage of recipient subjects in the ASP0113 group (47%) than in the placebo group (61.8%), but the difference was not statistically significant (P = 0.145, Cochran-Mantel-Haenszel [CMH] test stratified by site). The PP population was modified for this analysis to exclude 2 subjects who received CMV-specific AVT because of false positive results from the local viral load assay).

When the occurrence of CMV viremia, as defined as ≥ 500 copies/mL by a laboratory developed test (LDT) based upon the LightCycler® polymerase chain reaction (PCR), was evaluated in a central laboratory, the rate was lower in the ASP0113 group (33%) than in the placebo group (62%) and was statistically significant (P = 0.008, CMH test stratified by site).

Post-hoc analyses of the phase 2 data also suggest that the clinical endpoints which have been reported to be associated with CMV infection may be reduced as a result of vaccination with ASP0113, including mortality, aGVHD and cGVHD. For the clinical endpoints of aGVHD, cGVHD, and mortality and a composite of CMV EOD, aGVHD, cGVHD and mortality, the observed rate for ASP0113 was lower than the corresponding observed rate for placebo, although the differences were not statistically significant [Table 3].

Table 3 Incidence of CMV EOD, Death, aGVHD (Grade 3-4), cGVHD (High Risk) and a Composite of All Variables

	ASP0113 (n=40)	Placebo (n=34)	P value
**CMV EOD**	3 (7.5%)	3 (8.8%)	1.000§
**Mortality**	7 (17.5%)	11 (32.4%)	0.1775§
**aGVHD (grade 3-4) †**	2 (5.0%)	5 (14.7%)	0.2364§
**cGVHD (high risk) ‡**	4 (10.0%)	7 (20.6%)	0.3259§
**Composite**	13 (32.5%)	18(52.9%)	0.0995§

aGVHD: acute graft-versus-host disease; cGVHD: chronic GVHD; CMV: cytomegalovirus; EOD: end-organ disease

Per-protocol population: all unpaired recipients who met the inclusion/exclusion criteria and received at least 1 injection and had at least 1 post-transplant assessment relevant to an efficacy endpoint.

†Glucksberg et al, Transplantation 1974

‡Akpek et al, Blood 2003

§ Fisher's Exact Test

Further investigation of the phase 2 study results of the relationship between CMV viremia and potential phase 3 endpoints was investigated by Cox proportional hazards models using past or current viremia as a time-dependent covariate. Results of these analyses are summarized in [Table 4]. Although incidence of each of these events is numerically greater among subjects who have had CMV viremia, the effect of CMV viremia is not statistically significant.

Table 4 Results of Cox Proportional Hazards Models on the Effect of Past or Current CMV Viremia (defined as viral load > 1000 by central lab assay) on Endpoint Events

Component	Events	Hazard Ratio	95% CI	P value
aGVHD (Grade 3-4)	7	1.13	0.20, 6.51	0.88
cGVHD (Severe)	11	1.27	0.39, 4.16	0.69
Severe Infection (CTCAE Grade 3-5)	48	1.72	0.84, 3.49	0.14

aGVHD: acute graft versus host disease; cGVHD: chronic graft versus host disease; CTCAE: common terminology criteria for adverse events; CMV: cytomegalovirus

Immune responses to the 2 immunogens, gB and pp65, encoded in the vaccine were assessed by 2 immunoassays: 1) a gB-specific IgG-binding ELISA; and 2) an ex vivo pp65-specific IFN-γ ELISPOT [Kharfan-Dabaja, 2012]. T-cell responses to CMV pp65 and gB as well as gB-specific serum antibody levels were similar in the ASP0113 and placebo recipients prior to conditioning therapy and transplant. The CMV pp65 T-cell responses were numerically enhanced in the ASP0113 group relative to the placebo group at all post-transplant times evaluated; the differences in the responses were statistically significant by the Wilcoxon rank sum test at day 84 (P = 0.036). Please refer to the IB regarding other post-hoc analyses of the immunogenicity data.

The geometric mean gB-antibody levels in recipients in the ASP0113 group compared with recipients in the placebo group showed a trend towards significance at the fourth injection (P = 0.064, Wilcoxon rank sum test) and reached significance by day 365 (P = 0.009, Wilcoxon rank sum test). Although the geometric mean gB antibody levels were higher for the ASP0113 recipients at all time points evaluated, the repeated measurement ANOVA using $\log_{10}$ transformed data from days 56 to 365 did not show a significant difference between the longitudinal gB antibody responses (P = 0.749).

Overall the vaccine appears to be safe and well-tolerated. The incidence of local reactogenicity was higher in the ASP0113 group than in the placebo group (22.9% and 10.9%, respectively), primarily due to injection site pain. Fatalities were reported for 20.8% recipients in the ASP0113 group and 32.6% recipients in the placebo group during the 1-year study follow-up. No recipient died due to study drug-related AEs. No subject experienced CPK elevation in this study. One subject in the ASP0113 arm discontinued the study due to a serious allergic reaction that was considered by the Investigator to be possibly related to ASP0113. The subject developed clinical signs of swollen mouth and tongue without respiratory distress 50 minutes after the second vaccination. The subject received hydrocortisone, diphenhydramine and ranitidine and fully recovered.

1.4 Summary of Key Safety Information for Study Drugs

ASP0113 in nonclinical studies was in general well-tolerated. Nonclinical studies demonstrated that the risk of integration of injected DNA into the host's genome is negligible per FDA regulatory guidance and induction of autoimmunity is unlikely. No systemic organ toxicity was noted. There was no local reactogenicity reported, but in the highest dose groups in a repeated-dose toxicity study in rabbits, there was histologic evidence of inflammation in the muscles injected with vaccine and concurrent elevations in CPK.

In the phase 1 and phase 2 clinical studies the vaccine was safe and well-tolerated. One healthy volunteer had a transient elevation in CPK (> 5x normal but < 10x normal). Local (injection site) reactions including pain, induration, erythema, pruritus, discomfort and swelling were reported more frequently in the higher dosing group and more accelerated schedule with ASP0113 in healthy volunteers and in the ASP0113 treated group when compared to the placebo group. The only discontinuation due to an SAE that was possibly related to ASP0113 was the transplant recipient who was previously discussed in [Section 1.3.2.2].

1.5 Risk-Benefit Assessment

Despite currently licensed CMV-specific antiviral therapies, CMV disease continues to cause significant morbidity and mortality in transplant recipients and CMV infection may be associated with multiple other clinical outcomes including infections other than CMV aGVHD, cGVHD and mortality. In addition, the treatments and associated toxicities can incur significant healthcare costs.

ASP0113 appeared to show efficacy in a single phase 2 trial over multiple secondary end-points. The incidence of local reactogenicity may be greater with ASP0113 than placebo. In addition, while there were CPK elevations at the highest dose level in toxicity studies in

rabbits, significant damage to the muscle, as determined by CPK elevations greater than 10 times the ULN, has not been observed in humans. One SAE (hypersensitivity/allergic reaction) leading to discontinuation responded well to medical treatment and was deemed not life-threatening. False positive results in the assessment of CMV viral load can occur in subjects receiving ASP0113 with the use of PCR assays that utilize primers targeted at gB and pp65. Such false positive results, if unrecognized, can lead to misdiagnosis and unnecessary initiation of AVT.

Given the continued morbidity and mortality associated with CMV infection and treatment, the evidence of efficacy in the phase 2 trial in HCT recipients and the safety profile of ASP0113, continued investigation is warranted.

2 STUDY OBJECTIVES, DESIGN AND VARIABLES

2.1 Study Objectives

- To evaluate the efficacy of ASP0113 compared with placebo as measured by a primary composite endpoint of overall mortality and CMV EOD through 1 year post-transplant.
- To evaluate the safety of ASP0113 in subjects undergoing allogeneic HCT.

2.2 Study Design and Dose Rationale

2.2.1 Study Design

This is a randomized, double-blind, placebo-controlled trial of approximately 500 subjects being conducted in North America, Europe, Asia, and Australia. Subjects will be randomized in a 1:1 ratio, ASP0113 or placebo, and stratified by donor-recipient relatedness and donor CMV serostatus. At least 30% of subjects enrolled will be donor-seronegative. The test compound will be a vaccine (ASP0113) which contains 2 plasmids encoding gB and pp65, each at 2.5 mg/mL, formulated with CRL1005 poloxamer and benzalkonium chloride (BAK). The placebo control will be phosphate-buffered saline (PBS). Subjects will receive either 5 doses of ASP0113 or placebo on days -14 to -3, 14-40, 60 ±5, 90 ±10 and 180 ±10 in relation to the day of transplant (donor cell infusion). The first injection must be given within 72 hours prior to the start of chemotherapy and radiation therapy for conditioning and the second injection should be given as close as possible to, but not prior to, day 14. The platelet count must be at least ≥ 50000/mm^3 (spontaneously or after platelet transfusion) performed by the local laboratory within 3 days prior to all study drug injections. Prior to all study drug injections, confirmation must be made that there is no medical contraindication to an IM injection. Results received in an equivalent local unit of measure must be converted to SI units. Syringes will be masked prior to dosing to blind the subjects and all other personnel who need to remain blinded to the treatment assignment (i.e., site staff other than pharmacy personnel and staff designated to administer study drug injections).

The pre-specified primary endpoint is the composite of overall mortality and CMV EOD, through 1 year post-transplant. Despite the significant decrease in CMV tissue-invasive disease related mortality, many studies indicate that survival continues to be decreased in CMV-seropositive patients, suggesting an indirect effect of subclinical CMV infection on

mortality (see Table 5) [Craddock et al, 2011 Yakoub-Agha et al, 2006; Boeckh & Nichols, 2004].

Table 5 Association of CMV with Patient Survival

Source	Number of Patients	Underlying Disease	CMV-seropositive Recipients Compared with CMV-seronegative Recipients with a Seronegative Donor
Broers et al, 2000	115	Mixed	24% absolute decline in OS (P = 0.01)
McGlave et al, 2000	1423	CML	20% relative decline in DFS (P = 0.002)
Cornelissen et al, 2001	127	ALL	38% relative decline in DFS (P = 0.05)
Craddock et al, 2001	106	CML	22% absolute decline in OS (P = 0.006)
Kroger et al, 2001	125	Mixed	41% absolute decline in OS (P < 0.001)
Castro-Malaspina et al, 2002	510	MDS	46% relative decline in DFS (P = 0.001)
Doney, 2003	182	ALL	99% relative rise in TRM (P =.01)
Yakoub-Agha et al, 2006	236	Mixed	16.4% absolute decline in OS (P = 0.01)
Craddock et al, 2011	168	Primary refractory AML	13% absolute decline in OS (P = 0.09)

Table modified from Boeckh & Nichols, 2004.

AML: acute myelogenous leukemia; CML: chronic myelogenous leukemia; CMV: cytomegalovirus; DFS: disease free survival; MDS: myelodyplastic syndrome; OS: overall survival; TRM: transplant-related mortality

Some publications have suggested that mortality related to CMV seropositivity may partially result from an increase in aGVHD, cGVHD and infections other than CMV as these outcomes result in decreased survival and are associated with CMV infection and CMV seropositivity in the recipient or donor [Srinivasin et al, 2012; Cantoni et al, 2010; Larsson et al, 2004; Nichols et al, 2002].

A key secondary endpoint is CMV viremia. CMV viremia will be protocol-defined as CMV plasma viral load of ≥ 1000 IU/mL as assessed by the central laboratory through 1 year post-transplant. Another key secondary endpoint is adjudicated CMV-specific AVT through 1 year post-transplant.

CMV plasma viral load will be monitored per the following schedule during the primary study period (through day 365/V14): weekly (± 2 days) during days 0-100, every other week (± 5 days) during days 101-180, every 30 days (± 5 days) during days 181-365. The CMV plasma viral loads will be performed by the central laboratory. CMV plasma viral load will also be performed by the central laboratory at the initiation of CMV-specific AVT. Upon initiation of CMV-specific AVT, plasma viral load testing will be performed at least weekly until the CMV-specific AVT is discontinued, at which point, the regularly scheduled plasma viral load assessments will resume.

Note: CMV plasma viral loads may be performed at a local laboratory approved by the Sponsor and at the discretion of the Investigator. However, every time a viral load sample is sent to the local laboratory a sample will also be sent to the central laboratory for confirmatory testing. Pre-emptive therapy may be started based on the central or local laboratory assessment and the subject's clinical condition.

As 1 of the plasmids of the ASP0113 vaccine codes for the pp65 antigen, the theoretical possibility of interference with the pp65 antigenemia assay has been raised. At present, there are no data to suggest that such interference occurs. However, to ensure patient safety and study integrity, sites that use pp65 antigen as their local methodology for the detection of CMV infection must adhere to the procedures defined below as a condition of participation in the trial.

- The local pp65 antigenemia results must be entered into the eCRF database within 72 hours (or next business day) of receipt to facilitate in-time monitoring of results.
- The Medical Monitor must be called within 48 hours (or next business day) if the pp65 antigenemia test is positive and the central laboratory PCR viral load result is negative.
- Both the pp65 antigenemia and the central laboratory test must be repeated within 48 hours if the local pp65 antigenemia result is positive, the central laboratory PCR viral load result is negative and the subject is treated with CMV-specific AVT.

The plasmids in the study vaccine may interfere with certain PCR assays. If a PCR assay is used that amplifies the genes UL55/gB or UL83/pp65, this could result in either a false positive viral load or unblinding of the study subject.

Sites that are determined to have unacceptable PCR assay in their local laboratory but were able to determine a Sponsor deemed acceptable work practice flow for utilizing another acceptable local assay or methodology will be allowed to participate in the trial.

Note: Local CMV plasma viral load testing may be performed only at a local laboratory approved by the Sponsor.

Adjudicated CMV-specific AVT will be assessed for subjects up to 1 year following transplant through the primary study period (through day 365/V14).

Immunologic response will be assessed in all subjects at V2, V5, V8, V9, V11 and V14. If the visit coincides with a study drug administration, the sample will be drawn prior to injection. In addition, if a CMV seropositive donor has consented and is participating in the optional donor cell sub-study, a 20 mL peripheral blood sample from the donor will be obtained prior to the stem cell donation for recipients participating in the main trial to test for CMV-specific T-cells. For those donating peripheral stem cells, the donor blood sample is to be collected prior to the initiation of mobilization therapy (e.g., granulocyte colony stimulating factor [G-CSF]).

Subjects will be evaluated for local reactogenicity and systemic signs and symptoms for 60 minutes (±10 minutes) after each injection. Subjects will be directly observed for 15 minutes following the injection for any injection-related side effects, then remain in a designated area at the study site for 45 minutes so that side effects can be noted and treated, if

necessary. Local reactogenicity will also be evaluated for each of the 7 consecutive days following each injection beginning approximately 24 hours after the dose (day 1-7 post dose). The assessments for each of the 7 consecutive days following an injection will be done by the subject and reported to the site via diary. When reactogenicity is reported as an AE, it must be followed until resolution or medically stable. All local reactions ≥ Grade 3 [Appendix 7] require confirmation by a health care professional.

Subjects will be contacted after the last injection of vaccine through 5.5 years post-transplant for long-term safety related to the DNA vaccine. Items to be assessed by telephone interview and available patient records include mortality, development of any new/recurrent cancer, development of infection requiring hospitalization or resulting in death, and erythema and/or induration at the injection sites [Appendix 20].

All AEs and SAEs will be collected from signing of the informed consent through 30 days post last dose of study drug. All events requiring adjudication, Grade 3 and higher AEs as well as SAEs will be collected from 31 days post last dose of study drug through 1 year post-transplant. A change in medical status or medical history from time of signing of informed consent through first dose of study drug is to be reported as an AE or SAE as appropriate.

An independent Data Monitoring Committee (DMC) will be chartered to oversee safety issues and futility analysis.

An Adjudication Committee (AC) will be chartered to adjudicate all cases of CMV EOD, initiation of CMV-specific AVT, and cause of death.

2.2.2 Dose Rationale

Efficacy and safety data was collected from normal healthy subjects in a phase 1 trial as well as HCT recipients in a phase 2 clinical trial (VCL-CB01-101 and CB01-202, respectively). The dose used in the phase 2 study was 1 mL of 5 mg/mL and 4 doses were given over approximately a 6 month time period. The dose and dose schedule appeared to be well tolerated.

The first dose of vaccine is scheduled to be administered at -14 to -3 days prior to transplant, and within 72 hours prior to the start of conditioning therapy. The first dose is scheduled during this time frame to ensure protein expression at the time of infusion of the donor stem cells as animal models demonstrate that the peak protein expression after injection of a DNA plasmid is between 3 and14 days. This dose is given prior to the start of conditioning therapy as protein expression may be decreased because of a catabolic state in the patient after the start of conditioning therapy. The second dose of vaccine is scheduled as close to Day 14 after transplant as possible to ensure protein expression and development of a protective immune response at the expected time of the first viremic episodes, which is approximately 30 days after transplant. Finally, doses are scheduled for 60, 90 and 180 days after transplant to help protect against late-onset viremia.

2.3 Variables

2.3.1 Primary Variable

The primary endpoint of the study is the composite of overall mortality and CMV EOD through 1 year post-transplant.

2.3.2 Secondary Variables

The 2 key secondary endpoints are:

- Time to first protocol-defined CMV viremia [CMV plasma viral ≥ 1000 IU/mL as assessed by the central laboratory] through 1 year post-transplant.
- Time to first adjudicated CMV-specific AVT through 1 year post-transplant.

Additional secondary endpoints are:

- Time to first CMV-specific AVT for protocol-defined CMV viremia[CMV plasma viral load ≥ 1000 IU/mL as assessed by the central laboratory] or CMV EOD through one year post-transplant.

2.3.3 Exploratory Variables

- Clinical outcomes:
  - Overall mortality
  - Incidence of CMV EOD
  - Incidence of Grade 3-4 aGVHD
  - Incidence of severe cGVHD
  - Incidence of grade 3 treatment-emergent viral, bacterial, or fungal infections other than CMV
  - Maximum grade of aGVHD
  - Maximum cGVHD score
  - Relapse mortality (death due to relapse of the subject's primary disease)
  - Non-relapse mortality (death due to causes unrelated to the subject's primary disease)
  - Incidence of graft rejection
  - Incidence of failure to engraft
  - Time to engraftment
  - Time to platelet recovery
  - Incidence of relapse of primary disease requiring therapy.
  - Number of episodes of protocol-defined CMV viremia [CMV plasma viral load ≥ 1000 IU/mL by the Abbott RealTime CMV assay as assessed by the central laboratory]
  - Time to Grade 3 treatment-emergent viral, bacterial, or fungal infections other than CMV
- Duration of hospitalization(s)
- Health economic/resource utilization

- Subjects' quality of life (QOL) variables (EQ-5D, Functional Assessment of Cancer Therapy – Bone Marrow Transplant [FACT-BMT], and Work Productivity and Activity Impairment Questionnaire: General Health [WPAI: GH])
- Immunogenicity variables
  - T-cell responses to pp65
  - gB-specific antibody levels

2.3.4 Safety Assessments

- Vital signs
- Adverse events (AEs)
- Local reactogenicity signs and symptoms using both the NCI-CTCAE Version 4.03 grading scale and the protocol-specified reactogenicity scale [Appendix 7]
- Clinical laboratory assessments as defined in [Appendix 1]
- Physical examination (PE)

2.3.5 Pharmacogenomics (PGx) Assessment (Optional)

An optional PGx saliva sample will be collected during the baseline visit (V2) after randomization but prior to the first dose of study drug. These may be assessed retrospectively to include:

- Exploratory analysis of the relationship between genes potentially involved in immunogenicity and response to ASP0113 in terms of efficacy and/or safety.
- Exploratory analysis of the relationship between genes of relevance to toxicity and/or safety issues and response to ASP0113 with respect to safety.

Please refer to [Appendix 19] (PGx Sub-Study) for additional information on the retrospective PGx sub-study associated with this study.

3 STUDY POPULATION

3.1 Selection of Study Population

This study will randomize HCT recipients who are at least 18 years of age or of legal age of consent and are CMV-seropositive.

Subjects will be enrolled in the trial based on positive CMV serostatus, which will be CMV IgG antibody detection by the local laboratory. Testing will be performed prior to randomization. Presence of anti-CMV IgG antibodies will indicate a positive CMV status.

3.2 Inclusion Criteria

A subject is eligible for the study if all of the following apply:

1. Institutional Review Board (IRB)/Independent Ethics Committee (IEC)-approved written informed consent and privacy language as per national regulations (e.g., Health Insurance Portability and Accountability Act [HIPAA] Authorization for U.S. sites) must be obtained from the subject or legally authorized representative prior to any study-specific related procedures (including withdrawal of prohibited medication, if applicable).
2. Subject is a male or female subject who is at least 18 years old or the legal age of consent (whichever is greater).
3. Subject is a CMV-seropositive HCT recipient as confirmed by local laboratory at screening.
4. Subject is planned to undergo either of the following:
    a. Sibling Donor Transplant - 7/8 HLA-A, -B, -C, -DRß1 match utilizing high resolution typing or 8/8 HLA-A, -B, -C, -DRß1 match utilizing low or high resolution typing.*
    b. Unrelated Donor Transplant - 7/8 or 8/8 HLA-A, -B, -C, -DRß1 match utilizing high resolution typing.*
    * A minimum of 8 allele matching is required. Subjects with matches performed at more than 8 alleles are included if they have 7/8 or 8/8 match at the HLA-A, -B, -C, -DRß1 alleles.
5. Subject is scheduled to receive an allogeneic peripheral blood stem cell or bone marrow transplant (BMT) for the treatment of hematologic disorders as indicated in Inclusion Criterion 6.
6. Subject has 1 of the following underlying diseases:
    a. Acute myeloid leukemia (AML), with or without a history of myelodysplastic syndrome (MDS), in first or second complete remission (CR) [Appendix 6] or in early relapse (< 20% blasts in bone marrow with no circulating blasts in peripheral blood and no extramedullary leukemia).
    b. Acute lymphoblastic leukemia (ALL), in first or second CR [Appendix 6].
    c. Acute undifferentiated leukemia (AUL) in first or second CR [Appendix 6].
    d. Acute biphenotypic leukemia in first or second CR [Appendix 6].
    e. Chronic myelogenous leukemia (CML) in either chronic or accelerated phase.
    f. Chronic lymphocytic leukemia.
    g. One of the following MDS defined by the following:
        i. Refractory anemia with evidence of dysplasia.
        ii. Refractory anemia with ringed sideroblasts.
        iii. Refractory cytopenia with multilineage dysplasia.
        iv. Refractory cytopenia with multilineage dysplasia and ringed sideroblasts.
        v. Refractory anemia with excess blasts-1 (5%-10% blasts).
        vi. Refractory anemia with excess blasts-2 (10%-20% blasts).

vii. MDS, unclassified.
viii. MDS associated with isolated deletion (5q).
ix. Chronic myelomonocytic leukemia.

h. Primary or secondary myelofibrosis without leukemic transformation except if Dynamic International Prognostic Scoring System category of high or intermediate-2 [Appendix 5].
i. Lymphoma (including Hodgkin's) with chemosensitive disease (≥ 50% response to chemotherapy).

7. Female subject must be either:
   - Of non-childbearing potential
     - Postmenopausal (defined as at least 1 year without any menses) prior to screening, and if < 50 years of age and not documented to be surgically sterile must have a negative urine or serum pregnancy test at screening, or
     - Documented surgically sterile or status post-hysterectomy (at least 1 month prior to screening), or
   - Or, if of childbearing potential
     - Must have a negative urine or serum pregnancy test at screening, and
     - If heterosexually active, must use at least 1 form of birth control* (which must be a barrier method) starting at screening and through the primary study period.
8. Female subject must not be breastfeeding at screening, through the treatment period and through the primary study period.
9. Female subject must not donate ova starting at screening, through the treatment period and through the primary study period.
10. Male subject and their female spouse/partners who are of childbearing potential must be using highly effective contraception consisting of 2 forms of birth control* (1 of which must be a barrier method) starting at screening, through the treatment period and through the primary study period.

    * Acceptable forms include:
    - Consistent and correct usage of established oral contraception.
    - Established intrauterine device (IUD) or intrauterine system (IUS).
    - Barrier methods of contraception: condom or occlusive cap (diaphragm or cervical/vault caps) with spermicidal foam/gel/film/cream/suppository <Not applicable to sites in Japan: with spermicidal foam/gel/film/cream/suppository>
    - For sites in Japan: calendar-based contraceptive methods (Knaus-Ogino or rhythm method)
11. Male subject must not donate sperm starting at screening, through the treatment period and through the primary study period.
12. Subject is willing to comply with the protocol.

13. Subject agrees not to participate in another interventional study while on treatment. New regimens of approved chemotherapeutic drugs or antibodies, conditioning drugs, irradiation and T-cell depleting antibodies, except alemtuzumab, are allowed.

Waivers to the inclusion criteria will NOT be allowed.

3.3 Exclusion Criteria

A subject will be excluded from participation if any of the following apply:

1. Subject has active CMV disease or infection or has received treatment for active CMV disease or infection within 3 months (90 days) prior to transplant.
2. Subject has planned CMV prophylactic therapy with antiviral drugs or CMV-specific immunoglobulins from randomization through the primary study period completion/day 365 visit (V14).
3. Subject has a modified hematopoietic cell transplant comorbidity index (HCT-CI) score ≥ 4 [Appendix 4].
4. Subject is known to be positive for human immunodeficiency virus (HIV), hepatitis B surface antigen or hepatitis C RNA PCR.
5. Donor CMV serostatus is unknown.
6. Subject has received any of the following substances or treatments:
    a. Alemtuzumab within 60 days prior to transplant, including conditioning regimen. Subjects for whom treatment with alemtuzumab is planned at any time from 60 days prior to through 1 year post-transplant must not be enrolled in the trial.
    b. T-cell depletion of donor cell product.
    c. Administration of a CMV vaccine, including any prior exposure to ASP0113.
7. Subject has received an allogeneic stem cell transplant within 1 year prior to transplant (subjects who have received a prior autologous transplant are allowed).
8. Subject has a current malignancy in addition to the malignancy being treated for the study or the subject has a history of any other malignancy (within the past 5 years prior to screening) except non-metastatic basal or squamous cell carcinoma of the skin that has been treated successfully or cancer in situ of the cervix uteri that has been handled by local surgery.
9. Subject has an unstable medical or psychiatric condition, including a history of illicit drug(s) or alcohol abuse that the Investigator believes will interfere with protocol requirements.
10. Subject has had an allergic reaction to any component of the vaccine. Subject has had an allergic reaction to aminoglycosides as kanamycin is used in the manufacturing process of the vaccine.

11. Subject has participated in any interventional clinical study or has been treated with any investigational research products within 30 days or 5 half-lives, whichever is longer, prior to the initiation of screening. (Investigational research products are considered those products that have not been approved for any indication in the country where the subject is enrolled. New regimens of approved chemotherapeutic drugs or antibodies, conditioning drugs, irradiation and T-cell depleting antibodies, except alemtuzumab, are allowed.)
12. Subject who has received a prior HCT and has residual cGVHD.
13. Subject who is scheduled to have a cord blood transplant or a haploidentical transplant.
14. Subject has a platelet count of less than 50000 mm^3 within 3 days prior to randomization (platelet transfusions are allowed). Results received in an equivalent local unit of measure must be converted to SI units.
15. Subject has aplastic anemia or multiple myeloma.
16. For sites in Japan only: Other subjects considered ineligible by the Investigator/sub-Investigator.

Waivers to the exclusion criteria will NOT be allowed.

3.4 Contraception and Pregnancy Avoidance

During the screening period of the study and at the time of randomization, a urine or serum pregnancy test will be performed on all women of childbearing potential and all women who are of non-childbearing potential but are < 50 years of age and not documented to be surgically sterile. Subsequently, urine or serum pregnancy tests will be performed on the same day and prior to each dose, at visit 13 and at the primary study period completion/ day 365 visit (V14). All pregnancy tests will be done locally. For subjects who receive mycophenolate mofetil, additional pregnancy testing must be done in accordance with local regulatory requirements.

Subjects who become pregnant or have a positive pregnancy test during the treatment period will be withdrawn from further study treatment [Section 3.5].

Female subjects or partners of male subjects who become pregnant during the study will be followed through delivery. Pregnancy of female subjects or female partners of male subjects from first dose through 1 year post-transplant will be reported as an SAE [Section 5.5.9].

3.5 Discontinuation Criteria for Individual Subjects

A subject discontinued from study drug treatment is a subject who enrolled in the study and for whom study drug treatment is terminated prematurely for any reason.

A subject is free to withdraw from the study drug treatment and/or study follow-up for any reason and at any time without giving reason for doing so and without penalty or prejudice. This is referred to as withdrawal of consent if the subject does not allow efforts to be made even to assess survival status. Withdrawal of consent can only occur if it is subject-initiated.

The Investigator is also free to terminate a subject's study drug treatment at any time if the subject's clinical condition warrants it and the Investigator deems that further treatment is not in the best interest of the subject.

Study drug administration and participation in the study will continue for subjects when a non-fatal endpoint is met.

If a subject fails to receive 1 or more doses of study drug after the first injection, but is able to continue in the trial, the subject's participation in the trial should not be terminated.

Study Discontinuation

Subjects will be withdrawn from study and will receive no further follow-up except for mortality (at day 365 post-transplant) through public records/sources if the following occurs:

- Withdrawal of consent by subject

Subjects will be withdrawn from study and will receive no further follow-up if the following occurs:

- Subject did not receive first dose of study drug

For a subject who prematurely withdraws from the study (discontinues treatment with no continued follow-up) the study completion visit must be completed within 14 days of study withdrawal.

Treatment Only Discontinuation

Subjects will be withdrawn from study drug treatment if any of the following occur. However, they are to continue to be followed according to the protocol Schedule of Assessments [Table 1] with exception of the dose follow-up visits (i.e. V3, V6, V8, V10, and V12).

- Investigator's decision that further treatment is not in the best interest of the subject.
- Pregnancy (female subjects only).
- Failure to engraft.
- Local reactogenicity with a Grade ≥ 3 based on the criteria defined in [Appendix 7] and confirmed by a health care professional.
- Anaphylaxis with a Grade ≥ 3 based on NCI-CTCAE Version 4.03.
- Seizure considered possibly or probably related to study drug treatment.
- Participation in another interventional trial for an investigational research product (investigational research products are considered those products that have not been approved for any indication in the country where the subject is enrolled).

If a subject is withdrawn from study drug treatment and is unable to comply with study procedures as defined in the Schedule of Assessments, the subject must be asked if they are willing to be followed for SAEs through 30 days of last dose of study drug treatment, to attend the scheduled final study visit (V14), to be assessed for CMV EOD, and/or to be followed for mortality. They must also be asked if they are willing to be followed through the long term follow-up period.

Subjects who discontinue from the study after one or more doses of study drug will not be replaced. Subjects who are randomized but never receive study drug will be replaced, to a total of approximately 500 dosed subjects.

4 STUDY PRODUCTS

4.1 Description of Study Products

4.1.1 Test Product

ASP0113 contains 2 closed circular plasmid macromolecules. The plasmids are purified from cellular components and formulated with CRL1005 poloxamer and BAK, a cationic surfactant, in PBS. The combination of CRL1005 and BAK produces a thermodynamically stable self-assembled particulate system with a defined particle size, surface charge and stability profile at room temperature. BAK is used to control and maintain the particle size distribution of the formulation.

ASP0113 will be supplied by the Sponsor as a frozen solution in single dose 2-mL vials containing 1.3 mL of 5 mg/mL ASP0113. ASP0113 is packaged in a cardboard secondary container.

ASP0113 is a milky white suspension at room temperature, and clear at temperatures below the cloud point of CRL1005 (4°C - 7°C). ASP0113 is to be stored frozen at -25°C ± 10°C.

Detailed instructions for the preparation and administration of ASP0113 will be provided in the Pharmacy Manual.

4.1.2 Comparative Product

Placebo will be supplied by the Sponsor in 2-mL vials containing PBS. It is a clear colorless liquid. Placebo is to be stored frozen at -25°C ± 10°C.

Detailed instructions for the preparation and administration of placebo will be provided in the Pharmacy Manual.

4.2 Packaging and Labeling

All medication used in this study will be prepared, packaged, and labeled under the responsibility of a qualified person at Astellas US Technologies, Inc. (AUST) or designee in accordance with AUST Standard Operating Procedures (SOPs), Good Manufacturing Practice guidelines, ICH Good Clinical Practice (GCP) guidelines, and applicable local laws/regulations.

4.3 Study Drug Handling

Current International Conference on Harmonization (ICH) of Technical Requirements for Registration of Pharmaceuticals for Human Use GCP Guidelines require the Investigator to ensure that study drug deliveries from the Sponsor are received by a responsible person (e.g., pharmacist), and

- That such deliveries are recorded.

- That study drug is handled and stored according to labeled storage conditions,
- That only study drug with appropriate expiry/retest is dispensed to study subjects in accordance with the protocol, and
- That any unused study drug is returned to the Sponsor or standard procedures for the alternative disposition of unused study drug are followed with prior Sponsor approval.

Drug inventory and accountability records for the study drugs will be kept by the Investigator/ pharmacist. Study drug accountability throughout the study must be documented. The following guidelines are therefore pertinent:

- The Investigator agrees to not supply study drugs to any persons except the subjects in this study.
- The Investigator/pharmacist will keep the study drugs in a pharmacy or other locked and secure storage facility under controlled storage conditions, accessible only to those authorized by the Investigator to dispense these test drugs.
- A study drug inventory will be maintained by the Investigator/pharmacist. The inventory will include details of material received and a clear record of when they were dispensed and to which subject.
- At the conclusion or termination of this study, the Investigator/pharmacist agrees to conduct a final drug supply inventory and to record the results of this inventory on the Drug Accountability Record. It must be possible to reconcile delivery records with those of used and returned medication. Any discrepancies must be accounted for. Appropriate forms of deliveries and returns must be signed by the person responsible.
- Used or unused study drug must be returned to the depot for destruction unless prohibited by local policy. In this instance, study drug may be destroyed at the study center according to standard institutional procedures and preferably after drug accountability has been conducted by the Sponsor or representative. Should this occur, a copy of the standard institutional procedure for destroying investigational drugs will be provided to the Sponsor. Upon destruction, a copy of the certificate of destruction must be provided for the Sponsor files and site drug accountability records.

For sites in Japan:

The head of the study center or the study drug storage manager should take accountability of the study drugs as following issues.

- The study drug storage manager should store and take accountability of the study drugs in conforming to the procedures for handling the study drugs written by the Sponsor.
- The study drug storage manager should prepare and retain records of the study drug's receipt, the inventory at the study center, the use by each subject, and the return to the Sponsor or alternative disposal of unused study drugs. These records should include dates, quantities, batch/serial numbers, expiration dates (if applicable), and the subject identification codes.

The study drug storage manager should prepare and retain records that document adequately that the subjects were provided the doses specified in the protocol, and reconcile all the study drugs supplied from the Sponsor.

4.4 Blinding

4.4.1 Blinding Method

Subjects will be assigned to a double-blind treatment arm in the order in which they are entered into the interactive response technology randomization system. Subjects will receive their allocated treatment assignment according to a computer-generated randomization schedule prepared by the Sponsor or designee prior to the start of the study. (Refer to [Section 4.5]).

Only the pharmacist and designated staff, including the person(s) injecting the vaccine will be unblinded to treatment. All other study site staff will remain blinded to subjects' treatment assignment. The syringes will be masked prior to dosing to maintain the blind for subjects and all other personnel. Details regarding study blinding will be described in the Pharmacy Manual for the study.

4.4.2 Retention of the Assignment Schedule and Procedures for Treatment Code Breaking

The randomization list and study medication blind will be maintained by the IRT system vendor.

The DMC will be provided with access to the dosing assignment for periodic review of the unblinded data as documented in the DMC Charter.

Study drug treatment may be revealed only for reasons relating to the subject's safety and when critical therapeutic decisions are contingent on knowing the assigned study drug (refer to [Section 4.4.3]).

Withdrawal of a subject from the study [Section 3.5] is not a sufficient reason to break the study blind. The only reason to break the blind is whether the information gained materially alters the subsequent care of the subject. The reason to break the blind must be discussed with the Medical Monitor prior to breaking the blind. After receiving authorization for unblinding from the Sponsor, the Investigator will utilize the IRT to unblind the subject's treatment assignment. Unblinding through the interactive system may only be done by designated individuals at the site (typically the Investigator or any others specified by the Sponsor). As treatment assignment information for each subject will be kept in the site's pharmacy records, if for some reason access to the IRT is not available the Investigator may obtain this information from the Pharmacist or designee. If the clinician breaking the blind is unable to reach the Medical Monitor and an urgent safety issue with direct impact on patient care decisions warrants this action, the blind may be broken, but the Medical Monitor must be informed as soon as possible after the blind is broken.

If the blind is broken for a subject, the reason is to be documented as a written entry in the source document. Key information will be recorded at the time the blind is broken and entered in the subject's electronic case report form (eCRF). This includes the subject number, date the blind was broken, the reason, the person who requested the breaking of the blind, the name of the person who broke the blind, and the name of the APGD-US representative contacted.

4.4.3 Breaking the Treatment Code for Emergency

Breaking the treatment code, or unblinding of an individual subject's treatment assignment, may be done only for reasons relating to subject safety or when critical therapeutic decisions are contingent upon knowing the blinded study drug assignment. As noted in [Section 4.4.2], the only reason to break the blind is whether the information gained materially alters the subsequent care of the subject. The reason to break the blind must be discussed with the Medical Monitor prior to breaking the blind. If the clinician breaking the blind is unable to reach the Medical Monitor and an urgent safety issue with direct impact on patient care decisions warrants this action, the blind may be broken, but the Medical Monitor must be informed as soon as possible after the blind is broken.

If it becomes necessary to break the code during the study, the date, time and reason will be collected in the subject's eCRF and the appropriate source documents at the site.

4.4.4 Breaking the Treatment Code by the Sponsor

The Sponsor may break the treatment code for subjects who experience a Suspected Unexpected Serious Adverse Reaction (SUSAR) in order to determine if the individual case or a group of cases requires expedited regulatory reporting. A code breaking process will be provided to the limited staff who are empowered to break the codes for all SUSAR cases for regulatory reporting purposes.

4.5 Assignment and Allocation

At the screening visit, after the Informed Consent Form (ICF) has been signed, the subject will receive a subject number assignment through the IRT for use throughout the study.

Subjects who subsequently meet all inclusion and none of the exclusion criteria will be randomly assigned to receive either ASP0113 or placebo. The randomization to treatment will be equally allocated (i.e., 1:1) and stratified by donor-recipient relatedness and donor CMV serostatus. At least 30% of subjects enrolled will be donor-seronegative.

The IRT vendor will generate the randomization schedule. To obtain the randomized treatment assignment for a subject, the pharmacist or designee will utilize an IRT, which is available 7 days a week, 24 hours a day.

Randomization is to be done after the completion of the screening period, and at least -14 to -3 days prior to the anticipated transplant day (day 0). The first injection must be given within 72 hours prior to the start of conditioning therapy after randomization. After

submitting required information about the eligible subject, the drug kit number assignment will be provided.

Study drug assignment will remain blinded to all site staff except the pharmacist and designated staff.

If a subject is randomized but does not receive study drug, the subject number will not be used again. In this instance, the treatment assignment should only be known by the pharmacist and designated staff (i.e., the blind must be maintained as it is with all other subjects).

5 TREATMENTS AND EVALUATION

5.1 Dosing and Administration of Study Drugs and Other Medications

5.1.1 Dose/Dose Regimen and Administration Period

One (1) mL of 5 mg/mL ASP0113 or placebo will be administered to the subjects at the clinical site when there is no medical contraindication to an IM injection, in the deltoid muscle, alternating sides with each dose if possible. Preparation of study drug will be described in the Pharmacy Manual for the study. The syringes will be masked to maintain the study blind.

The platelet count must be ≥ 50000/ mm^3 (spontaneously or after platelet transfusion) performed by the local laboratory within 3 days prior to all study drug injections. Prior to all study drug injections, confirmation must be made that there is no medical contra-indication to an IM injection. Results received in an equivalent local unit of measure must be converted to SI units. If the subject's platelet count does not meet this threshold at the time of the visit, the subject may return for the study drug injection and local reactogenicity assessment at a later time within the allowable window for the visit. If the subject's platelet count does not meet this threshold during the visit window, the dose must be skipped, and the reactogenicity assessment and dose follow-up visit(s) should not be done.

Doses will be given at the following time points:

- Dose 1 at day -14 to -3 pretransplant, within 72 hours prior to the start of chemotherapy and radiation therapy for conditioning.
- Dose 2 at day 14-40 (but not prior to day 14).
    - Dose 2 is to be administered as close to day 14 as possible. If the subject cannot be dosed by day 40 based on these criteria, V5 procedures (except study drug injection and local reactogenicity assessment) is to be completed within 5 days of day 40 (day 40 to 45) and V6 should be skipped.
- Dose 3 at day 60 (± 5 days)
- Dose 4 at day 90 (± 10 days)
- Dose 5 at day 180 (± 10 days)

5.1.2 Increase or Reduction in Dose of the Study Drugs

Dose increases or decreases are not allowed.

5.1.3 Treatment Compliance

The number of study drug doses administered will be collected in the eCRF. If a subject fails to receive 1 or more doses of study drug after the first injection, but is able to continue in the trial, the subject's participation in the trial should not be terminated.

5.1.4 Emergency Procedures and Management of Overdose

No maximum tolerated dose for ASP0113 has been identified. In the event of an adverse clinical response to ASP0113 injection, interventions should be guided by the subject's symptoms and condition.

5.1.5 Previous, Concomitant and Prohibited Medication (Drugs and Therapies)

5.1.5.1 Previous Medication (Drugs and Therapies)

Subjects who have received any of the following substances or treatments are not eligible for the study:

- Investigational research products within 30 days or 5 half-lives, whichever is longer, prior to the initiation of screening. (Investigational research products are considered those products that have not been approved for any indication in the country where the subject is enrolled. New regimens of approved chemotherapeutic drugs or antibodies and conditioning drugs, irradiation and T-cell depleting antibodies, except alemtuzumab, are allowed).
- Alemtuzumab within 60 days prior to transplant, including conditioning regimen.
- T-cell depletion of donor product.
- Administration of a CMV vaccine, including any prior exposure to ASP0113.

5.1.5.2 Concomitant Medication (Drugs and Therapies)

All concomitant medications and therapies administered from 30 days prior to transplant through 30 days post last dose of study drug will be collected on the eCRF; Concomitant medications and therapies associated with all events requiring adjudication, Grade 3 and higher AEs as well as all SAEs will be collected from 31 days post last dose of drug through 1 year post-transplant. Over-the-counter medications, and herbal remedies do not need to be captured beginning 30 days after the last dose of study drug.

5.1.5.2.1 Conditioning Regimens

Subjects are to undergo a conditioning regimen per local care for the institution at which they are enrolled. Both myeloablative and non-myeloablative (reduced intensity) conditioning regimens are allowed (see [Appendix 17]).

Note: alemtuzumab is not allowed within 60 days prior to transplant.

5.1.5.2.2 Viral Prophylaxis

Viral prophylaxis for infection other than CMV is to be administered according to standard institutional protocols. Prophylactic use of aciclovir (acyclovir), valaciclovir (valacyclovir),

or famciclovir must not exceed the following doses following transplant (day 0) through 1 year post-transplant (day 365/V14) [Kumar, 2011; Tomblyn, 2009].

Aciclovir:

- 1600 mg orally (total daily dose), or
- 500 mg/m^2/day iv (total daily dose) – The dose may be rounded up to nearest 100 mg.

Valaciclovir: 1000 mg po (total daily dose)

Famciclovir: 500 mg po (total daily dose)

These doses may NOT be exceeded for prophylaxis but may be exceeded if necessary to treat active infections post-transplant.

5.1.5.3 Prohibited Concomitant Medications (Drugs and Therapies)

The following medications and therapies are prohibited throughout the study (through the primary study period completion/day 365 visit [V14]):

- Investigational research products that have not been approved for any indication in the country where the subject is enrolled.
    - New regimens of approved chemotherapeutic drugs or antibodies; conditioning drugs, irradiation and T-cell depleting antibodies, except alemtuzumab, are allowed.
- Prophylactic use of antiviral drugs for CMV viremia from time of randomization through completion of the primary study period completion/day 365 visit (V14).
- Prophylactic use of CMV-specific immunoglobulin.
- Alemtuzumab within 60 days prior to transplant (including conditioning regimen) through completion of the primary study period completion/day 365 visit (V14) unless required for disease relapse post-transplant.

5.2 Demographics and Baseline Characteristics

5.2.1 Demographics

The subject's date of birth, sex, race, and ethnicity (USA only) will be recorded at the screening visit.

5.2.2 Medical History

A detailed medical history for each subject, including history of prior CMV, will be obtained at the screening visit. All relevant past and present conditions will be recorded for the main body systems, as well as prior surgical procedures.

A detailed description of the subject's current disease status and conditioning regimen (including doses) will be recorded at the baseline visit (V2). A determination of myeloablative vs. non-myeloablative regimens will be made by the Investigator based on the definitions included in [Appendix 17].

Any untoward medical events that occur from the time of consent and during the screening period (prior to study drug administration) will be captured as AEs in the eCRF. A change in

medical status or medical history from time of signing of informed consent through first dose of study drug is to be reported as an AE or SAE as appropriate.

5.2.3 Transplant Information

The following transplant information will be collected for each subject:

- Volume infused (in mL)
- Human leukocyte antigen (HLA) typing of the donor and recipient
- HLA cross match at time of transplant (as determined by the site's standard method of determination and if available)
- Type of transplant
- Number of CD34+ stem cells/kg infused, if available
- CMV-serostatus of the donor and recipient
- Hepatitis B virus, HCV, and EBV serostatus of the donor and recipient, if available
- Donor CD3+, CD4+, CD8+ and CD56+ cell counts from donor cell product, if available
- Blood type of the donor and recipient
- ABO cross match, if available
- Donor-recipient relatedness
- Donor gender
- Donor age
- Donor race, if available
- Donor ethnicity, if available (USA only)
- Donor product plasma volume and red blood cell (RBC) depletion status
- Donor CMV-specific T-cells quantity (optional and requires informed consent from donor)
- Number of mononuclear cells transfused, if available (for bone marrow recipients only).

5.2.4 Modified HCT-CI Score

The subject's Modified HCT-CI score must be determined during the screening period using the history and laboratory results obtained on the day of screening. Pulmonary function tests (PFTs) including forced expiratory volume and diffusing lung capacity of carbon monoxide, multi gated acquisition scans, and/or echocardiograms performed within 6 months (180 days) prior to screening are to be used for scoring pulmonary and cardiac comorbidities.

Modified HCT-CI definitions and scores are located in [Appendix 4].

5.3 Efficacy and Immunogenicity Assessments

5.3.1 CMV Plasma Viral Load Assessment

CMV plasma viral load will be assessed by Abbott's RealTi*m*e CMV assay using a fully automated system entailing extraction of CMV DNA and amplification of viral DNA with virus specific primers and probes. The device (test) used to measure CMV in subjects' plasma being developed by Abbott Molecular Inc. is investigational as it is not approved by any regulatory authorities or agencies.

A subject's viremia status will be monitored by the site on a regular schedule, as outlined in Table 1a, and at any time it is clinically indicated. Central laboratory measurements for routine surveillance of viremia will be performed at the following time points:

- Every 7 days (weekly ± 2 days) from days 0 to 100.
- Every 14 days (every other week ± 5 days) from days 101 to 180,
- Every 30 days (every 30 days ± 5 days) from days 181 to 365.

A central laboratory measurement will also be performed at the initiation of CMV-specific AVT, when clinically indicated and/or each time a local laboratory is used to assess the viral load. Upon initiation of CMV-specific AVT, plasma viral load testing will be performed at least weekly until the CMV-specific AVT is discontinued, at which point, the regularly scheduled plasma viral load assessments will resume. Please refer to the Laboratory Manual for more exact instructions.

5.3.2 Graft-Versus-Host Disease

Acute and chronic GVHD must be assessed and graded/globally scored and the Karnofsky Performance Scale (KPS) will be used to assess performance status at baseline and visits V5, V7, V9, V11, and V13 following transplant (day 0) through the primary study period completion/day 365 visit (V14). In addition, aGVHD is to be assessed weekly through day 100. Local laboratory values may be used for grading.

When a diagnosis of aGVHD is made for a subject, assessments must continue weekly (local laboratory values may be used for grading), until resolution for maximal grade and stage by key organ. When a diagnosis of cGVHD is made for a subject, the subject must return to the study center for evaluation and data collection, and assessments must be done at each regularly scheduled protocol visits, except those visits conducted by HHC, until resolution the maximum score by any assessment at the study site will also be recorded. Refer to [Appendix 11] for the criteria for the diagnosis of cGVHD.

All results of biopsies obtained for confirming the diagnosis of aGVHD and cGVHD will also be recorded.

Local laboratory values used for grading of GVHD must be entered into the eCRF.

The following grading/global scoring scales for acute and chronic GVHD will be utilized for the study:

- aGVHD: Occurrence, stage, and overall grade of aGVHD will be based on the aGVHD tables presented in [Appendix 9].
- cGVHD: Grading as limited or extensive as assessed based on definitions in [Appendix 12].
- cGVHD: Occurrence, organ scoring and global scoring of cGVHD will be based on the information presented in [Appendix 12 and Appendix 13].
- KPS: Performance status as recorded in percentages will be based on the table presented in [Appendix 22].

5.3.3 CMV EOD

CMV EOD is defined as presented in [Appendix 2]: Definition of CMV Disease.

5.3.4 Infection

Except for CMV, infection is defined as presented in [Appendix 8], Severity Grading Table and Recurrence Interval Definitions for Infections. Infection grading using both of these appendix definitions as well as the NCI-CTCAE Version 4.03 grading scale is required.

5.3.5 Cause of Death

All diagnoses at the time of death will be captured in the eCRF. Additionally, the cause of death on the subject's death certificate, if available, must be reported. The cause of death will be adjudicated by the AC.

5.3.6 Engraftment

The date of engraftment is defined as the first of 3 consecutive measurements post nadir where the peripheral blood absolute neutrophil count (ANC) is > 500/mm^3 (0.5x10^9/L). If the subject's blood neutrophil count does not nadir below 500/mm^3 (0.5x10^9/L), the day of engraftment should be recorded as day 0. Results received in an equivalent local unit of measure must be converted to SI units.

5.3.6.1 Failure to Engraft

The subject has never engrafted and has an ANC < 500/mm^3 (0.5x10^9/L).

5.3.6.2 Rejection/poor Graft Function

ANC sustained > 0.5 x 10^9/L for 3 or more consecutive days with subsequent decrease to < 0.5x 10^9/L and bone marrow examination with < 5% cellularity.

5.3.6.3 Graft Failure

Graft failure includes persistent neutropenia, < 5% donor chimerism, and ANC < 0.5x10^9/L for 3 or more consecutive laboratory values [CIBMTR Forms manual, 2007].

5.3.6.4 Platelet Recovery

The first date of 3 consecutive laboratory values obtained on different days where the platelet count was > 20 x 10^9/L. All dates must reflect no platelet transfusions in the previous 7 days. If the subject's platelet count does not nadir below 20 x 10^9/L, the day of platelet recovery should be recorded as day 0.

5.3.6.5 Incidence of Relapse of Primary Disease Requiring Therapy

Diagnoses and type of recurrence of the primary disease requiring therapy must be captured in the eCRFs. Therapies to include are chemotherapeutic drugs or antibodies, irradiation, or a second HCT.

5.3.7 Immunogenicity

Because subject inclusion is dependent on serological evidence of previous CMV infection, IgG antibodies to gB will be present prior to transplantation.

An indicator of immunogenicity elicited by the vaccine is predicted to be an increase in gB-specific antibody levels and the T-cell response to pp65 protein over time compared with the placebo group.

Two immunogenicity assessments will be performed:

1. T-cell response to viral protein pp65 using either ELISPOT assay and/or intracellular cytokine staining (ICS).
2. Antibody response to gB antigen using an ELISA-based platform.

Assessments will be done from peripheral blood mononuclear cells (T-cells) or serum samples (antibody) isolated over the course of the trial. Time points of the assessments are as follows:

- On V2, V5, V8, V9, V11 and V14.
  - Samples for T-cell assays will not be collected if the ALC is known to be ≤ 500 mm^3 by local or central laboratory measurement.
  - If the visit coincides with a study drug administration, the sample will be drawn prior to injection.

Further details will be available in the Laboratory Manual.

5.3.8 Resource Utilization and Patient Reported Outcomes

Additional assessments include the following resource utilization and patient reported outcome measures:

- EuroQol (EQ-5D)
- FACT-BMT
- WPAI:GH
- Health Economic Assessment (HEA)

The EQ-5D, FACT-BMT, and WPAI:GH will be assessed at each dosing visit, at V13, and at the primary study period completion/day 365 visit (V14). These questionnaires are to be completed by the subject prior to any other study assessments or visit procedures. Subjects are to complete these assessments in the following order: EQ-5D first, FACT-BMT second, WPAI last. For all 3 patient-reported outcome measures, i.e. EQ-5D, FACT-BMT, WPAI, the answers must come from the subject. If a subject is unable to answer a question, the rest of the questionnaire is still to be completed. If a subject is not able to provide any answers to the questions, the assessment should be skipped.

Health economic information will be collected for subjects through the primary study period completion/day 365 visit (V14) [Section 5.3.8.4].

5.3.8.1 EQ-5D

The EQ-5D is an international standardized non-disease specific (i.e. generic) instrument for describing and valuing health status. It is a measure of health-related QOL, capable of being expressed as a single index value and specifically designed to complement other health status measures. The questionnaire will be provided in the local language of the subject. The EQ-5D has 5 domains: Mobility, Self-Care, Usual Activities, Pain/Discomfort, and Anxiety/Depression. Each domain has 5 response levels (e.g., 1 = no problems, 2 = slight problems, 3 = moderate problems, 4 = severe problems, 5 = extreme problems). In addition, it has a visual analogue scale that elicits a self-rating by the respondent of his/her health status [Appendix 14].

5.3.8.2 FACT-BMT

The FACT-BMT is a self-administered instrument designed to assess multi-dimensional aspects of QOL in BMT patients. The questionnaire will be provided in the local language of the subject. It consists of the 27-item FACT-General (FACT-G) and the 23-item Bone Marrow Transplantation Subscale (BMTS). The FACT-G assesses 4 primary dimensions of QOL, including physical well-being (7 items), social/family well-being (7 items), emotional well-being (6 items), and functional well-being (7 items). A 5 point response scale ranging from 0 to 4 is used (0 = 'not at all'; 1 = 'a little bit'; 2 = 'somewhat'; 3 = 'quite a bit'; and 4 = 'very much') [Appendix 15].

5.3.8.3 Work Productivity and Activity Impairment: General Health Questionnaire

The WPAI:GH is a 6-question instrument designed to assess the effect of health problems (physical or emotional problem or symptom) on an individual's ability to work and perform regular activities [Appendix 16]. The questionnaire will be provided in the local language of the subject.

5.3.8.4 Health Economic Assessment

Health Economic Assessments (see [Appendix 21]) will include the following: hospitalizations recorded as number of days in the Intensive Care Unit, Step-down Unit, and general medical ward, (including bone marrow transplant [BMT] unit), (including emergency room [ER] visits greater than 24 hours) also non-protocol-related physician (MD/DO) visits and ER visits less than or equal to 24 hours. The information will be collected by the site via a retrospective review of the subject's medical record. HEA data will include both data being routinely captured for the subject (e.g., concomitant medications) as well as data that will be used specifically for these analyses (e.g., information on hospitalizations).

5.3.9 Optional Donor Sub-Study (Not Applicable to Sites in Japan)

For CMV seropositive donors, there is an optional sub-study for the testing of CMV-specific T-cells. A 20 mL peripheral blood sample from the donor will be obtained prior to the stem cell donation for recipients participating in the main trial. For those donating peripheral stem cells, the donor blood sample is to be collected prior to the initiation of mobilization therapy (e.g., G-CSF). Whether donors donating at institutions other than the site where the HCT will

occur will be able to participate is dependent on the capabilities of each country and subsequent institution to arrange the logistics. If the donor does not consent to participate in the sub-study, the recipient will not be impacted and will still receive the HCT and be allowed to continue in the main trial.

5.4 Safety Assessment

Safety assessments include the following:

- Vital signs
- AEs
- Local reactogenicity
- Laboratory assessments
- PE

5.4.1 Vital Signs

Vital signs (systolic and diastolic blood pressure, pulse rate, respiratory rate, temperature and weight) will be collected at all dosing visits, and at screening (V1), V13 and V14. Height will be collected at screening only.

Vital signs will be collected as follows:

- Vital signs will be collected immediately prior to injection and 60 (±10) minutes post injection (except weight) at study visits accompanied by injection.
- Vital signs will be collected once at screening (V1), and at V13 and V14.

5.4.2 Adverse Events

All AEs and serious adverse events (SAEs) will be collected from signing of the informed consent through 30 days following the last dose of study drug. All events requiring adjudication, Grade 3 and higher grade AEs as well as SAEs will be collected from 31 days post last dose of drug through 1 year post-transplant. A change in medical status or medical history from time of signing of the informed consent through first dose of study drug are to be reported as an AE, or SAE as appropriate. In this study, the day 0 HCT is not considered an AE.

Data to be recorded includes the AE, date of onset, date of resolution, intensity, action taken with respect to study drug, treatment required, relationship to study drug and outcome of the event.

Grade 3 or greater AEs ongoing at V14 will be followed up for as long as necessary to adequately evaluate the subject’s safety or until the event stabilizes. If the event resolves during the study, a resolution date will be documented on the case report form.

5.4.2.1 Local Reactogenicity Assessments

Subjects will be evaluated for local reactogenicity and systemic signs for 60 minutes (± 10 minutes) after each injection. Subjects will be directly observed for 15 minutes following the injection for any injection-related side effects, then remain in a designated area at the study site for 45 minutes so that side effects can be noted and treated, if necessary.

Local reactogenicity will also be evaluated for each of the 7 consecutive days following each injection. The assessments for each of the 7 consecutive days following an injection will be done by the subject and reported to the site via diary. When reactogenicity is reported as an AE, it must be followed until resolution or medically stable. All local reactions ≥ Grade 3 [Appendix 7] require confirmation by a health care professional.

Grading of reactogenicity will be done using the protocol-specified reactogenicity scale [Appendix 7]. The daily maximum measurement of local reactogenicity signs will be recorded.

5.4.3 Laboratory Assessments

Please refer to [Appendix 1] for a list of laboratory tests to be performed during the study.

Blood and urine samples will be collected for the following laboratory assessments:

- Urine or serum pregnancy testing for all females of childbearing potential and all females who are of non-childbearing potential but are < 50 years of age and not documented to be surgically sterile will be performed at the screening visit. Subsequently, a urine or serum pregnancy test will be performed at V2, V5, V7, V9, V11, V13, and at the primary study period completion/day 365 visit (V14). All pregnancy tests will be done locally and performed on the same day and prior to study drug administration on visits that coincide.
- CMV IgG antibody assay will be measured at screening by the local laboratory. CMV plasma viral load will be monitored as outlined in [Section 5.3.1].
    - Local laboratory values for CMV viral load must be collected in the eCRF.
- Hematology (RBC, white blood cells [total leukocytes], hemoglobin, hematocrit, platelets [thrombocytes], neutrophils [ANC], eosinophils, basophils, lymphocytes, monocytes and blast cells) will be measured at V1, V2, and V5-V12.
- Platelet count must be performed locally and within 3 days prior to study drug dosing. For dose 1, it must be done within 3 days prior to randomization.
- Biochemistry (sodium, potassium, calcium, chloride, glucose, creatinine, CPK/CK, total protein, albumin) will be measured at V1, V2, and V5-V12.
    - If local or central CPK/CK levels are 5x the ULN at any time during the primary study period, a repeat laboratory draw must be performed and sent to the central laboratory for testing. In addition, these local laboratory levels must be captured in the eCRF for the subject.
    - Local laboratory values used for grading of GVHD must also be captured in the eCRF for the subject.
- Hepatic profile (total bilirubin, direct bilirubin, alkaline phosphatase, aspartate amino-transferase [AST], alanine aminotransferase [ALT]) will be measured at V1, V2 and V5-V12. See [Appendix 18] for follow-up of liver function tests (LFTs) beyond the ULN
    - If local or central AST or ALT are > 20 x ULN, or bilirubin is found to be ≥ 10 x ULN at any time during the primary study period, a repeat laboratory draw

must be performed and sent to the central laboratory for testing. In addition, these local laboratory levels must be captured in the eCRF for the subject.

Clinically significant laboratory results must be followed until resolution or until they are no longer clinically significant or are medically stable.

- Immunogenicity: gB-specific antibody levels and T-cell responses to pp65.

A saliva sample will be collected at V2 after randomization but prior to the first dose of study drug for subjects who agree to participate in the optional PGx substudy.

For CMV seropositive donors who consent to participate in the optional substudy, a 20 mL peripheral blood sample from the donor will be obtained prior to the stem cell donation for recipients participating in the main trial to test for CMV-specific T-cells. For those donating peripheral stem cells, the donor blood sample is to be collected prior to the initiation of mobilization therapy (e.g., G-CSF).

5.4.3.1 Abnormal LFTs

See [Appendix 18] (Liver Safety Monitoring and Assessment) for information on monitoring and assessment of abnormal LFTs.

5.4.3.2 Bronchoalveolar Lavage Fluid

In the event that a bronchoalveolar lavage (BAL) sample is obtained for CMV pneumonia evaluation some of this sample will be sent to the central laboratory for storage and possible central reading.

5.4.4 Physical Examination

Complete PE will be performed at the screening and baseline visits (V1 and V2) and at the primary study period completion/day 365 visit (V14). The PE will include the following: an examination of the skin, general appearance, neck (including thyroid), eyes, ears, nose, throat, lymph nodes, chest (lungs), heart, abdomen, musculoskeletal system, neurological system and any additional assessments needed to establish baseline status or evaluate symptoms or AEs. At all other visits where a PE is performed, symptom-directed PEs may be done. Performance status will be assessed using the KPS [Appendix 22] at baseline visit (V2).

The Investigator or qualified medical personnel who routinely perform these evaluations in this patient population will conduct the examination, determine findings, and assess any abnormalities as to clinical significance.

5.4.5 Long-Term Follow-up

Subjects will be contacted after the primary study period (day 365) through 5.5 years post-transplant for long-term safety related to the DNA vaccine. Items to be assessed by telephone interview and available patient records include mortality, development of any new/recurrent cancer, development of infection requiring hospitalization or resulting in death, and erythema and induration at the sites of study drug injections [Appendix 20].

During the long-term follow-up period, if an SAE is identified and deemed possibly or probably related to the study medication, an SAE report on the SAE worksheet must be sent to the Sponsor per [Section 5.5.6] for all sites except those in Japan. For study sites in Japan, an SAE report on the JUTOKUNA YUUGAIJISHOU HOUKOKUSHO must be sent to delegated CRO per [Section 5.5.3].

5.5 Adverse Events and Other Safety Aspects

5.5.1 Definition of Adverse Events

An AE is defined as any untoward medical occurrence in a subject administered a study drug or has undergone study procedures and which does not necessarily have a causal relationship with this treatment. An AE can therefore be any unintended sign (including an abnormal laboratory finding), symptom, or disease temporally associated with the use of a medicinal (investigational) product, whether or not related to the medicinal (investigational) product.

Some countries may have additional local requirements for events that are required to be reported as AEs or in an expedited manner similar to an SAE. In these cases, it is the investigator's responsibility to ensure these and other reporting requirements are followed and that the information is appropriately recorded in the eCRF accordingly.

In this study, the day 0 HCT is not considered an AE. All AEs will be collected from signing of the informed consent through 30 days after last dose of study drug. Only events requiring adjudication, Grade 3 and higher AEs will be collected from 31 days after last dose of study drug through 1 year post-transplant.

An abnormality identified during a medical test (e.g., laboratory parameter, vital sign, electrocardiogram [ECG] data, PE) should be defined as an AE only if the abnormality meets 1 of the following criteria:

- Induces clinical signs or symptoms.
- Requires active intervention.
- Requires interruption or discontinuation of study medication.
- The abnormality or investigational value is clinically significant in the opinion of the Investigator.

5.5.2 Adverse Events of Special Interest

The following definitions must be used to properly record and report adverse events for the study.

5.5.2.1 Malignancy

All new malignancies or relapses of prior malignancies and relapses of primary malignancy requiring treatment will be recorded.

5.5.2.2 Sinusoidal Obstruction Syndrome (SOS) [Hepatic Veno-occlusive Disease (VOD)]

In the absence of a histological diagnosis, recipients must fulfill the criteria below for a diagnosis of SOS.

No other identifiable cause for liver disease with at least 2 or more of the following clinical signs and symptoms:

- Jaundice (bilirubin >2 mg/dL or > 34 μmol/L).
- Hepatomegaly with right upper quadrant pain.
- Ascites and/or weight gain (> 5% over baseline).

5.5.2.3 Adverse Events Commonly Associated with Vaccines

The following AEs have been reported with other vaccines and will be subject to specific monitoring:

- Acute local vaccines reactions: local pain, redness, swelling or induration (as defined in Section 5.4.2.1)
- Allergic and demyelination reactions

5.5.3 Definition of SAEs

An AE is considered "serious" if, in the view of either the Investigator or Sponsor, it results in any of the following outcomes:

- Results in death.
- Is life threatening (an AE is considered "life-threatening" if, in the view of either the Investigator or Sponsor, its occurrence places the subject at immediate risk of death. It does not include an AE that, had it occurred in a more severe form, might have caused death).
- Results in persistent or significant disability/incapacity or substantial disruption of the ability to conduct normal life functions.
- Results in congenital anomaly, or birth defect.
- Requires inpatient hospitalization or leads to prolongation of hospitalization (hospitalization for treatment/observation/examination caused by AE is to be considered as serious).
- Results in drug exposure during pregnancy.
- Other medically important events.

In this study, the day 0 HCT is not considered an SAE.

Medical and scientific judgment must be exercised in deciding whether expedited reporting is appropriate in other situations, such as important medical events that may not be immediately life-threatening or result in death or hospitalization but may jeopardize the subject or may require intervention to prevent 1 of the other outcomes listed in the definition above. These events, including those that may result in disability/incapacity, are also usually considered serious. Examples of such events are intensive treatment in an ER or at home for allergic bronchospasm; blood dyscrasias or convulsions that do not result in hospitalization; or development of drug dependency or drug abuse.

Safety events of interest on the medicinal products administered to the subject as part of the study (e.g., study drug, comparator, background therapy) that may require expedited reporting and/or safety evaluation include, but are not limited to:

- Overdose of a Sponsor medicinal product
- Suspected abuse/misuse of a Sponsor medicinal product
- Inadvertent or accidental exposure to a Sponsor medicinal product
- Medication error involving a Sponsor product (with or without subject/patient exposure to the Sponsor medicinal product, e.g., name confusion)

All of the events of interest noted above must be recorded on the eCRF. Any situation involving these events of interest that also meets the criteria for an SAE must be recorded on the AE page of the eCRF and marked 'serious' and the SAE worksheet (for sites in Japan: JUTOKUNA YUUGAIJISHOU HOUKOKUSHO) completed.

The Sponsor has a list of events that they classify as "always serious" events. If an AE is reported that is considered to be an event per their classification as "always serious", additional information on the event may be requested.

SAEs as listed in [Appendix 3] (Common Serious Adverse Events and Endpoint Events) must be reported to Astellas within 24 hours, but a single occurrence of SAEs listed in [Appendix 3] may be excluded from expedited reporting from Astellas to the regulatory agencies or/and competent authorities (CAs) (See [Appendix 3] for more information).

If a subject becomes pregnant during treatment, this must be reported as if it were a SAE. Refer to [Section 5.5.9], Procedure in Case of Pregnancy.

5.5.4 Criteria for Causal Relationship to the Study Drug

AEs that fall under either "Possible" or "Probable" must be defined as "adverse events whose relationship to the study drugs could not be ruled out."

Causal Relationship to the Study Drug	Criteria for Causal Relationship
Not Related	A clinical event, including laboratory test abnormality, with a temporal relationship to drug administration which makes a causal relationship improbable, and/or in which other drugs, chemicals or underlying disease provide plausible explanations.
Possible	A clinical event, including laboratory test abnormality, with a reasonable time sequence to administration of the drug, but which could also be explained by concurrent disease or other drugs or chemicals. Information on drug withdrawal may be lacking or unclear.
Probable	A clinical event, including laboratory test abnormality, with a reasonable time sequence to administration of the drug, unlikely to be attributed to concurrent disease or other drugs or chemicals, and which follows a clinically reasonable response on re-administration (rechallenge) or withdrawal (dechallenge).

5.5.5 Criteria for Defining the Severity of an Adverse Event

The NCI-CTCAE Version 4.03 (including laboratory abnormalities) will be used to measure the severity of AEs, except local reactogenicity which will use both the NCI-CTCAE Version 4.03 and the protocol-specific reactogenicity scale [Appendix 7].

5.5.6 Reporting of SAEs

For all countries, except Japan, in the case of an SAE, the Investigator must contact the Sponsor by telephone or fax immediately (within 24 hours of awareness).

The Investigator must complete and submit an SAE Worksheet containing all information that is required by the Regulatory Authorities to the Sponsor by fax or email immediately (within 24 hours of awareness). If the faxing or emailing of an SAE Worksheet is not possible or is not possible within 24 hours, the local drug safety contact must be informed by phone.

For sites in Japan only: In the case of an SAE, the Investigator/Sub- investigator must report the SAE to the head of the study center and also contact the delegated CRO by telephone or fax immediately (within 24 hours of awareness).

The Investigator must complete and submit JUTOKUNA YUUGAIJISHOU HOUKOKUSHO containing all information that is required by the regulatory authorities to the delegated CRO and head of the study center by fax immediately (within 24 hours of awareness). If faxing of JUTOKUNA YUUGAIJISHOU HOUKOKUSHO is not possible, or is not possible within 24 hours, the delegated CRO should be informed by phone.

For contact details for each country/region, see Section II Contact Details of Key Sponsor's Personnel. Please fax the SAE Worksheet (Specific to sites in Japan: JUTOKUNA YUUGAIJISHOU HOUKOKUSHO) to:

PPD

For study sites outside of Japan, the Investigator must fax or email the SAE Worksheet to:

Astellas Pharma Global Development – United States
Product Safety & Pharmacovigilance
Fax number: +1 847-317-1241
International Fax: +44 800-471-5263
Email: Safety-US@astellas.com

If there are any questions, or if clarification is needed regarding the SAE, please contact the Sponsor's Medical Monitor or his/her designee [Section II].

For study sites outside of Japan: Follow-up information for the event must be sent promptly (within 7 days of the initial notification).

Full details of the SAE should be recorded on the medical records and on the eCRF.

The following minimum information is required:

- ISN/Study number,
- Subject number, sex and age,
- The date of report,

- A description of the SAE (event, seriousness of the event), and
- Causal relationship to the study drug.

The Sponsor or Sponsor's designee will submit expedited safety reports (i.e. IND Safety Reports) to the regulatory agencies (i.e. FDA) as necessary, and will inform the investigators of such regulatory reports. Investigators must submit safety reports as required by their IRB/IEC within timelines set by regional regulations (i.e. EU, (e)CTD, FDA). Documentation of the submission to and receipt by the IRB/IEC of expedited safety reports should be retained by the site.

The Sponsor/ delegated CRO will notify all investigators responsible for ongoing clinical studies with the study drug of all SAEs which require submission per local requirements IRB/IEC/the heads of the study sites.

The heads of the study sites/investigators should provide written documentation of IRB/IEC notification for each report to the Sponsor per local requirements IRB/IEC.

The study site may contact the Sponsor's Medical Monitor/Expert for any other problem related to the safety, welfare, or rights of the subject.

UNIQUE to studies enrolling subjects in EU: For SUSARs from a blinded trial, unblinded CIOMS-I report will be submitted to the authorities and IRB/IEC where required.

All observed or spontaneously reported SAEs occurring up to 12 months after transplant must be captured on the eCRFs and reported to the Sponsor (for sites in Japan: the delegated CRO) in accordance with these guidelines. All SAEs will be recorded within the source documents, on the SAE worksheets (for sites in Japan: JUTOKUNA YUUGAIJISHOU HOUKOKUSHO) which will be faxed to the Sponsor (for sites in Japan: the delegated CRO), and collected in the eCRF. AEs leading to subject discontinuation from the study will be collected in the appropriate section of the eCRF.

For the purposes of expedited safety reporting to the regulatory agencies, SAEs describing an event listed in [Appendix 3] as common and disease related or a protocol-defined endpoint (e.g., CMV diseases) may not be reported by Astellas for a single occurrence for a given subject. However, Astellas may report as appropriate aggregated cases of such events.

5.5.7 Follow-up to Adverse Events

All AEs reported during the study are to be followed up until resolved or judged to be no longer clinically significant, or until they become chronic to the extent that they can be fully characterized.

If during an AE follow-up, the AE progresses to an ″SAE″, or if a subject experiences a new SAE, the Investigator must immediately report the information to the Sponsor.

5.5.8 Monitoring of Common Serious Adverse Events and Endpoint Events

Common serious AEs are SAEs commonly anticipated to occur in the study population independent of drug exposure. SAEs classified as “common” are provided in [Appendix 3] Common Serious Adverse Events and Endpoint Events for your reference. The

list does NOT change your reporting obligations or prevent the need to report an AE meeting the definition of an SAE as detailed above. The purpose of this list is to alert you that some events reported as SAEs may not require expedited reporting to the regulatory authorities based on the classification of "common serious adverse events" as specified in [Appendix 3] Common Serious Adverse Events and Endpoint Events. The Sponsor will monitor these events throughout the course of the study for any change in frequency. Any changes to this list will be communicated to the participating investigational sites. Investigators must report individual occurrences of these events as stated in [Section 5.5.6] Reporting of Serious Adverse Events.

5.5.9 Procedure in Case of Pregnancy

If a female subject or partner of a male subject becomes pregnant from first dose through 1 year post-transplant, the Investigator must report the information to the Sponsor/delegated CRO as if it is an SAE. The expected date of delivery or expected date of the end of the pregnancy, last menstruation, estimated fertility date, pregnancy result, neonatal data, and any other clinically relevant information must be included, if available and if the mother has granted release of medical information.

The Investigator will follow the medical status of the mother, as well as the fetus, as if the pregnancy is an SAE and will report the outcome to the Sponsor/delegated CRO.

When the outcome of the pregnancy falls under the criteria for SAEs (spontaneous abortion, induced abortion, stillbirth, death of newborn, congenital anomaly [including anomaly in a miscarried fetus)], the Investigator must respond in accordance with the report procedure for SAEs. Additional information regarding the outcome of a pregnancy (which is categorized as an SAE) is mentioned below.

- "Spontaneous abortion" includes miscarriage, abortion and missed abortion.
- Death of an infant within 1 month (30 days) after birth must be reported as an SAE regardless of its relationship with the study drug.
- If an infant dies more than 1 month (30 days) after the birth, it must be reported if a relationship between the death and intrauterine exposure to the study drug is judged as "possible" by the Investigator.
- In the case of a delivery of a living newborn, the "normality" of the infant is evaluated at the birth.
- "Normality" of the miscarried fetus is evaluated by visual examination unless test results which indicate a congenital anomaly are obtained prior to miscarriage.

If during the conduct of a clinical trial, a male subject makes his partner pregnant through 1 year post-transplant, the subject will be asked to report the pregnancy to the Investigator. The Investigator will report the pregnancy to the Sponsor via an SAE/delegated CRO reporting form.

5.5.10 Supply of New Information Affecting the Conduct of the Study

When new information becomes available necessary for conducting the clinical study properly, the Sponsor will inform all investigators involved in the clinical study as well as the regulatory authorities. Investigators should inform the IRB/IEC of such information when needed.

The following 2 paragraphs are specific to sites in Japan:

1. When information is obtained regarding serious and unexpected adverse drug reactions (ADRs) (or other) that are specified in Article 273 of the Act on Securing Quality, Efficacy and Safety of Pharmaceuticals, Medical Devices, Regenerative and Cellular Therapy Products, Gene Therapy Products, and Cosmetics, in compliance with Article 80-2 Paragraph 6 of the Act described above, the Sponsor should inform all the investigators involved in the clinical study, the head of the study site, and the regulatory authorities of such information. The head of the study site who receives such information will decide whether the clinical study should be continued after hearing the opinions of the IRB. The Investigator will supply the new information to the subjects, in compliance with [Section 8.2.3.2] Supply of New and Important Information Influencing the Subject's Consent and Revision of the Written Information.
2. In addition to the above item (1), when the head of the study site receives the revisions of the IB, protocol, or written information, information on the matters covering the quality of the study drug, efficacy and safety, information necessary for conducting the clinical study properly, or documents to be examined by the IRB should be sent to the IRB.

5.5.11 Deviations from the Protocol and Other Actions Taken to Avoid Life-Threatening Risks to Subjects (FOR SITES IN JAPAN ONLY)

The following 2 paragraphs are specific to sites in Japan:

The Investigator must not deviate from or amend the protocol, excluding an emergency case for avoiding risks to the subjects. When the Investigator does not follow the protocol in order to avoid urgent risks for subjects, the Investigator should take the following actions.

1. Describe the contents of the deviation or amendment and the reasons for it in a written notice, and immediately send the document stating the deviation or amendment and the reasons to the Sponsor and the head of the study site. Keep a copy of the notice.
2. Consult with the Sponsor at the earliest possibility for cases in which it is necessary to amend the protocol. Obtain approval for a draft of the amended protocol from the IRB and the head of the study site as well as written approval from the Sponsor.

5.6 Test Drug Concentration

Not applicable.

5.7 Other Measurements, Assessments, or Methods

5.7.1 Pharmacogenomics (PGx)

During the baseline visit (V2), after randomization but prior to the first dose of study drug, subjects who consent to participate in the retrospective PGx assessment will have 2 mL of saliva collected and sent to the central laboratory.

Please refer to [Appendix 19] for more information regarding pharmacogenomic sampling and to the study Laboratory Manual for laboratory specimen handling instructions.

5.8 Total Amount of Blood

The total amount of blood to be collected for central laboratory assessments for the primary study period per subject should not exceed 63 mL at any study visit and is expected to be approximately 681 mL total for the study for all countries, except Japan. For sites in Japan: total blood volume for the study is approximately 550 mL.

For CMV seropositive donors who consent to participate in the optional donor sub-study, a 20 mL peripheral blood sample from the donor will be obtained prior to the stem cell donation for recipients participating in the main trial test for CMV-specific T-cells. For those donating peripheral stem cells, the donor blood sample is to be collected prior to the initiation of mobilization therapy (e.g., G-CSF).

Refer to the Laboratory Manual for more details regarding blood collection.

6 DISCONTINUATION

6.1 Discontinuation of Individual Subject(s)

A discontinuation is a subject who enrolled in the study and for whom study treatment is permanently discontinued prematurely for any reason. Only subjects who have received at least a single study dose and discontinued will be followed for safety.

The subject is free to withdraw from the study treatment and/or study for any reason and at any time without giving reason for doing so and without penalty or prejudice. The Investigator is also free to terminate a subject's drug treatment, if warranted by the subject's medical condition.

6.2 Discontinuation of the Study

When the Sponsor is aware of information on matters concerning the quality and safety of the study drugs, as well as other important information that may affect proper conduct of the clinical study, the Sponsor may discontinue the clinical study and send a written notice of the discontinuation along with the reasons to the Investigator (for sites in Japan: to the head of the study site), and regulatory authorities.

The DMC is responsible for periodically reviewing select accruing data for the purpose of ensuring subject safety. The DMC is an independent multidisciplinary group consisting of clinicians and a biostatistician external to Astellas that, collectively, has experience in the

management of patients undergoing HCT and other diseases, and in the conduct and monitoring of randomized clinical trials with interim analyses.

The DMC statistician will perform a futility analysis on efficacy data for presentation to the DMC at selected DMC meetings. The futility of ASP0113 will be monitored based on its effect on viremia (defined as a CMV plasma viral load ≥1000 IU/mL as assessed by the central laboratory). It is believed that viremia is a surrogate of treatment effect on clinical endpoints. Subjects receiving ASP0113 had a statistically significant reduction in viremia relative to placebo in the phase 2 trial of ASP0113. Although there are no explicit safety stopping rules, all safety issues will be thoroughly evaluated by the DMC with special attention paid to local reactogenicity and hypersensitivity cases.

Futility analysis will be performed when the first 100 subjects have been randomized, and every 3 months thereafter until the total enrollment reaches 400 subjects. Futility analysis are based on the rate of CMV viremia defined as CMV plasma load ≥1000 IU/mL as assessed by the central laboratory. Analysis will include all available viremia data obtained from the central laboratory up to the time of a cut-off point for each futility analysis. At each futility analysis, a Bayesian posterior probability that the hazard ratio for viremia of the ASP0113 treatment group to the placebo treatment group will be less than 0.75 will be calculated.

The study may stop for futility if: Posterior Probability of $(Vir_{trt}/Vir_{con} < 0.75) < 0.15$ at any time point when futility analysis is performed, including when a planned interim data analysis is conducted, where Vir_{trt} is the hazard of viremia in the ASP0113 group and Vir_{con} is the hazard of viremia in the placebo group.

The DMC will determine if the futility criterion for discontinuation of the study has been met. If the criterion has been met, sufficient information will be provided to Astellas representatives to enable them to evaluate and, if applicable, act on the recommendation of the DMC.

If an Investigator intends to discontinue participation in the study, the Investigator must immediately inform the Sponsor (UNIQUE to JP: and the head of the Study site) of the discontinuation and the reason for it.

7 STATISTICAL METHODOLOGY

This is a randomized, double-blind, placebo-controlled trial of approximately 500 subjects.

In general, all data will be summarized with descriptive statistics (number of subjects, mean, standard deviation, minimum, median and maximum) for continuous endpoints, and frequency and percentage for categorical endpoints. Statistical tests will be conducted using a significance level of 0.05. Confidence intervals will be 95%, except were otherwise indicated.

7.1 Sample Size

In the primary analysis set of a phase 2 study of the efficacy of ASP0113 in HCT, 7 subjects died and 2 additional subjects experienced CMV EOD out of 40 treated with ASP0113, for an overall failure rate of 22.5%. while among the placebo subjects 11 subjects died and 1 additional subject experienced CMV EOD out of 34, for an overall failure rate of 35.3%.

To detect the estimated difference in mortality and CMV EOD from the phase 2 study (35.3% vs. 22.5%), the study needs a sample size of at least 424 (212 per arm) to have 80% power at the 2-sided significance level of 0.050. A trial of size 500 (250 per arm) is expected to have 86% power for the composite endpoint.

All calculations are based on 1:1 randomization and used nQuery Advisor 7.0.

7.2 Analysis Set

Prior to database lock, a Final Review of Data and TLFs Meeting will be held to allow a review of the clinical trial data and to verify the data that will be used for analysis set classification. If required, consequences for the statistical analysis will be discussed and documented. A meeting to determine analysis set classifications will also be held prior to database lock.

7.2.1 Safety Analysis Set

The safety analysis set (SAF) consists of all randomized subjects who received at least 1 dose of randomized study drug. Subjects will be included in analyses using the SAF based on treatment actually received.

The SAF will be used for summaries of demographic and baseline characteristics and analysis for all safety and tolerability related variables.

7.2.2 Full Analysis Set

The full analysis set (FAS) consists of all randomized subjects who receive at least 1 dose of randomized study drug. Subjects will be included in analyses using the FAS based on assigned treatment, even if 1 or more doses of the incorrect treatment were given. The FAS will be used for summaries of demographic and baseline characteristics and for primary analysis of the efficacy variables.

7.2.3 Per Protocol Set

The per protocol set (PPS) includes all subjects of the FAS who have no major protocol violations. Criteria will be defined in the SAP.

Final judgments on exclusion of subjects from the PPS, based on protocol violations, are to be made at the Blind Data Review Meeting held prior to unblinding.

The PPS will be used for secondary analyses of efficacy variables. Also, selected demographic and baseline characteristics will be summarized for the PPS.

7.2.4 Pharmacokinetic Analysis Set

Pharmacokinetic analysis will not be performed in this study.

7.2.5 Immunogenicity Analysis Set

The immunogenicity analysis set (IAS) includes subjects who received randomized study drug for whom at least 1 post-transplant immunogenicity measurement is available.

The IAS will be used for analyses of the immunogenicity data.

7.3 Demographics and Other Baseline Characteristics

Demographics and baseline characteristics such as age, race, gender, height, weight, baseline disease status, baseline disease therapy, and donor-recipient relatedness will be summarized overall and by treatment group using each analysis set. For subjects with donors included in the optional donor substudy, numbers and percent of donors with T-cell immunity to pp65 will be summarized by treatment group.

Continuous variables will be summarized by descriptive statistics (e.g., n, mean, standard deviation, minimum, median, and maximum) and discrete variables will be summarized by the number and percentage of subjects in each category.

7.4 Analysis of Efficacy

7.4.1 Analysis of Primary Variable

7.4.1.1 Primary Analysis

In order to compare the effect on the primary composite endpoint between ASP0113 and placebo, the following null hypothesis will be constructed:

$$H_0\text{: Odds Ratio} = 1.$$

The accompanying alternative hypothesis is:

$$H_1\text{: Odds Ratio} \neq 1.$$

The 2-sided P value for the above hypothesis tests will be calculated using the CMH test stratified by the 2 stratification factors for randomization (donor-recipient relatedness and donor CMV serostatus). The effect of ASP0113 will be considered statistically significant if the CMH rejects the null hypothesis at the significance level of 0.050.

Every effort will be made to collect each subject’s status that will define the outcome of the primary endpoint. For the primary analysis, subjects with unknown survival status will be considered as having the primary endpoint event.

In addition, the Breslow-Day test will be used to assess the consistency of the odds ratio across the strata. In the event that the Breslow-Day test is significant at the 0.10 level, tabulations of the treatment effect will be provided for each stratum to assess the strength of possible treatment-by-strata interactions.

In a supportive analysis, time to first occurrence of a primary endpoint event will be analyzed in a Cox proportional hazards model where the time-to-event will be calculated in days from transplant to the earliest of death or CMV EOD. Subjects not experiencing the primary endpoint event will be censored at the time of their last follow-up visit. The Cox model will include treatment and the stratification factors for randomization. The effect of ASP0113 compared with placebo will be estimated as 1- hazard ratio, which is estimated from the Cox model. The corresponding 95% CI for the effect will be presented.

Efficacy analyses will be limited to the data obtained from time of first dose of study drug through the 1 year post-transplant follow-up period. Data collected after the 1 year post-transplant follow-up period for the long-term follow-up will not be included for the primary analysis of the primary endpoint.

7.4.1.2 Secondary Analysis for the Primary Endpoint

An analysis of the primary endpoint will be repeated on the PPS. The method used for this analysis will be identical to the primary analysis described in [Section 7.4.1.1]. Additional sensitivity analyses for the primary endpoint will be conducted using only the subjects having complete follow-up through 1 year post-transplant and using alternative imputations of outcomes.

7.4.1.3 Subgroup Analysis

The impact on treatment effect will be assessed by a Breslow-Day test for each of the following factors: age (less than study median, greater than or equal to study median), race (white, non-white), gender (male, female), subjects who had ATG as part of their conditioning regimen vs. those who did not, and myeloablative vs. non-myeloablative conditioning regimen. If the P value of Breslow-Day test is ≤ 0.10 the treatment effect will be estimated for each strata of the subgroup.

If enough donors are enrolled in the optional donor substudy then subgroup analyses by donor T-cell mediated immunity will also be performed.

7.4.2 Analysis of Secondary Variables

7.4.2.1 Key Secondary Variables

A Cox proportional hazards model will be used to assess the effect on the time-to-first protocol-defined CMV viremia through 1 year post-transplant. Protocol-defined CMV viremia is defined as a CMV plasma load ≥1000 IU/mL as assessed by the central laboratory.

The Cox model will contain the same terms as the 1 described above. Vaccine efficacy will be estimated as:

VE = 1 – hazard ratio, and 95 % CI will be provided.

To compare the treatment effect on protocol-defined CMV viremia between ASP0113 and placebo, the following null hypothesis will be constructed:

$$H_0: VE \leq 20\%.$$

The accompanying alternative hypothesis is:

$$H_1: VE > 20\%.$$

The P value for the above hypothesis tests will use a Wald statistic using the Cox model parameter estimate for the treatment effect, θ_t:

$$Z = (\theta_t - \ln(1\text{-}20\%))/S.E.(\theta_t)$$

The 95% CI for VE will be calculated by taking 1 minus the 95% CI for the hazard ratio. Alternative hypothesis will be accepted if the 95% CI for VE excludes 20%.

The hazard rate of first adjudicated CMV-specific AVT through 1 year post-transplant and for first CMV-specific AVT for protocol defined viremia will be analyzed similar to the analysis for CMV viremia. These analyses will test for simple superiority (0% efficacy margin) rather than use a 20% efficacy margin.

7.4.2.2 Other Secondary Variable

The endpoint of time to first CMV-specific AVT for protocol-defined CMV viremia or CMV EOD through 1 year post-transplant will be analyzed using a Cox proportional hazards model which includes effects for treatment, donor CMV serostatus, and donor-recipient relatedness.

7.4.3 Analysis of Other Variables

The maximum grade of aGVHD, maximum cGVHD global score, number of episodes of protocol-defined CMV viremia, and duration of hospitalizations will each be tested using a Kruskal-Wallis test.

Incidence of overall mortality, CMV EOD, grade 3-4 aGVHD, severe cGVHD, grade 3 infections other than CMV, relapse mortality, non-relapse mortality, graft rejection, failure to engraft, and incidence of relapse of primary disease requiring therapy will each be tested using a stratified analysis of the binary response using a CMH test to account for the stratification variables used as part of the randomization. Subjects who discontinue the study without experiencing the event considered in the analysis will be considered as having the event for the statistical analysis.

Number of episodes of protocol-defined CMV viremia [CMV plasma viral load ≥1000 IU/mL by the Abbott RealTi*m*e CMV assay as assessed by the central laboratory] will be compared between treatments using the stratum-adjusted Kruskal-Wallis test, adjusting for donor-recipient relatedness and donor CMV status. To estimate the association between CMV viremia and mortality, a multivariable Cox proportional hazards model will be constructed in a time-dependent manner. The model will be adjusted for the factors used to stratify the randomization. CMV viremia will be treated as a time-dependent variable, starting at the time of the first occurrence of protocol-defined CMV viremia.

The maximum grade of aGVHD, maximum cGVHD global score, and duration of hospitalizations will each be tested using a stratum-adjusted Kruskal-Wallis test adjusting for donor-recipient relatedness and donor CMV serostatus.

The treatment effect on time to engraftment and time to platelet recovery will each be analyzed in a Cox proportional hazards model including the factors used to stratify the randomization.

Immunogenicity analysis will be conducted using the IAS. Descriptive statistics on the raw and log transformed results will be presented by treatment group and collection time point. The Wilcoxon rank sum test will be used to compare treatment groups at each of the time points for immunogenicity assessment for the following:

- Antibody response to gB antigen
- T cell response to viral protein pp65

The EQ-5D response for each domain will be summarized by treatment arm at each visit. The total FACT-BMT and the corresponding change from baseline will be summarized by treatment arm at each visit and at final visit. The productivity outcomes from the WPAI will be summarized by treatment arm at each visit and at final visit. Treatments will be compared with respect to these scales using analysis of covariance of change from baseline to final visit with treatment, donor-recipient relatedness, and donor CMV serostatus as factors and baseline value as a covariate.

7.5 Analysis of Safety

The SAF will consist of all randomized subjects who have at least 1 study drug injection. This analysis set will be used for all summaries and analysis of AEs, reactogenicity, clinical laboratory data, PEs, vital signs, and subject survival through 1 year post first study drug injection. All recorded AEs will be listed, including duration, outcome, toxicity grade, and association with use of study drug. In addition, the toxicity of antiviral therapy will be identified from its association with those products.

All AEs and SAEs will be collected from signing of the ICF through 30 days post last dose of study drug. All events requiring adjudication, Grade 3 and higher AEs and SAEs will be collected from 31 days post last dose of drug through 1 year post-transplant. AEs and SAEs are to be reported as a change in medical status or medical history from time of signing of informed consent through first dose of study drug. In this study, the day 0 HCT is not considered an AE.

TEAEs will be defined as those that occurred after the first dose of study drug and within 30 days of the last dose of study drug.

The coding dictionary for this study will be MedDRA. It will be used to summarize AEs by system organ class and preferred term.

Tables will include the following details:

- Number of TEAEs
- Number and percentage of subjects with TEAEs
- Number and percentage of subjects with drug related TEAEs
- Number of serious TEAEs

- Number and percentage of subjects with serious TEAEs
- Number and percentage of subjects with TEAEs by NCI-CTCAE Version 4.03 scale. AEs will be summarized by their worst CTCAE grade
- TEAEs that are deemed by the Investigator as local reactogenicity will be graded and summarized by their worst grade. The grading will be done using both the NCI-CTCAE Version 4.03 grading scale and in accordance with the FDA's Guidance for Industry: Toxicity Grading Scale for Healthy Adult and Adolescent Volunteers Enrolled in Preventive Vaccine Clinical Trials [Appendix 7]
- Number of deaths

The number and percentage of subjects with TEAEs, as classified by system organ class and patient, as well as the number and percentage of subjects with at least 1 TEAE, will be summarized for each treatment group. Similar summaries will also be provided for drug related TEAEs, serious TEAEs, and TEAEs that lead to study discontinuation. A drug-related TEAE is defined as any TEAE with at least possible relationship to study treatment as assessed by the Investigator or with missing assessment of the causal relationship.

Additionally, the following tables will be presented for AEs occurring any time after first dose of study drug during the primary study period:

- Number and percentage of subjects with drug related AEs
- Number of serious AEs
- Number and percentage of subjects with serious AEs
- Number and percentage of subjects with AEs by CTCAE scale. AEs will be summarized by their worst CTCAE grade
- AEs that are deemed by the Investigator as local reactogenicity summarized by their worst grade
- Number of deaths

Lastly, the following tables will be presented for the Long-term Follow-up Period overall and by target month:

- Number of deaths
- Number of new or recurrent cancers
- Number and percentage of subjects with new or recurrent cancers
- Number of infections requiring hospitalization or resulting in death
- Number and percentage of subjects with infections requiring hospitalization or resulting in death
- Number of subjects with local erythema or induration at site of injection of study drug
- Number and percentage of subjects with local erythema or induration at site of injection of study drug

The number and percentage of subjects completing each long-term follow-up period assessment will be presented by target month and treatment arm.

7.6 Analysis of Pharmacokinetics

Pharmacokinetics will not be analyzed in this study.

7.7 Analysis of Pharmacogenomics

Samples will be collected and stored as described in [Appendix 19], Pharmacogenomics Sub-study, for potential retrospective analysis.

7.8 Protocol Deviations and Other Analysis

Protocol deviations as defined in [Section 8.1.6] will be summarized for all randomized subjects by treatment group and total as well as by site. A data listing will be provided by site and subject.

The protocol deviation criteria will be uniquely identified in the summary table and listing. The unique identifiers will be as follows:

PD1- Entered into the study even though they did not satisfy entry criteria,

PD2- Developed withdrawal criteria during the study and was not withdrawn,

PD3- Received wrong treatment or incorrect dose,

PD4- Received excluded concomitant treatment.

7.9 Interim Analyses and Early Discontinuation of the Clinical Study

Futility analysis will be conducted during this study. Futility will be monitored based on the effect of ASP0113 on viremia (defined as a CMV plasma viral load ≥1000 IU/mL as assessed by the central laboratory). It is believed that viremia is a surrogate of treatment effect.

Futility analysis will be performed when the first 100 subjects have been randomized, and every 3 months thereafter until the total enrollment reaches 400 subjects. At each futility analysis, a Bayesian posterior probability that the hazard ratio for viremia of the ASP0113 treatment group to the placebo treatment group will be less than 0.75 will be calculated. The study may stop for futility if this posterior probability is less than 0.15.

Futility analysis will be carried out by an external data analysis group. Sponsor personnel will not have access to unblinded interim analysis data during the study unless the futility criterion is met and the Sponsor needs to make a decision on discontinuing the trial.

7.9.1 DMC

A DMC, independent of the Sponsor and investigators, will be established to review the safety data throughout the study. The independent DMC statistician will have access to the randomization schedule. Unblinded deliverables for use by the DMC will be created by an independent data analysis center. The Sponsor will not have access to the unblinded tables and listings created for the DMC. All subjects who have been enrolled will be included in the DMC reviews and all data submitted to the DMC will remain blinded to Astellas personnel involved in the study.

Further details of the futility assessments and DMC safety review are available in the SAP, the Interim Statistical Analysis Plan (IAP) and DMC charter.

7.10 Handling of Missing Data, Outliers, Visit Windows, and Other Information

In the analysis of the primary endpoint, subjects with unknown survival status will be assumed to have had the primary endpoint. Sensitivity analysis excluding those subjects from the denominator will be conducted. Other imputations of missing data will be considered based on the nature and quantity of missing data with appropriate statistical methods which will be fully specified prior to unblinding. Assignments of observations to visits for analyses by scheduled visit will be based on dates relative to date of transplantation. The details will be described in the SAP.

8 OPERATIONAL AND ADMINISTRATIVE CONSIDERATIONS

8.1 Procedure for Clinical Study Quality Control

8.1.1 Data Collection

The Investigator or site designee is responsible to ensure that all data in the eCRFs and queries are accurate and complete and that all entries are verifiable with source documents. These documents are to be appropriately maintained by the site.

The Investigator or designee will enter data collected using an Electronic Data Capture system or data will be electronically transferred by an external vendor to Astellas' clinical database (e.g., central lab data). Data will be collected for all subjects who sign an ICF and are screened for the study, whether or not they are subsequently randomized. For screening failures, minimal demographic data and reason for screening failure will be collected.

The monitor should verify the data in the eCRFs with source documents and confirm that there are no inconsistencies between them.

For screen failures the minimum demographic data (i.e., sex, age, informed consent date) and reason for screen failure will be collected in the eCRF and the screen failure log if applicable.

8.1.2 Specification of Source Documents

Source data must be available at the site to document the existence of the study subjects and substantiate the integrity of study data collected. Source data must include the original documents relating to the study, as well as the medical treatment and medical history of the subject.

The following information should be included in the source medical records:

- Demographic data (date of birth, sex, race, ethnicity (USA only), height and body weight)
- Inclusion and exclusion criteria details
- Participation in study and signed and dated ICFs
- Visit dates

- Medical history and PE details
- Key efficacy and safety data as specified in the protocol
- AEs and concomitant medication
- Results of relevant examinations (e.g., ECG tracings, X-ray films etc.)
- Laboratory printouts (if applicable)
- Kit number assignment and dispensing and return of study drug details
- Reason for premature discontinuation (if applicable)
- Subject number

8.1.3 Clinical Study Monitoring

The Sponsor or delegated contract research organization (CRO) is responsible for monitoring the clinical study to ensure that subject's human rights, safety, and well-being are protected, that the study is properly conducted in adherence to the current protocol and GCP, and study data reported by the Investigator/sub-Investigator are accurate and complete and that they are verifiable with study-related records such as source documents. The Sponsor is responsible for assigning study monitor(s) to this study for proper monitoring. They will monitor the study in accordance with planned monitoring procedures.

8.1.4 Direct Access to Source Data/Documents

The Investigator and the study site must accept monitoring and auditing by the Sponsor or delegated CRO as well as inspections from the IRB/IEC and relevant regulatory authorities. In these instances, they must provide all study-related records, such as source documents (refer to [Section 8.1.2]) when they are requested by the Sponsor monitors and auditors, the IRB/IEC, or regulatory authorities. The confidentiality of the subject's identities shall be well protected and maintained in compliance with local and national regulations when the source documents are subject to direct access.

8.1.5 Data Management

Data management will be coordinated by the Global Data Science Department of the Sponsor or designee in accordance with the SOPs for data management. All study specific processes and definitions will be documented by Data Management in the Data Management Plan for the study. The eCRF completion and correction processes will be referenced in the eCRF instructions. Coding of AEs and medical terms will be performed using MedDRA and World Health Organization dictionaries.

8.1.6 Protocol Deviations

A protocol deviation is generally an unplanned excursion from the protocol that is not implemented or intended as a systematic change. The Investigator is responsible for ensuring the study is conducted in accordance with the procedures and evaluations described in this protocol and must protect the rights, safety, and welfare of subjects. The Investigator should not implement any deviation from, or changes of, the protocol, unless it is necessary to eliminate an immediate hazard to trial subjects.

A protocol waiver is a documented prospective approval of a request from an Investigator to deviate from the protocol. Protocol waivers are strictly prohibited within Astellas.

For the purposes of this protocol, deviations requiring notification to Sponsor are defined as any subject who:

- Entered into the study even though they did not satisfy entry criteria.
- Developed withdrawal criteria during the study and was not withdrawn.
- Received wrong treatment or incorrect dose.
- Received excluded concomitant treatment.

When a deviation from the protocol is identified for an individual subject, the Investigator or designee must ensure the Sponsor is notified. The Sponsor will follow-up with the Investigator, as applicable, to assess the deviation and the possible impact to the safety and/or efficacy of the subject to determine subject continuation in the study.

If a deviation impacts the safety of a subject, the Investigator must contact Sponsor immediately.

The Investigator will also assure that deviations meeting IRB/IEC and applicable regulatory authorities criteria are documented and communicated appropriately. All documentation and communications to the IRB/IEC and applicable regulatory authorities will be provided to Astellas and maintained within the Trial Master File.

NOTE: Other deviations outside of the categories defined above that are required to be reported by the IRB/IEC in accordance with local requirements will be reported, as applicable.

8.1.7 End of Trial in All Participating Countries

The end of trial in all participating countries is defined as the last subject’s last visit.

8.2 Ethics and Protection of Subject Confidentiality

8.2.1 IRB / IEC / CA

GCP requires that the clinical protocol, any protocol amendments, the IB, the informed consent and all other forms of subject information related to the study (e.g., advertisements used to recruit subjects) and any other necessary documents be reviewed by an IEC/IRB. The IEC/IRB will review the ethical, scientific and medical appropriateness of the study before it is conducted. IEC/IRB approval of the protocol, informed consent and subject information and/or advertising, as relevant, will be obtained prior to the authorization of drug shipment to a study site.

Any substantial amendments to the protocol will require IEC/IRB approval prior to implementation of the changes made to the study design at the site. The Investigator will be required to submit, maintain and archive study essential documents according to ICH GCP.

Any SAEs that meet reporting criteria, as dictated by local regulations, will be reported to both responsible Ethics Committees and Regulatory Agencies, as required. During the

conduct of the study, the Investigator should promptly provide written reports (e.g., ICH Expedited Reports, and any additional reports required by local regulations) to the IEC/IRB, providing notice of any changes that affect the conduct of the study and/or increase the risk to subjects. Written documentation of the submission to the IEC/IRB should also be provided to the Sponsor.

If required by local regulations, the Investigator shall make accurate and adequate written progress reports to the IEC/IRB at appropriate intervals, not exceeding 1 year. The Investigator shall make an accurate and adequate final report to the IRB/IEC within 90 days after the close-out visit for Astellas sponsored studies, within 1 year after last subject out or termination of the study.

8.2.2 Ethical Conduct of the Study

The study will be conducted in accordance with the protocol, ICH guidelines, applicable regulations and guidelines governing clinical study conduct and the ethical principles that have their origin in the Declaration of Helsinki.

8.2.3 Informed Consent of Subjects

8.2.3.1 Subject Information and Consent

The Investigator or his/her representative will explain the nature of the study to the subject or his/her guardian or legal representative, and answer all questions regarding this study. Prior to any study-related screening procedures being performed on the subject, the informed consent statement will be reviewed and signed (specific to sites in Japan place a personal seal) and dated by the subject or his/her guardian or legal representative, the person who administered the informed consent and any other signatories according to local requirements. A copy of the signed (specific to sites in Japan or sealed) informed consent form will be given to the subject and the original will be placed in the subject’s medical record. An entry must also be made in the subject’s dated source documents to confirm that informed consent was obtained prior to any study-related procedures and that the subject received a signed copy.

The signed/sealed consent forms will be retained by the Investigator and made available (for review only) to the study monitor, auditor, regulatory authorities and other applicable individuals upon request.

8.2.3.2 Supply of New and Important Information Influencing the Subject’s Consent and Revision of the Written Information

1. The Investigator/sub-Investigator will immediately inform the subject orally whenever new information becomes available that may be relevant to the subject's consent or may influence the subject's willingness to continue participation in the study (e.g., report of serious ADRs). The communication must be documented in the subject's medical records, and it should be confirmed whether the subject is willing to remain in the study or not.

2. The Investigator must update the ICF and the updated ICF is submitted for approval to the IRB/IEC. The Investigator or his/her representative must obtain written informed consent from the subject on all updated ICFs throughout their participation in the study. The Investigator or his/her designee must reconsent subjects with the updated ICF even if relevant information was provided orally. The subject and the Investigator or his/her representative who obtained the written informed consent should sign and date the ICF (specific to sites in Japan: place a personal seal). A copy of the signed (specific to sites in Japan: or sealed) ICF will be given to the subject and the original will be placed in the subject's medical record. An entry must be made in the subject's records documenting the reconsent process.

8.2.4 Subject Confidentiality

Individual subject medical information obtained as a result of this study is considered confidential and disclosure to third parties is prohibited. Such medical information may be given only after approval of the subject to the subject's physician or to other appropriate medical personnel responsible for the subject's well-being.

The Sponsor shall not disclose any confidential information on subjects obtained during the performance of their duties in the clinical study without justifiable reasons.

The Sponsor affirms the subject's right to protection against invasion of privacy. Only a subject identification number and/or initials (if not prohibited by local regulations) will identify subject data retrieved by the Sponsor. However, the Sponsor requires the Investigator to permit the Sponsor, Sponsor's representative(s), the IRB/IEC and when necessary, representatives of the regulatory health authorities to review and/or to copy any medical records relevant to the study.

The Sponsor will ensure that the use and disclosure of protected health information (PHI) obtained during a research study complies with the federal and/or regional legislation related to the privacy and protection of personal information (e.g., HIPAA for U.S. sites).

For US sites, the HIPAA Privacy Rule provides federal protection for the privacy of PHI by implementing standards to protect and guard against the misuse of individually identifiable health information of subjects participating in Sponsored clinical trials. "Authorization" is required from each research subject, i.e. specific permission granted by an individual to a covered entity for the use or disclosure of an individual's PHI. A valid authorization must meet the implementation specifications under the HIPAA Privacy Rule. Authorization may be combined into the Informed Consent document (approved by the IRB/IEC) or it may be a separate document [approved by the IRB/IEC or designated privacy board (PB)] or provided by the Investigator or Sponsor (without IRB/IEC or PB approval). It is the responsibility of the Investigator and institution to obtain such waiver/authorization in writing from the appropriate individual.

For sites in Japan:

All individuals and organizations involved in the study must pay very careful attention to protect subjects' privacy with appropriate measures, for example, by prohibiting the use of any private information that may identify a subject (e.g., name or address). These details shall be processed in accordance with the applicable local and regional laws.

Even though any individuals involved in the study, including the Sponsor monitors and auditors, may get to know matters related to subjects' privacy due to direct access to source documents, or from other sources, they may not leak the content to third parties.

8.3 Administrative Matters

8.3.1 Arrangement for Use of Information and Publication of the Clinical Study

Information concerning the study drug, patent applications, processes, unpublished scientific data, the IB and other pertinent information is confidential and remains the property of the Sponsor. Details should be disclosed only to the persons involved in the approval or conduct of the study. The Investigator may use this information for the purpose of the study only. It is understood by the Investigator that the Sponsor will use the information obtained during the clinical study in connection with the development of the drug and therefore may disclose it as required to other clinical investigators or to regulatory agencies. In order to allow for the use of the information derived from this clinical study, the Investigator understands that he/she has an obligation to provide the Sponsor with all data obtained during the study.

The study will be considered for publication or presentation at (scientific) symposia and congresses. The Investigator will be entitled to publish or disclose the data generated at their respective study site only after submission to the Sponsor all transcripts, texts of presentations, and abstracts related to the study at least (for sites in Japan: 90 days prior to the intended submission for publication or any other disclosure studies, or) 30 days prior to the intended submission for publication or any other disclosure studies for APGD-Sponsored studies. This is necessary to confirm whether any inventive knowledge should be protected by a patent or not and to prepare and file a patent application accordingly. In addition this is in no way intended to restrict publication of facts or opinions formulated by the Investigator. The Sponsor will inform the Investigator in writing of any objection or question arising within 30 days of receipt of the proposed publication material.

For sites in Japan: After agreement between investigator(s) and Sponsor, the manuscript is free for publication.

8.3.2 Documents and Records Related to the Clinical Study

The Sponsor will provide the Investigator and/or institution with the following:

- Study protocol (and amendments, as applicable)
- IB (and amendments, as applicable)
- CRFs and SAE Report Worksheet
- Study drug with all necessary documentation

- Study contract
- For sites in Japan: JUTOKUNA YUUGAIJISHOU HOUKOKUSHO

In order to start the study, the Investigator and/or study site is required to provide the following documentation to the Sponsor:

- Financial disclosure in compliance with US federal regulation 21CFR Part 54.
- Signed Investigator's Statement in this protocol.
- Executed Research Agreement.
- Signed and dated FDA form 1572.
- Copy of the approved ICF and separate authorization form, if appropriate.
- IEC/IRB approval of the protocol, protocol amendments (if applicable) and ICF (and separate authorization form, if appropriate), stating clearly the Sponsor's name, study number and study drug, including a membership list with names and qualifications.
- Current Curricula Vitae of all investigators (signed and dated).
- Laboratory normal reference ranges (if applicable, signed and dated by the responsible laboratory employee).
- Medical/Laboratory/Technical procedures/tests certifications or accreditations or established quality control or other validation, where required.
- List of sub-Investigators and collaborators (Japan sites only)
- Instruction and decision of the head of the study center (Japan sites only)

At the end of the study, the Sponsor is responsible for the following:

- Collection of and/or destruction of unused CRFs and other study documentation.
- Ensuring destruction of unused study drug

The Investigator will archive all study data (e.g., Subject Identification Code List, source data, CRFs, and Investigator's File) and relevant correspondence. These documents are to be kept on file for the appropriate term determined by local regulation (for US sites, 2 years after approval of the NDA or discontinuation of the IND).

For countries except Japan: The Sponsor will notify the site/Investigator if the NDA/MAA/J-NDA is approved or if the IND/IMPD/CHIKEN TODOKE is discontinued. The Investigator agrees to obtain the Sponsor's agreement prior to disposal, moving, or transferring of any study-related records. The Sponsor will archive and retain all documents pertaining to the study according to local regulations.

Data generated by the methods described in the protocol will be recorded in the subjects' medical records and/or study progress notes. All data will be entered on eCRFs supplied for each subject.

For sites in Japan only:

The records to be retained at the study centers are the ones listed as essential documents in GCP. These records shall be retained by the head of the study center or the record keeper designated by the head until notice issued by the Sponsor on completion of the retention period

is received. These documents are also subject to direct access and should be provided upon request from the Sponsor or regulatory authorities.

The head of the study center will retain the essential documents that should be stored at the study center in an appropriate manner according to the rules of the study center concerned until the date defined in 1. or 2. below, whichever comes later.

1. Approval date of marketing of the test drug (if development of the drug is discontinued, until 3 years after the decision to discontinue development is notified)
2. Until 3 years after discontinuation or termination of the study.

8.3.3 Protocol Amendment and/or Revision

Any changes to the study that arise after approval of the protocol must be documented as protocol amendments: substantial amendments and/or non-substantial amendments. Depending on the nature of the amendment, either IRB/IEC Competent Authority approval or notification is required. The changes will become effective only after the approval of the Sponsor, the Investigator, the regulatory authority (except sites in Japan), and the IRB/IEC (if applicable). (Unique to sites in Japan: followed by the approval of the head of the study site).

Amendments to this protocol must be signed by the Sponsor and the Investigator. Written verification of IRB/IEC approval will be obtained before any amendment is implemented which affects subject safety or the evaluation of safety, and/or efficacy. Modifications to the protocol that are administrative in nature do not require IRB/IEC approval, but will be submitted to the IRB/IEC for their information, if required by local regulations.

If there are changes to the ICF, written verification of IRB/IEC approval must be forwarded to the Sponsor. An approved copy of the new ICF must also be forwarded to the Sponsor.

8.3.4 Insurance of Subjects and Others (FOR JAPAN SITES ONLY)

If a subject suffers any study-related injury, the Sponsor will compensate appropriately according to the severity and duration of the damage. However, if it was caused intentionally or was due to gross negligence by the study center, the Sponsor will consult with the study center about handling the injury, based on the agreed study contract.

Compensation for study-related injury is provided by the following procedures:

1. If a subject incurs an injury as a result of participation in the clinical study, the study center should provide medical treatment and other necessary measures. The Sponsor should be notified of the injury.
2. When the subject claims compensation from the study center for the above study-related injury, or such compensation may be claimed, the study center should immediately communicate the fact to the Sponsor. Both parties should work together towards compensation settlement.
3. The Sponsor shall pay compensation or indemnification and bear expenses necessary for the settlement as provided in the clinical contract.

4. The Sponsor shall make an arranging for insurance and take measures necessary to ensure the compensation or indemnification mentioned above.

8.3.5 Signatory Investigator for Clinical Study Report

ICH E3 guidelines recommend and EU Directive 2001/83/EC requires that a final study report which forms part of a marketing authorization application be signed by the representative for the Coordinating Investigator(s) or the Principal Investigator(s). The representative for the Coordinating Investigator(s) or Principal Investigator(s) will have the responsibility to review the final study results to confirm to the best of his/her knowledge it accurately describes the conduct and results of the study. The representative for Coordinating Investigator(s) or Principal Investigator(s) will be selected from the participating Investigators by the Sponsor prior to database lock.

9 QUALITY ASSURANCE

The Sponsor is implementing and maintaining quality assurance and quality control systems with written SOPs to ensure that trials are conducted and data are generated, documented (record), and reported in compliance with the protocol, GCP, and applicable regulatory requirement(s).

The Sponsor or Sponsor's designee may arrange to inspect/audit the clinical study at any or all investigational sites. The auditor is independent from the clinical monitoring and project management team at the Sponsor. The audit may include on-site review of regulatory documents, case report forms, and source documents. Direct access to these documents will be required by the auditors.

10 STUDY ORGANIZATION

10.1 Data Monitoring Committee (DMC)

A DMC external from the Sponsor will be chartered to oversee safety issues and to assess the quality of the trial conduct. The DMC will consist of independent reviewers who are not directly involved in the conduct of the study. For additional details of the DMC refer to [Section 7.9.1] and the study DMC Charter.

10.2 Adjudication Committee

An AC external from the Sponsor will be charted to adjudicate all subjects for cases of CMV EOD, initiation of CMV-specific AVT, and cause of death. The AC will consist of a multidisciplinary group of independent clinician-scientists who are not directly involved in the conduct of the study. For details of the committee refer to the study AC Charter.

10.3 Other Study Organization

10.3.1 Home Healthcare

For sites in applicable regions, laboratory draws for central laboratory testing and collection of subject local reactogenicity diaries may be performed by qualified staff from the designated home healthcare agencies for the study.

11 REFERENCES

Available upon request

Akpek G, Lee SJ, Flowers ME, Pavletic SZ, Arora M, Lee S, et al. Performance of a new clinical grading system for chronic graft-versus-host disease: a multicenter study. Blood. 2003;102:802-9.

Boeckh M, Ljungman P. How we treat cytomegalovirus in hematopoietic cell transplant recipients. Blood. 2009; 113(23):5711-19.

Boeckh M and Nichols WG. The impact of cytomegalovirus serostatus of donor and recipient before hematopoietic stem cell transplantation in the era of antiviral prophylaxis and preemptive therapy. Blood. 2004;15:103:2003-8.

Britt WJ, Vugler L, Butfiloski EJ, Stephens EB. Cell surface expression of human cytomegalovirus (HCMV) gp55-116 (gB): use of HCMV-recombinant vaccinia virus-infected cells in analysis of the human neutralizing antibody response. J Virol. 1990; 64:1079-85.

Broers A E, van der Holt R, van Esser Joost W J, Gratama J-W, Henzen-Logmans S, Kuenen-Boumeester V, et al. Increased transplant-related morbidity and mortality in CMV-seropositive patients despite highly effective prevention of CMV disease after allogeneic T-cell–depleted stem cell transplantation. Blood. 2000;95:2240-5.

Cantoni N, Hirsch H, Khanna N, Gerull S, Buser A, Bucher C, et al. Evidence for a bidirectional relationship between cytomegalovirus replication and acute graft-versus-host disease. Biol Blood Marrow Transplant. 2010;16:1309-14.

Castro-Malaspina H, Harris RE, Gajewski J, Ramsay N, Collins R, Dharan B, et al. Unrelated donor marrow transplantation for myelodysplastic syndromes: outcome analysis in 510 transplants facilitated by the National Marrow Donor Program. Blood. 2002;99:1943-51.

Cheson BD, Bennett JM, Kopecky KJ, Buchner T, Willman CL, Estey EH, et al. Revised recommendations of the international working group for diagnosis, standardization of response criteria, treatment outcomes, and reporting standards for therapeutic trials in acute myeloid leukemia. J Clin Oncol. 2003;21(24):4642-9.

Cornelissen JJ, Carston M, Kollman C, King R, Dekker AW, Löwenberg B, et al. Unrelated marrow transplantation for adult patients with poor-risk acute lymphoblastic leukemia: strong graft-versus-leukemia effect and risk factors determining outcome. Blood. 2001;97:1572-7.

Craddock C, Szydlo RM, Dazzi F, Olavarria E, Cwynarski K, Young A et al. Cytomegalovirus seropositivity adversely influences outcome after T-depleted unrelated donor transplant in patients with chronic myeloid leukemia: the case for tailored graft-versus-host disease prophylaxis. Br J Haematol. 2001;112:228-36.

Craddock C, Labopin M, Pillai S, Finke J, Bunjes D, Greinix H, et al. Factors predicting outcome after unrelated donor stem cell transplantation in primary refractory acute myeloid leukemia. Leukemia. 2011;25:808-13.

Doney K, Hagglund H, Leisenring W, Chauncey T, Appelbaum FR, and Storb R. Predictive factors for outcome of allogeneic hematopoietic cell transplantation for adult acute lymphoblastic leukemia. Biol. Blood Marrow Transp. 2003;9:472-81.

Filipovich AH, Weisdorf D, Pavletic S, Socie G, Wingard JR, Lee SJ, et al. National institutes of health consensus development project on criteria for clinical trials in chronic graft-versus-host disease: 1. diagnosis and staging working group report. Biology of Blood and Marrow Transplantation 2005;11:945-55.

Glucksberg H, Storb R, Fefer A, Buckner CD, Neiman PE, Clift RA, et al. Clinical manifestations of graft-versus-host disease in human recipients of marrow from HL-A-matched sibling donors. Transplantation. 1974;18:295-304.

Guidance for Industry titled "Drug-Induced Liver Injury: Premarketing Clinical Evaluation" issued by FDA on July 2009.

Guidance for Industry titled "Toxicity Grading Scale for Healthy Adult and Adolescent Volunteers Enrolled in Preventive Vaccine Clinical Trials" issued by FDA on September 2007.

Gyulai Z, Endresz V, Burian K, Pincus S, Toldy J, Cox WI, et al. Cytotoxic T lymphocyte (CTL) responses to human cytomegalovirus pp65, IE1-Exon4, gB, pp150, and pp28 in healthy individuals: reevaluation of prevalence of IE1-specific CTLs. J Infect Dis. 2000;181:1537-46.

Karnofsky DA, Burchenal JH. The clinical evaluation of chemotherapeutic agents in cancer, in Macleod CM (ed): Evaluation of Chemotherapeutic Agents. New York, Columbia University Press, 1949;199-205.

Kern F, Bunde T, Faulhaber N, Kiecker F, Khatamzas E, Rudawski IM, et al. Cytomegalovirus (CMV) phosphoprotein 65 makes a large contribution to shaping the T cell repertoire in CMV-exposed individuals. J Infect Dis. 2002;185:1709-16.

Kharfan-Dabaja MA, Boeckh M, Wilck MB, Langston AA, Chu AH, Wloch MK, et al. A novel cytomegalovirus DNA vaccine in allogeneic haemopoietic stem-cell transplantation: a randomized, double-blind, placebo-controlled phase 2 trial. The Lancet Infectious Diseases. 2012;12:290-9. Corrections in 2013;13:12.

Kroger N, Zabelina T, Kruger W, Renges H, Stute N, Schrum J et al. Patient cytomegalovirus seropositivity with or without reactivation is the most important prognostic factor for survival and treatment-related mortality in stem cell transplantation from unrelated donors using pretransplant in vivo T-cell depletion with anti-thmocyte globulin. Br J Haematol. 2001;113:1060-71.

Kumar D, Humar A. The AST handbook of transplant infections. 2011.

Larsson K, Aschan J, Remberger M, Ringden O, Winiarski J, Ljungman P. Reduced risk for extensive chronic graft-versus-host disease in patients receiving transplants with human leukocyte antigen-identical sibling donors given polymerase chain reaction-based preemptive therapy against cytomegalovirus. Transplantation. 2004;77:526-31.

Ljungman P, Griffiths P, Paya C. Definitions of cytomegalovirus infection and disease in transplant recipients. Clinical Infectious Diseases. 2002;34:1094-7.

McGlave PB, Shu XO, Wen W, Anasetti C, Nademanee A, Champlin R, et al. Unrelated donor marrow transplantation for chronic myelogenous leukemia: 9 years' experience of the national marrow donor program. Blood. 2000;95:2219-25.

Mocarski ES, Jr. Immunomodulation by cytomegaloviruses: manipulative strategies beyond evasion. Trends Microbiol. 2002;10:332-9.

Nichols WG, Corey L, Gooley T, Davis C, Boeckh M. High risk of death due to bacterial and fungal infection among cytomegalovirus (CMV)-seronegative recipients of stem cell transplants from seropositive donors: evidence for indirect effects of primary CMV infection. J Infect Dis. 2002;185:273-82.

Pasquini MC, Wang Z. Current uses and outcome of hematopoietic stem cell transplantation: CIBMTR Summary Slides, 2009.

Passamonti F, Cervantes F, Vannucchi AM, Morra E, Rumi E, Pereira A, et. al. A dynamic prognostic model to predict survival in primary myelofibrosis: a study by the IWG-MRT (International Working Group for Myeloproliferative Neoplasms Research and Treatment). Blood. 2010;115:1703-8.

Przepiorka D, Weisdorf D, Martin P, Klingemann H-G, Beatty P, Hows J and Thomas ED. Consensus conference on acute GVHD grading. BMT. 1995;15:825-28.

Scott BL, Gooley TA, Sorror ML, Rezvani AR, Linenberger ML, Grim J, et al. The dynamic international prognostic scoring system for myelofibrosis predicts outcomes after hematopoietic cell transplantation. Blood. 2012;119:2657-64.

Sorror ML, Maris MB, Storb R, Baron F, Sandmaier MB, Maloney DG, et al. Hematopoietic cell transplantation (HCT)-specific comorbidity index: a new tool for risk assessment before allogeneic HCT. Blood. 2005;106:2912-9.

Sorror, ML. How I assess comorbidities before hematopoietic cell transplantation. Blood. 2013;121:2854-63.

Srinivasan A, Wang C, Srivastava DK, Burnette K, Shenep JL, Leung W, et al. Timeline, epidemiology and risk factors for bacterial, fungal and viral infections in children and adolescents after allogeneic hematopoietic stem cell transplant. Bio Blood Marrow Transplant. 2012:1-8.

Tomblyn M, Chiller T, Einsele H, Gress R, Sepkowitz K, Storek J, et al. Guidelines for preventing infectious complications among hematopoietic cell transplantation recipients: A global perspective. Biol Blood Marrow Transplant. 2009;15:1143-238.

Wills MR, Carmichael AJ, Mynard K, Jin X, Weekes MP, Plachter B, et al. The human cytotoxic T-lymphocyte (CTL) response to cytomegalovirus is dominated by structural protein pp65: frequency, specificity, and T-cell receptor usage of pp65-specific CTL. J Virol. 1996;70:7569-79.

Wingard JR, Carter SL, Walsh TJ, Kurtzberg J, Small TN, Baden LR, et al. Randomized double-blind trial of fluconazole versus voriconazole for prevention of invasive fungal infection after allogeneic hematopoietic cell transplantation. Blood. 2010;116:5111-8.

Wloch MK, Smith LR, Boutsaboualoy S, Reyes L, Han C, Kehler J, et al. Safety and Immunogenicity of a Bivalent Cytomegalovirus DNA Vaccine in Healthy Adult Subjects. JID. 2008;197:1634-42.

Yakoub-Agha I, Mesnil F, Kuentz M, Boiron JM, Ifrah N, et al. Allogeneic marrow stem-cell transplantation from human leukocyte antigen-identical siblings versus human leukocyte antigen-allelic-matched unrelated donors (10/10) in patients with standard-risk hematologic malignancy: A prospective study from the French Society of Bone Marrow Transplantation and Cell Therapy. J Clin Onc. 2006;24:5695-702.

Company Reports:

ASP0113 Investigator's Brochure

12 APPENDICES

12.1 Appendix 1: Laboratory Tests

Central Laboratory Testing		
**Laboratory Test**	**Visit**	**Parameters to be Analyzed**
CMV Plasma Viral Load	Weekly (days 0-100)<br>Every other week (days 101-180)<br>Every 30 days (days 181-365)	• Cytomegalovirus (CMV) plasma viral load
Hematology	V2, V5-V12, including safety follow-up time points for doses 4 & 5	• Red blood cells<br>• White blood cells (total leukocytes)<br>• Hemoglobin<br>• Hematocrit<br>• Platelets (thrombocytes)<br>• Neutrophils (ANC)<br>• Eosinophils<br>• Basophils<br>• Lymphocytes<br>• Monocytes<br>• Blast cells
Biochemistry	V2, V5-V12, including safety follow-up time points for doses 4 & 5	• Sodium<br>• Potassium<br>• Calcium<br>• Chloride<br>• Glucose<br>• Creatinine<br>• Creatine phosphokinase (CPK)/Creatine kinase (CK)<br>• Total protein<br>• Albumin
Hepatic Profile	V2, V5-V12, including safety follow-up time points for doses 4 & 5	• Total bilirubin<br>• Direct bilirubin<br>• Alkaline Phosphatase<br>• Aspartate aminotransferase<br>• Alanine aminotransferase (ALT)
Immunogenicity	V2, V5, V8, V9, V11 & V14	• gB-specific antibody levels<br>• T-cell responses to phosphoprotein 65
Pharmacogenomics (PGx) – optional – saliva	V2	• Genomic analyses

12.1 Appendix 1: Laboratory Tests continued

Local Laboratory Testing		
**Laboratory Test**	**Visit**	**Parameters to be Analyzed**
CMV Screening	Screening (V1)	• CMV antibody assay
CMV Plasma Viral Load	As allowed per protocol	• CMV plasma viral load
Urine or Serum Pregnancy Test (all females of childbearing potential and all females who are of non-childbearing potential but are < 50 years of age and not documented to be surgically sterile)	V1, V2, V5, V7, V9, V11, V13 & V14	• Human Chorionic Gonadotropin (hCG)
Platelet Count	V2, V5, V7, V9 & V11	• Platelet count
Hematology	Screening (V1)	• Red blood cells<br>• White blood cells (total leukocytes)<br>• Hemoglobin<br>• Hematocrit<br>• Platelets (thrombocytes)<br>• ANC<br>• Eosinophils<br>• Basophils<br>• Lymphocytes<br>• Monocytes<br>• Blast cells
Biochemistry	Screening (V1)	• Sodium<br>• Potassium<br>• Calcium<br>• Chloride<br>• Glucose<br>• Creatinine<br>• CPK/CK<br>• Total protein<br>• Albumin
Hepatic Profile	Screening (V1)	• Total bilirubin<br>• Direct bilirubin<br>• Alkaline Phosphatase<br>• ALT<br>• AST

12.2 Appendix 2: Definition of CMV Disease

Disease type	Definition
CMV pneumonia	Signs and/or symptoms of pulmonary disease combined with the detection of cytomegalovirus (in BAL fluid*) or lung tissue samples performed by virus isolation, histopathologic testing, immunohistochemical analysis, or in situ hybridization<br>(The presence of fungal copathogens such as *Aspergillus* species, together with radiologic signs typical of *Aspergillus* pneumonia [e.g., a halo sign or a crescent sign] indicates fungal pneumonia rather than CMV pneumonia)
CMV gastrointestinal disease	Combination of clinical symptoms from the upper or lower gastrointestinal tract, findings of macroscopic mucosal lesions on endoscopy, and demonstration of CMV infection (by culture, histopathologic testing, immunohistochemical analysis, or in situ hybridizations) in a gastrointestinal tract biopsy specimen. If CMV is detected in normal mucosa near a lesion consistent with those typical of CMV infection, this can be accepted as CMV gastrointestinal disease.
CMV hepatitis	Elevated bilirubin and/or enzyme levels during liver function testing, absence of other documented cause of hepatitis, and detection of CMV infection (by culture, histopathologic testing, immunohistochemical analysis, or in situ hybridization) in a liver biopsy specimen. Other pathogens, such as hepatitis C virus, may be present without excluding the diagnosis of CMV hepatitis.
CMV CNS disease	CNS symptoms together with the detection of CMV in cerebrospinal fluid samples, by culture or PCR, or in brain biopsy specimens, by culture, histopathologic testing, immunohistochemical analysis, or in situ hybridization.
CMV retinitis	Lesions typical of CMV retinitis must be confirmed by an ophthalmologist or as confirmed by detection of CMV in vitreous fluid by culture or PCR.
CMV nephritis	CMV infection (by culture, immunohistochemical analysis, or in situ hybridization) together with the identification of histologic features of CMV infection in a kidney biopsy specimen obtained from a patient with renal dysfunction. Detection of CMV in the urine of a patient with kidney dysfunction does not fulfill the definition of CMV nephritis.
CMV cystitis	CMV infection (by culture, immunohistochemical analysis, or in situ hybridization) together with the identification of conventional histologic features of CMV infection in a bladder biopsy specimen obtained from a patient with cystitis. Detection of CMV in urine combined with identification of symptoms does not fulfill the definition of CMV cystitis.
CMV myocarditis	CMV infection (by culture, immunohistochemical analysis, or in situ hybridization) together with the identification of conventional histologic features of CMV infection in a heart biopsy specimen obtained from a patient with myocarditis.
CMV pancreatitis	CMV infection (by culture, immunohistochemical analysis, or in situ hybridization) together with the identification of conventional histologic features of CMV infection in a pancreatic biopsy specimen obtained from a patient with pancreatitis.
Other CMV disease categories	Definitions for additional disease categories include the presence of compatible symptoms and signs and documentation of CMV by biopsy with other relevant causes excluded.

BAL: bronchoalveolar lavage; CMV: cytomegalovirus; CNS: central nervous system; PCR: polymerase chain reaction

*Whenever a BAL is performed, fluid should be collected and a sample will be stored at the central laboratory.

Reference:

Ljungman P, Griffiths P, Paya C. Definitions of cytomegalovirus infection and disease in transplant recipients. Clinical Infectious Diseases. 2002;34:1094-7.

12.3 Appendix 3: Common Serious Adverse Events and Endpoint Events

The following is a list of serious adverse events (SAEs) that the Sponsor considers to be associated with the disease state being studied. **The list does NOT change your reporting obligations or prevent the need to report an adverse event meeting the definition of an SAE as detailed in** [**Section 5.5.3**] **Definition of Serious Adverse Event (SAE)**. The purpose of this list is to alert you that some events reported as SAEs may not require expedited reporting to the regulatory authorities based on the classification of "common serious adverse events". You are required to follow the requirements detailed in [Section 5.5.6] Reporting of Serious Adverse Events (SAE).

For an investigational new drug (IND) safety reporting, single occurrences of the following events may be excluded from expedited reporting to the regulatory agencies (e.g., the FDA). If aggregate analysis of these events indicates that they occur more frequently with study drug, an expedited IND safety report may be submitted to the regulatory agencies.

- Anemia
- Leukopenia
- Lymphopenia
- Neutropenia and febrile neutropenia
- Thrombocytopenia
- Pancytopenia
- Cholestatic liver disease
- Sinusoidal obstruction syndrome (Hepatic veno-occlusive disease)
- Abnormal LFTs
- Relapse of primary malignancy or disease
- The following protocol-defined clinical endpoint events as reported by the Investigator, including those with a fatal outcome:
  - CMV infection and CMV diseases including but may not be limited to those listed in [Appendix 2]
  - GVHD (any grade), acute or chronic, including liver involvement

12.4 Appendix 4: Protocol Defined Definitions of Comorbidities for the HCT-Comorbidity Index

Comorbidity	Definition	Modified HCT-CI Weighted Score
Arrhythmia	Atrial fibrillation or flutter, sick sinus syndrome, or ventricular arrhythmias	1
Cardiac*	Coronary artery disease,§ congestive heart failure, myocardial infarction, or ejection fraction (EF) ≤ 50%	1
Inflammatory Bowel Disease	Crohn’s disease or ulcerative colitis	1
Diabetes	Requiring treatment with insulin or oral hypoglycemic but not diet alone	1
Cerebrovascular disease	Transient ischemic attack or cerebrovascular accident	1
Psychiatric disturbance	Unstable depression or anxiety condition that the Investigator believes will interfere with protocol requirements, including compliance with long-term follow-up.	1
Hepatic, mild	Chronic hepatitis, bilirubin > ULN to 1.5 X ULN, or AST/ALT > ULN to 2.5 X ULN	1
Obesity	Patients with a body mass index > 35 kg/m^2	1
Infection	Requiring continuation of antimicrobial treatment after Day 0	1
Rheumatologic	Systemic lupus erythematosus (SLE), rheumatoid arthritis (RA), polymyositis, mixed connective tissue disease (CTD), or polymyalgia rheumatic	2
Peptic Ulcer	Requiring treatment	2
Moderate/severe renal**	Serum creatinine > 2 mg/dL, on dialysis, or prior renal transplantation	2
Moderate pulmonary*	Diffusion capacity of carbon monoxide (DLco) and/or FEV_1 66%-80% or dyspnea on slight activity	2
Prior solid tumor	Treated at any time point in the patient’s past history, excluding nonmelanoma skin cancer	3
Heart valve disease	Except mitral valve prolapsed	3
Severe pulmonary*	DLco and/or FEV_1 ≤ 65% or dyspnea at rest or requiring oxygen	3
Moderate/severe hepatic	Liver cirrhosis, bilirubin > 1.5 X ULN, or AST/ALT > 2.5 X ULN	3

*Pulmonary function tests (PFTs), multi gated acquisition scans (MUGAs), and/or echocardiograms performed within 6 months (180 days) prior to Screening may be used for scoring pulmonary and cardiac comorbidities.

**When assessing Renal function, to convert creatinine from micromoles per liter (Umole/L) to milligrams per deciliter (mg/dL), divide micromoles per liter by 88.4 (Umole/L ÷ 88.4 = mg/dL).

The definitions for each comorbidity based on definitions published by Sorror in 2013 are provided below:

Arrhythmia (score 1): Any type of arrhythmia that has necessitated the delivery of a specific anti-arrhythmia treatment at any time in the patient's past medical history. A score is assigned even if the patient was in normal sinus rhythm at the time of data acquisition or at the landmark date.
No score is assigned to transient arrhythmias that never required treatment.

Cardiovascular Comorbidity (score 1): Assigned for cardiovascular comorbidity in the presence of 1 or more of the following 3 presentations:

1. Coronary artery disease: This is based on the presence of a documented diagnosis of chronic exertional angina, unstable angina, or myocardial infarction at any point the patient's past medical history. Information on prior placement of a coronary stent or undergoing a coronary artery bypass graft surgery should support coding this comorbidity.
2. Congestive heart failure: At any time in the patient's medical history, symptoms/signs of congestive heart failure (e.g., an exertional or paroxysmal nocturnal dyspnea) that later responded to diuretics, afterload-reducing agents, B-blocker, and/or digitalis.
3. Low Ejection Fraction (EF). Patients with an EF of 50% or lower as determined by an echocardiogram or a multigated acquisition scan.
   This should be from the most recent measurements.

Inflammatory bowel disease (score 1): The presence of documented prior diagnosis (history of an endoscopic examination of the mucosa with or without confirmatory histology and radiologic findings) of Crohn's disease or ulcerative colitis requiring treatment at any time in the patient's past medical history.

Diabetes (score 1): Diagnosis of diabetes or steroid-induced hyperglycemia requiring continuous treatment with insulin or oral hypoglycemic drugs *within 4 weeks prior to screening.*

Cerebrovascular disease (score 1): A prior diagnosis of transient ischemic attack, subarachnoid hemorrhage, or cerebral thrombosis, embolism, or hemorrhage at any time in the past medical history.

Psychiatric disorder (score 1): Unstable depression or anxiety condition that the Investigator believes will interfere with protocol requirements, including compliance with long-term follow-up*

Hepatic comorbidity (score 2 levels of severity): *The safety laboratories obtained at screening should be used to assess this score.*

1. **Mild hepatic comorbidity (score 1):** The presence of 1 or more of the following 3 clinical presentations: (1) elevated total bilirubin to a value higher than the upper limit of normal (ULN) and up to 1.5 times the ULN. (2) elevated values of alanine aminotransferase (ALT) or aspartate aminotransferase (AST) to values higher than the ULN and up to 2.5 times the ULN; or (3) a prior diagnosis of an infection with hepatitis B or C at any time in the patient's past medical history.
2. **Moderate to severe hepatic comorbidity (score 3):** The presence of 1 or more of the following 3 clinical presentations: (1) elevated values of total bilirubin to a level higher than 1.5 times the ULN; (2) elevated values of ALT or AST to levels higher than 2.5 times the ULN or (3) a documented diagnosis of liver cirrhosis at any time in the patient's past medical history.

Obesity (score 1): A body mass index higher than 35.00 kg/m^2

Infection (score 1): The presence of 1 or more of the following 4 clinical presentations at screening: (1) a documented infection (e.g., by culture or biopsy); (2) fever of unknown origin; (3) pulmonary nodules suspicious for fungal pneumonia; (4) a requirement for prophylaxis against tuberculosis.

Table continued on next page

**Rheumatologic comorbidity (score 2):** The presence of a documented prior diagnosis of an autoimmune rheumatologic disease that has required administration of a specific treatment at any time in the patient's past medical history. Diagnosis include systemic lupus erythematosus, rheumatoid arthritis, Sjogren's syndrome, scleroderma, polymyositis, dermatomyositis, mixed connective tissue disease, polymylagia rheumatic, polychondritis, sarcoidosis and vasculitis syndromes. It does not include degenerative joint disease (osteoarthritis) or other musculoskeletal injuries.
**Peptic ulcer (score 2):** The presence of a prior endoscopic or radiologic diagnosis of gastric or duodenal ulcer at any point in the patient's past medical history.
**Renal comorbidity (score 2):** *The creatinine from the safety laboratories obtained at screening should be used to assess this score.*<br>The presence of 1 or more of the following 3 presentations: (1) elevated values of serum creatinine to more than 2 mg/dL or more than 176.8 umol/L; (2) chronic renal disease requiring weekly dialysis within 4 weeks of screening; (3) a documented prior history of renal transplantation at any point in the patient's past medical history.
**Pulmonary comorbidity (score 2 different levels of severity):** The diffusing lung capacity of carbon monoxide (DLco) should be corrected for the concurrent hemoglobin. The most recent set of pulmonary function test (PFTs) are to be used for criteria using DLco and forced expiratory volume (FEV1).<br>1. **Moderate pulmonary comorbidity (score 2):** The presence of 1 or more of the following 3 clinical presentations: (1) a corrected percentage of DLco in the range of 66% to 80%; (2) a percentage of FEV1 in the range of 66% to 80%; (3) shortness of breath on slight activity that is attributed to a pulmonary disease and cannot be corrected by blood transfusion for a noticeable anemia within 2 weeks of screening.<br>2. **Severe pulmonary comorbidity (score 3):** The presence of 1 or more of the following 4 clinical presentations: (1) a corrected percentage of DLco of 65% or less; (2) a percentage of FEV1 of 65% or less; (3) shortness of breath at rest that is attributed to a pulmonary disease and cannot be corrected by blood transfusion for a noticeable anemia within 2 weeks prior to screening; (4) the need for intermittent or continuous oxygen supplementation within 4 weeks of screening.
**Prior malignancy (score 3):** The presence of a prior diagnosis of any malignancy that required receiving a specific treatment at any point in the patient's past medical history except for the malignancy for which the patient is receiving the hematopoietic cell transplant (HCT). Patients with a prior malignancy from the same lineage of cells of the current malignancy should not be assigned a score for this comorbidity (e.g., if a subject had a diagnosis of non-Hodgkin lymphoma that was preceded by Hodgkin lymphoma or if a patient had a diagnosis of an acute myeloid leukemia that was preceded by myelodysplastic syndrome).<br>*Basal cell carcinomas and squamous cell carcinoma of the skin should not be assigned a score for this comorbidity.*
**Heart valve disease (score 3):** *The most recent echocardiogram, if performed, obtained should be used to score this comorbidity.*<br>The presence of 1 or more of the following 3 clinical presentations: (1) at least a moderate or severe degree of valve stenosis or insufficiency, as determined by echocardiogram; (2) prosthetic mitral or aortic valve; (3) symptomatic mitral valve prolapse.

Reference:

Sorror ML, Maris MB, Storb R, Baron F, Sandmaier MB, Maloney DG, et al. Hematopoietic cell transplantation (HCT)-specific comorbidity index: a new tool for risk assessment before allogeneic HCT. Blood.. 2005;106:2912-19.

Sorror, ML. How I assess comorbidities before hematopoietic cell transplantation. Blood. 2013;121;2854-63.

12.5 Appendix 5: Dynamic International Prognostic Scoring System for Survival in Primary Myelofibrosis

	**Value**		
**Prognostic Variable**	**0**	**1**	**2**
Age, y	≤ 65	> 65	
White blood cell count , X10^9/L	≤ 25	> 25	
Hemoglobin, g/dL	≥ 10		< 10
Peripheral blood blast, %	< 1	≥ 1	
Constitutional symptoms, Y/N	N	Y	

The risk category is obtained by adding up the values of each prognostic variable. Risk categories are defined as:

- Low: 0
- Intermediate-1: 1 or 2
- Intermediate-2: 3 or 4
- High: 5 or 6.

Reference:

Passamonti F, Cervantes F, Vannucchi AM, Morra E, Rumi E, Pereira A, et al. A dynamic prognostic model to predict survival in primary myelofibrosis: a study by the IWG-MRT (International Working Group for Myeloproliferative Neoplasms Research and Treatment). Blood. 2010;115:1703-08.

12.6 Appendix 6: Definition of Morphologic Complete Remission

Complete remission will be defined as all of the following according to the revised recommendations of the international working group:

1. A bone marrow aspirate containing spicules with < 5% blasts with a count of at least 200 nucleated cells and no Auer rods seen. If spicules are absent in the aspirate, a bone marrow biopsy should confirm that < 5% blasts are present.
2. No evidence of a persistently abnormal leukemic population by flow cytometry, if performed†.
3. Absolute neutrophil count > 1000/μL and platelet count > 100000/μL.
4. No extramedullary leukemia.
5. No blasts in peripheral blood.

†: If a result of flow cytometry is available, complete remission needs to be confirmed based all criteria including No.2. Otherwise, complete remission will be evaluated per the above criteria except for No.2.

Reference:

Cheson BD, Bennett JM, Kopecky KJ, Buchner T, Willman CL, Estey EH, et al. Revised recommendations of the international working group for diagnosis, standardization of response criteria, treatment outcomes, and reporting standards for therapeutic trials in acute myeloid leukemia. J Clin Oncol. 2003; 21(24): 4642-9.

12.7 Appendix 7: Grading of Reactogenicity

**Local Reaction To Injectable Product**	**Mild (Grade 1)**	**Moderate (Grade 2)**	**Severe (Grade 3)**	**Potentially Life -Threatening (Grade 4)**
**Pain**	Does not interfere with activity	Repeated use of non-narcotic pain reliever > 24 hours or interferes with activity	Any use of narcotic pain reliever or prevents daily activity	Emergency room (ER) visit or hospitalization
**Tenderness**	Mild discomfort to touch	Discomfort with movement	Significant discomfort at rest	ER visit or hospitalization
**Erythema/Redness†**	2.0 – 5 cm	5.1 – 10 cm	> 10 cm	Necrosis or exfoliative dermatitis
**Induration /Swelling‡**	2.0 – 5 cm and does not interfere with activity	5.1 – 10 cm or interferes with activity	> 10 cm or prevents daily activity	Necrosis

† In addition to grading the measured local reaction at the greatest single diameter, the measurement should be recorded as a continuous variable.

‡ Induration/Swelling is to be evaluated and graded using the functional scale as well as the actual measurement.

Reference:

Guidance for Industry titled "Toxicity Grading Scale for Healthy Adult and Adolescent Volunteers Enrolled in Preventive Vaccine Clinical Trials" issued by FDA on September 2007.

12.8 Appendix 8: Severity Grading Table and Recurrence Interval Definitions for Infections

Type of Infection	Grade 1	Grade 2	Grade 3
**Bacterial infections**	Bacterial focus NOS requiring no more than 14 days of therapy for treatment (e.g., urinary tract infection)	Bacteremia (except coagulase-negative staphylococcus) without severe sepsis†	Bacteremia with deep organ involvement (e.g. with new or worsening pulmonary infiltrates; endocarditis)
	Coag Neg Staph (S. epi), Corynebacterium, or Proprioniobacterium bacteremia	Bacterial focus with persistent signs, symptoms or persistent positive cultures requiring greater than 14 days of therapy	Severe sepsis with bacteremia.
	Cellulitis responding to initial therapy within 14 days	Cellulitis requiring a change in therapy d/t progression<br>Localized or diffuse infections requiring incision with or without drain placement	Fasciitis requiring debridement
		Any pneumonia documented or presumed to be bacterial	Pneumonia requiring intubation
			Brain abscess or meningitis without bacteremia
	C. Difficile toxin positive stool with diarrhea < 1L without abdominal pain (child < 20 mL/kg)	C. Difficile toxin positive stool with diarrhea ≥ 1L (child ≥ 20 mL/kg) or with abdominal pain	C. Difficile toxin positive stool with toxic dilatation or renal insufficiency with/without diarrhea
**Fungal infections**	Superficial candida infection (e.g., oral thrush, vaginal candidiasis)	Candida esophagitis (biopsy proven).	Fungemia including Candidemia
		Proven or probable fungal sinusitis confirmed radiologically without orbital, brain or bone involvement.	Proven or probable invasive fungal infections (e.g., Aspergillus, Mucor, Fusarium, Scedosporium)

Table continued on next page

Type of Infection	Grade 1	Grade 2	Grade 3
**Fungal infections continued**			Disseminated infections (defined as multifocal pneumonia, presence of urinary or blood antigen, and/or central nervous system involvement) with Histoplasmosis, Blastomycosis, Coccidiomycosis, or Cryptococcus<br><br>*Pneumocystis jiroveci* pneumonia (regardless of partial pressure of oxygen level)
**Viral infections**	Mucous HSV infection<br><br>Dermatomal Zoster<br><br>Asymptomatic CMV viremia untreated or a CMV viremia with viral load decline by at least 2/3 of the baseline value after 2 weeks of therapy<br><br>EBV reactivation not treated with rituximab<br><br>Adenoviral conjunctivitis asymptomatic viruria, asymptomatic stool shedding and viremia not requiring treatment<br><br>Asymptomatic (HHV-6) viremia untreated or an HHV-6 viremia with a viral load decline by at least 0.5 log after 2 weeks of therapy<br><br>BK viremia or viruria with cystitis not requiring intervention	VZV infection with 3 or more dermatomes<br><br>Clinically active CMV infection (e.g. symptoms, cytopenias) or CMV Viremia not decreasing by at least 2/3 of the baseline value after 2 weeks of therapy<br><br>EBV reactivation requiring institution of therapy with rituximab<br><br>Adenoviral upper respiratory infection, viremia, or symptomatic viruria requiring treatment<br><br>Clinically active HHV-6 infection (e.g., symptoms, cytopenias) or HHV-6 viremia without viral load decline 0.5 log after 2 weeks of therapy<br><br>BK viremia or viruia with clinical consequence requiring prolonged therapy and/or surgical intervention	Severe VZV infection (coagulopathy or organ involvement)<br><br>CMV end-organ involvement (pneumonitis, enteritis, retinitis)<br><br>EBV PTLD<br><br>Adenovirus with end-organ involvement (except conjunctivitis and upper respiratory tract)

Table continued on next page

Type of Infection	Grade 1	Grade 2	Grade 3
**Viral infections continued**	 <br>Viremia (virus not otherwise specified) not requiring therapy	Enterocolitis with enteric viruses<br><br>Symptomatic upper tract respiratory virus<br><br>Any viremia (virus not otherwise specified) requiring therapy	 <br>Lower tract respiratory viruses<br><br>Any viral encephalitis or meningitis
**Parasitic infections**			Central nervous system or other organ toxoplasmosis<br><br>Strongyloides hyperinfection
**Nonmicro-biologically defined infections**	Uncomplicated fever with negative cultures responding within 14 days<br><br>Clinically documented infection not requiring inpatient management	 <br>Pneumonia or bronchopneumonia not requiring mechanical ventilation<br><br>Typhlitis	 <br>Any acute pneumonia requiring mechanical ventilation<br><br>Severe sepsis† without an identified organism

CMV: cytomegalovirus; EBV: Epstein Barr virus; HHV-6: human herpes virus 6; HSV: herpes simplex virus; NOS: not otherwise specified; PTLD: post-transplant lymphoproliferative disorder; VZV: varicella zoster virus

*†**Severe Sepsis** is defined as the following:*

Adults: Hypotension

A systolic blood pressure of < 90 mmHg or a reduction of > 40 mmHg from baseline in the absence of other causes for hypotension

Multiple Organ Dysfunction Syndrome

Two or more of the following: Renal failure requiring dialysis, respiratory failure requiring bipap or intubation, heart failure requiring pressors, liver failure.

Disseminated Infections:

Two or more non-contiguous sites with the SAME organism

A disseminated infection can occur at any level of severity, but most will be grade 2 or 3.

Recurrence Intervals to Determine Whether an Infection is the Same or New:

CMV, HSV, EBV, HHV6: 2 months (< 60 days)

VZV, HZV: 2 weeks (< 14 days)

Bacterial, non-Clostridium difficile: 1 week (< 7 days)

Bacterial, Clostridium difficile: 1 month (< 30 days)

Yeast: 2 weeks (< 14 days)

Molds: 3 months (< 90 days)

Helicobacter: 1 year (< 365 days)

Adenovirus, Enterovirus, Influenza, RSV, Parainfluenza, Rhinovirus: 2 weeks (< 14 days)

Polyomavirus (BK virus): 2 months (< 60 days)

For infections coded as “Disseminated,” any previous infection with the same organism but different site within the recurrence interval for that organism will be counted as part of the disseminated infection.

Reference:

Blood and Marrow Transplant Clinical Trials Network, Technical MOP, Version 3.0, 19 Mar 2013. Accessed from http://www.bmtctn.net. Accessed on 31 August 2015.

12.9 Appendix 9: Staging and Grading of Acute Graft versus Host Disease

Staging of aGVHD

**Skin†**	0 = No rash<br>1 = Maculopapular rash < 25% of body surface<br>2 = Maculopapular rash 25%-50% of body surface<br>3 = Generalized erythroderma (maculopapular rash > 50%)<br>4 = Generalized erythroderma with bullous formation and desquamation
**Lower Intestinal Tract‡**	0 = Diarrhea < 500 mL/day or 280 mL/m2<br>1 = Diarrhea > 500 but ≤ 1000 mL/day or 280-555 mL/m2<br>2 = Diarrhea > 1000 but ≤ 1500 mL/day or 556-833 mL/m2<br>3 = Diarrhea > 1500 mL/day or > 833mL/m2<br>4 = Severe abdominal pain with or without ileus or stool with frank blood or melena
**Upper Intestinal Tract§**	0 = No protracted nausea and vomiting<br>1 = Persistent nausea, vomiting, or anorexia
**Liver¶**	0 = Bilirubin < 2.0 mg/dL<br>1 = Bilirubin 2.0 – 3.0 mg/dL<br>2 = Bilirubin 3.1 – 6.0 mg/dL<br>3 = Bilirubin 6.1 – 15.0 mg/dL<br>4 = Bilirubin > 15.0 mg/dL
**Biopsy Result**	PS = Positive<br>NG = Negative<br>EQ = Equivocal<br>ND = Not done

†Use 'Rule of Nines' or burn chart to determine extent of rash.

‡Downgrade 1 stage if an additional cause of diarrhea has been documented.

§Persistent nausea with histologic evidence of GVHD in the stomach or duodenum.

¶Range given as total bilirubin. Downgrade 1 stage if an additional cause of elevated bilirubin has been documented.

Overall Grade of aGVHD†

**Grade 0:**<br>● No skin rash and<br>● No protracted nausea and vomiting and<br>● No diarrhea or diarrhea < 500 mL/day and<br>● Bilirubin < 2.0 mg/dL	**Grade III:**<br>● Skin – no rash to rash > 50% of body surface with<br>● Diarrhea >1500 mL/day or severe abdominal pain or<br>● Bilirubin 3.1 – 15.0 mg/dL
**Grade I:**<br>● Skin Rash < 50% of body surface and<br>● No diarrhea or diarrhea < 500 mL/day and<br>● Bilirubin < 2.0 mg/dL	**Grade IV‡:**<br>● Skin – generalized erythroderma or<br>● Bilirubin > 15.0 mg/dL
**Grade II:**<br>● Skin rash > 50% of body surface or<br>● Diarrhea > 500 mL/day or<br>● Bilirubin 2.0 – 3.0 mg/dL or<br>● Persistent nausea/vomiting	

†Criteria for grading given as minimum degree of organ involvement required to confer that grade.

‡Grade IV may also include lesser organ involvement but with extreme decrease in performance status.

References:

BMT CTN 0201 Data listing for data review committee (DRC). Updated 12 Mar 2010.

Przepiorka D, Weisdorf D, Martin P, Klingemann H-G, Beatty P, Hows J, and Thomas ED. Meeting report: Consensus conference on acute GVHD grading. BMT 1995; 15:825-8.

12.10 Appendix 10: Rule of Nines Burn Chart

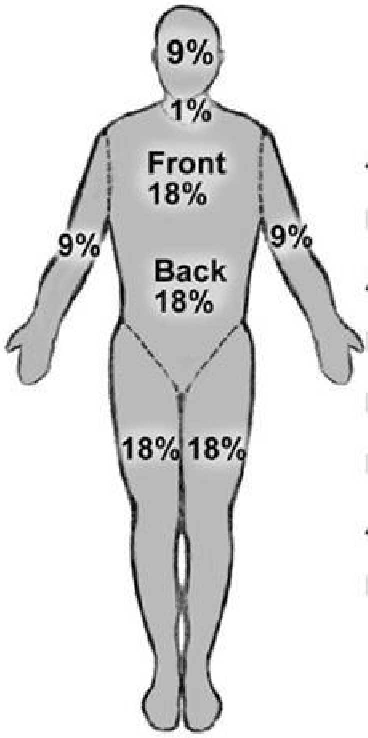

Adult body Part	% of total BSA
Arm	9%
Head	9%
Neck	1%
Leg	18%
Anterior trunk	18%
Posterior trunk	18%

12.11 Appendix 11: Criteria for the Diagnosis of Chronic Graft Versus Host Disease

The diagnosis of cGVHD requires the following 3 criteria:

1. Distinction from acute GVHD,
2. Presence of at least 1 diagnostic clinical sign of chronic GVHD or presence of at least 1 distinctive manifestation confirmed by pertinent biopsy or other relevant tests,
3. Exclusion of other possible diagnosis.

Organ or Site	Diagnostic (Sufficient to Establish the Diagnosis of cGVHD)	Distinctive (Seen in cGVHD, but Insufficient Alone to Establish a Diagnosis of cGVHD)	Other Features	Common (seen with Both Acute and Chronic GVHD)
**Skin**	Poikiloderma<br>Lichen planus-like features<br>Sclerotic features<br>Morphea-like features<br>Lichen sclerosus-like features	Depigmentation	Sweat impairment<br>Ichthyosis<br>Keratosis pilaris<br>Hypopigmentation<br>Hyperpigmentation	Erythema<br>Maculopapular rash<br>Pruritus
**Nails**		Dystrophy<br>Longitudinal ridging, splitting, or brittle features<br>Onycholysis<br>Pterygium unguis<br>Nail loss (usually symmetric; affects most nails)†		
**Scalp and body hair**		New onset of scarring or nonscarring<br>Scalp alopecia (after recovery from chemoradiotherapy)<br><br>Scaling, papulosquamous lesions	Thinning scalp<br>Hair, typically patchy, coarse, or dull (not explained by endocrine or other causes)<br>Premature gray hair	
**Mouth**	Lichen-type features<br>Hyperkeratotic plaques<br>Restriction of mouth opening from sclerosis	Xerostomia<br>Mucocele<br>Mucosal atrophy<br><br>Pseudomembranes†<br>Ulcers†		Gingivitis<br>Mucositis<br>Erythema<br><br>Pain
**Eyes**		New onset dry, gritty, or painful eyes‡<br>Cicatricial conjunctivitis<br>Keratoconjunctivitis sicca‡<br>Confluent areas of punctate<br><br>Keratopathy	Photophobia<br>Periorbital<br>Hyperpigmentation<br>Blepharitis (erythema of the eyelids with edema)	
**Genitalia**	Lichen planus-like features<br>Vaginal scarring or stenosis	Erosions†<br>Fissures†<br>Ulcers†		

Table continued on next page

Organ or Site	Diagnostic (Sufficient to Establish the Diagnosis of cGVHD)	Distinctive (Seen in cGVHD, but Insufficient Alone to Establish a Diagnosis of cGVHD)	Other Features	Common (seen with Both Acute and Chronic GVHD)
**Gastrointestinal Tract**	Esophageal web<br>Strictures or stenosis in the upper mid third of the esophagus†		Exocrine<br>Pancreatic insufficiency	Anorexia<br>Nausea<br>Vomiting<br>Diarrhea<br>Weight loss<br>Failure to thrive (infants and children)
**Liver**				Total bilirubin,<br>Alkaline phosphatase > 2 x ULN<br>Alanine aminotransferase or aspartate aminotransferase >2 x ULN
**Lung**	Bronchiolitis obliterans diagnosed with lung biopsy	Bronchiolitis obliterans diagnosed with<br>Pulmonary function test and radiology‡		Bronchiolitis obliterans organizing pneumonia
**Muscles, fascia, joint**	Fasciitis<br>Joint stiffness or contractures secondary to sclerosis	Myositis or polymyositis‡	Edema<br>Muscle cramps<br>Arthralgia or arthritis	
**Hematopoietic and immune systems**			Thrombocytopenia<br>Eosinophilia<br>Lymphopenia<br>Hypo- or Hyper γ-globulinemia<br>Autoantibodies (autoimmune hemolytic anemia and idiopathic thrombocytopenic purpura)	

Table continued on next page

Organ or Site	Diagnostic (Sufficient to Establish the Diagnosis of cGVHD)	Distinctive (Seen in cGVHD, but Insufficient Alone to Establish a Diagnosis of cGVHD)	Other Features	Common (seen with Both Acute and Chronic GVHD)
**Other**			Pericardial or Pleural effusions<br>Ascites<br>Peripheral neuropathy<br>Nephrotic syndrome<br>Myasthenia gravis<br>Cardiac conduction abnormality or cardiomyopathy	

aGVHD: acute graft-versus-host disease; cGVHD: chronic graft-versus-host disease; GVHD: graft-versus-host disease; ULN: upper limit of normal

†In all cases, infection, drug effects, malignancy, or other causes must be excluded.

‡Diagnosis of chronic GVHD requires biopsy or radiology confirmation (or Schirmer test for eyes).

Note: Other features can be acknowledged as part of the chronic GVHD symptomatology if the diagnosis is confirmed.

Reference:

Filipovich AH, Weisdorf D, Pavletic S, Socie G, Wingard JR, Lee SJ, et al. National institutes of health consensus development project on criteria for clinical trials in chronic graft-versus-host disease: 1. diagnosis and staging working group report. Biology of Blood and Marrow Transplantation. 2005; 11:945-55.

12.12 Appendix 12: Grading of Chronic Graft versus Host Disease

Maximum grade of chronic GVHD

Limited - localized skin involvement and/or hepatic dysfunction due to cGVHD.

Extensive - 1 or more of the following:

- Generalized skin involvement; or,
- Liver histology showing chronic aggressive hepatitis, bridging necrosis or cirrhosis; or,
- Involvement of the eye: Schirmer's test with < 5 mm wetting; or,
- Involvement of minor salivary glands or oral mucosa demonstrated on labial biopsy; or,
- Involvement of any other target organ.

References:

CIBMTR Form 2200 revision 2, June 2009.

Shulman HIM, Sullivan KM, Weiden PL, McDonald GB, Striker GE, Sale GE, et al. Chronic-graft-versus-host syndrome in man: A long-term clinicopathologic study of 20 Seattle patients. AJM 1980; 69:204-17.

12.13 Appendix 13: Organ Scoring and Global Scoring of Chronic Graft versus Host Disease

Organ Scoring cGVHD

	SCORE 0	SCORE 1	SCORE 2	SCORE 3
**PERFORMANCE SCORE:** ☐ **KPS**	☐ Asymptomatic and fully active (KPS 100%)	☐ Symptomatic, fully ambulatory, restricted only in physically strenuous activity (KPS 80%-90%)	☐ Symptomatic, ambulatory, capable of self-care, >50% of waking hours out of bed (KPS 60%-70%)	☐ Symptomatic, limited self-care, >50% of waking hours in bed (KPS <60%)
**SKIN**<br>*Clinical features*<br>☐ Maculopapular rash<br>☐ Lichen planus-like features<br>☐ Papulosquamous lesions or ichthyosis<br>☐ Hyperpigmentation<br>☐ Hypopigmentation<br>☐ Keratosis pilaris<br>☐ Erythema<br>☐ Erythroderma<br>☐ Poikiloderma<br>☐ Sclerotic features<br>☐ Pruritus<br>☐ Hair involvement<br>☐ Nail involvement<br>**%BSA Involved** ☐	☐ No symptoms	☐ 18% BSA with disease signs but **NO** sclerotic features	☐ 19%-50% BSA **OR** involvement with superficial sclerotic features "not hidebound" (able to pinch)	☐ 50% BSA **OR** deep sclerotic features "hidebound" (unable to pinch) **OR** impaired mobility, ulceration or severe pruritus
**MOUTH**	☐ No symptoms	☐ Mild symptoms with disease signs but not limiting oral intake significantly	☐ Moderate symptoms with disease signs **with** partial limitation of oral intake	☐ Severe symptoms with disease signs on examination **with** major limitation of oral intake
**EYES**<br>Mean tear test (mm):<br>☐ >10<br>☐ 6-10<br>☐ ≤ 5<br>☐ Not done	☐ No symptoms	☐ Mild dry eye symptoms not affecting ADL (requiring eye drops ≤ 3 x per day) **OR** asymptomatic signs of keratoconjunctivitis sicca	☐ Moderate dry eye symptoms partially affecting ADL (requiring drops > 3 x per day or punctual plugs), **WITHOUT** vision impairment	☐ Severe dry eye symptoms significantly affecting ADL (special eyewear to relieve pain) **OR** unable to work because of ocular symptoms **OR** loss of visions caused by keratoconjunctivitis sicca
Table continued on next page				

	SCORE 0	SCORE 1	SCORE 2	SCORE 3
**GI Tract**	☐ No symptoms	☐ Symptoms such as dysphagia, anorexia, nausea, vomiting, abdominal pain or diarrhea without significant weight loss (<5%)	☐ Symptoms associated with mild to moderate weight loss (5%-15%)	☐ Symptoms associated with significant weight loss >15%, requires nutritional supplement for most calorie needs **OR** esophageal dilation
**LIVER**	☐ Normal LFT	☐ Elevated Bilirubin, AP*, AST or ALT < 2 x ULN	☐ Bilirubin > 3 mg/dL or Bilirubin, enzymes 2 x-5 x ULN	☐ Bilirubin or enzymes > 5 x ULN
**LUNGS**[†]<br>**FEV1** ☐<br>**DLCO** ☐	☐ No symptoms<br>☐ FEV1 > 80% **OR** LFS = 2	☐ Mild symptoms (shortness of breath after climbing 1 flight of steps)<br>☐ FEV1 60%-79% **OR** LFS = 3-5	☐ Moderate symptoms (shortness of breath after walking on flat ground)<br>☐ FEV1 40%-59% **OR** LFS = 6-9	☐ Severe symptoms (shortness of breath at rest; requiring 0_2)<br>☐ FEV1 ≤ 39% **OR** LFS = 10-12
**JOINTS AND FASCIA**	☐ No symptoms	☐ Mild tightness of arms or legs, normal or mild decreased ROM **AND** not affecting ADL	☐ Tightness of arms or legs OR joint contractures, erythema thought due to fasciitis, moderate decrease ROM **AND** mild to moderate limitation of ADL	☐ Contractures **WITH** significant decrease of ROM ***AND*** significant limitation of ADL (unable to tie shoes, button shirts, dress self etc.)
**GENITAL TRACT**	☐ No symptoms	☐ Symptomatic with mild signs on exam **AND** no effect on coitus and minimal discomfort with gynecologic exam	☐ Symptomatic with moderate signs on exam **AND** with mild dyspaareunia or discomfort with gynecologic exam	☐ Symptomatic **WITH** advanced signs (stricture, labial agglutination or severe ulceration) **AND** severe pain with coitus or inability to insert vaginal speculum

Other indicators, clinical manifestations or complications related to chronic GVHD (check all that apply and assign a score to its severity (0-3) base on its functional impact where applicable (none - 0, mild - 1, moderate -2, severe - 3)

Esophageal stricture or web______ Pericardial Effusion ______ Pleural Effusion(s) ______

Ascites (serositis) ______ Nephrotic syndrome ______ Peripheral Neuropathy ______

Myasthenia Gravis ______ Cardiomyopathy ______ Eosinophilia > 500/ µL ______

Polymyositis ______ Cardiac conduction defects ______ Coronary artery involvement ______

Platelets < 100,000/µL ______ Progressive onset ______

OTHERS: Specify: ____

ADL: activities of daily living; BSA: bovine serum albumin; cGVHD: chronic graft-versus-host disease; DLCO: diffusing lung capacity of carbon monoxide; FEV1: forced expiratory volume ; GVHD: graft-versus-host disease; KPS: Karnofsky Performance Scale ; LFS: lung function score; PFT: pulmonary function testing; ROM: range of motion

Footnotes continued on next page

Organ scoring of chronic GVHD. *AP may be elevated in growing children, and not reflective of liver dysfunction. †Pulmonary scoring is to be performed using both the symptom and PFT scale whenever possible, if performed based on symptoms. When discrepancy exists between pulmonary symptom or PFT scores the higher value should be used for final scoring. Scoring using the LFS is preferred, but if DLCO is not available, grading using FEV1 should be used. The LFS is a global assessment of lung function after the diagnosis of bronchiolitis obliterans has already been established. The percent predicted FEV1 and DLCO (adjusted for hematocrit but not alveolar volume) should be converted to a numeric score as follows: >80% = 1; 70%-79% = 2; 60%-69% = 3; 50%-59% = 4; 40%-49% = 5; < 40% = 6. The LFS = FEV1 score + DLCO score, with a possible range of 2-12.

Global Scoring of cGVHD

- Mild: 1 or 2 organs or sites (except lung), with no clinically significant functional impairment (maximum of score 1 in all affected organs or sites).
- Moderate:
    - At least 1 organ or site with clinically significant but no major disability (maximum score of 2 in any affected organ or site) or;
    - 3 or more organs or sites with no clinically significant functional impairment (maximum score of 1 in all affected organs or sites) or;
    - A lung score of 1
- Severe:
    - Major disability caused by chronic GVHD (score of 3 in any organ or site);
    - A lung score of 2 or greater

Modified from Reference:

Filipovich AH, Weisdorf D, Pavletic S, Socie G, Wingard JR, Lee SJ, et al. National institutes of health consensus development project on criteria for clinical trials in chronic graft-versus-host disease: 1. diagnosis and staging working group report. Biology of Blood and Marrow Transplantation 2005;11:945-55.

12.14 Appendix 14: EQ-5D Questionnaire

Note that the below is an example; patients will be provided with forms to be completed.

Under each heading, please tick the ONE box that best describes your health TODAY

MOBILITY

I have no problems in walking about ☐
I have slight problems in walking about ☐
I have moderate problems in walking about ☐
I have severe problems in walking about ☐
I am unable to walk about ☐

SELF-CARE

I have no problems washing or dressing myself ☐
I have slight problems washing or dressing myself ☐
I have moderate problems washing or dressing myself ☐
I have severe problems washing or dressing myself ☐
I am unable to wash or dress myself ☐

USUAL ACTIVITIES *(e.g. work, study, housework, family or leisure activities)*

I have no problems doing my usual activities ☐
I have slight problems doing my usual activities ☐
I have moderate problems doing my usual activities ☐
I have severe problems doing my usual activities ☐
I am unable to do my usual activities ☐

PAIN / DISCOMFORT

I have no pain or discomfort ☐
I have slight pain or discomfort ☐
I have moderate pain or discomfort ☐
I have severe pain or discomfort ☐
I have extreme pain or discomfort ☐

ANXIETY / DEPRESSION

I am not anxious or depressed ☐
I am slightly anxious or depressed ☐
I am moderately anxious or depressed ☐
I am severely anxious or depressed ☐
I am extremely anxious or depressed ☐

SAMPLE

UK (English) v.2 © 2009 EuroQol Group. EQ-5D™ is a trade mark of the EuroQol Group

Appendix 14: EQ-5D Questionnaire continued

- We would like to know how good or bad your health is TODAY.
- This scale is numbered from 0 to 100.
- 100 means the best health you can imagine.
  0 means the worst health you can imagine.
- Mark an X on the scale to indicate how your health is TODAY.
- Now, please write the number you marked on the scale in the box below.

YOUR HEALTH TODAY = ☐

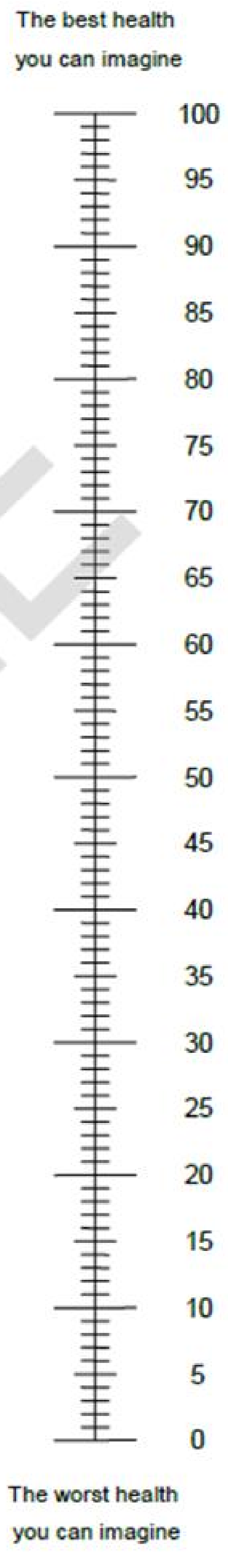

SAMPLE

UK (English) v.2 © 2009 EuroQol Group. EQ-5D™ is a trade mark of the EuroQol Group

12.15 Appendix 15: FACT-BMT (Version 4)

Note that the below is an example; patients will be provided with forms to be completed.

Below is a list of statements that other people with your illness have said are important. **Please circle or mark 1 number per line to indicate your response as it applies to the past 7 days.**

	PHYSICAL WELL-BEING	Not at all	A little bit	Some-what	Quite a bit	Very much
GP1	I have a lack of energy	0	1	2	3	4
GP2	I have nausea	0	1	2	3	4
GP3	Because of my physical condition, I have trouble meeting the needs of my family	0	1	2	3	4
GP4	I have pain	0	1	2	3	4
GP5	I am bothered by side effects of treatment	0	1	2	3	4
GP6	I feel ill	0	1	2	3	4
GP7	I am forced to spend time in bed	0	1	2	3	4

	SOCIAL/FAMILY WELL-BEING	Not at all	A little bit	Some-what	Quite a bit	Very much
GS1	I feel close to my friends	0	1	2	3	4
GS2	I get emotional support from my family	0	1	2	3	4
GS3	I get support from my friends	0	1	2	3	4
GS4	My family has accepted my illness	0	1	2	3	4
GS5	I am satisfied with family communication about my illness	0	1	2	3	4
GS6	I feel close to my partner (or the person who is my main support)	0	1	2	3	4
Q1	*Regardless of your current level of sexual activity, please answer the following question. If you prefer not to answer it, please mark this box and go to the next section.*					
GS7	I am satisfied with my sex life	0	1	2	3	4

Appendix 15: FACT-BMT (Version 4) continued

Please circle or mark 1 number per line to indicate your response as it applies to the <u>past 7 days</u>.

	EMOTIONAL WELL-BEING	Not at all	A little bit	Some-what	Quite a bit	Very much
GE1	I feel sad………………………………….…	0	1	2	3	4
GE2	I am satisfied with how I am coping with my illness……………………………………..…	0	1	2	3	4
GE3	I am losing hope in the fight against my illness……………………………………..…	0	1	2	3	4
GE4	I feel nervous…………………………….…..	0	1	2	3	4
GE5	I worry about dying……………………….....	0	1	2	3	4
GE6	I worry that my condition will get worse……….	0	1	2	3	4

	FUNCTIONAL WELL-BEING	Not at all	A little bit	Some-what	Quite a bit	Very much
GF1	I am able to work (include work at home)…..…	0	1	2	3	4
GF2	My work (include work at home) is fulfilling….	0	1	2	3	4
GF3	I am able to enjoy life……………………….....	0	1	2	3	4
GF4	I have accepted my illness……………….…...	0	1	2	3	4
GF5	I am sleeping well………………………………	0	1	2	3	4
GF6	I am enjoying the things I usually do for fun…..	0	1	2	3	4
GF7	I am content with the quality of my life right now………………………………………………..	0	1	2	3	4

Appendix 15: FACT-BMT (Version 4) continued

Please circle or mark 1 number per line to indicate your response as it applies to the <u>past 7 days</u>.

	ADDITIONAL CONCERNS	Not at all	A little bit	Some-what	Quite a bit	Very much
BMT1	I am concerned about keeping my job (include work at home)……………………………………	0	1	2	3	4
BMT2	I feel distant from other people…………………..	0	1	2	3	4
BMT3	I worry that the transplant will not work………..	0	1	2	3	4
BMT4	The effects of treatment are worse than I had imagined………………………………………………	0	1	2	3	4
C6	I have a good appetite……………………………	0	1	2	3	4
C7	I like the appearance of my body………………..	0	1	2	3	4
BMT5	I am able to get around by myself……………….	0	1	2	3	4
BMT6	I get tired easily………………………………….	0	1	2	3	4
BL4	I am interested in sex……………………………	0	1	2	3	4
BMT7	I have concerns about my ability to have children…………………………………………..	0	1	2	3	4
BMT8	I have confidence in my nurse(s)………………..	0	1	2	3	4
BMT9	I regret having the bone marrow transplant……..	0	1	2	3	4
BMT10	I can remember things…………………………..	0	1	2	3	4
Br1	I am able to concentrate…………………………	0	1	2	3	4
BMT11	I have frequent colds/infections…………………	0	1	2	3	4
BMT12	My eyesight is blurry…………………………….	0	1	2	3	4
BMT13	I am bothered by a change in the way food tastes……………………………………………….	0	1	2	3	4
BMT14	I have tremors……………………………………	0	1	2	3	4
B1	I have been short of breath………………………	0	1	2	3	4
BMT15	I am bothered by skin problems (e.g., rash, itching)……………………………………………..	0	1	2	3	4
BMT16	I have trouble with my bowels…………………..	0	1	2	3	4
BMT17	My illness is a personal hardship for my close family members………………………………….	0	1	2	3	4
BMT18	The cost of my treatment is a burden on me or my family………………………………………..	0	1	2	3	4

12.16 Appendix 16: Work Productivity and Activity Impairment Questionnaire: General Health Version 2.0

The following questions ask about the effect of your health problems on your ability to work and perform regular activities. By health problems we mean any physical or emotional problem or symptom. *Please fill in the blanks or circle a number, as indicated.*

1. Are you currently employed (working for pay)? ____ NO____ YES
   If NO, check "NO" and skip to question 6.

The next questions are about the **past 7 days**, not including today.

2. During the past 7 days, how many hours did you miss from work because of your health problems? Include hours you missed on sick days, times you went in late, left early, etc., because of your health problems. Do not include time you missed to participate in this study.
   _____HOURS

3. During the past 7 days, how many hours did you miss from work because of any other reason, such as vacation, holidays, time off to participate in this study?
   _____HOURS

4. During the past 7 days, how many hours did you actually work?
   _____HOURS (*If "0", skip to question 6.*)

5. During the past 7 days, how much did your health problems affect your productivity while you were working?

   Think about days you were limited in the amount or kind of work you could do, days you accomplished less than you would like, or days you could not do your work as carefully as usual. If health problems affected your work only a little, choose a low number. Choose a high number if health problems affected your work a great deal.

Consider only how much health problems affected productivity while you were working.

Health problems had no effect on my work	0	1	2	3	4	5	6	7	8	9	10	Health problems completely prevented me from working

CIRCLE A NUMBER

Appendix 16: Work Productivity and Activity Impairment Questionnaire: General Health V2.0 (WPAI:GH) continued

6. During the past 7 days, how much did your health problems affect your ability to do your regular daily activities, other than work at a job?

   By regular activities, we mean the usual activities you do, such as work around the house, shopping, childcare, exercising, studying, etc. Think about times you were limited in the amount or kind of activities you could do and times you accomplished less than you would like. If health problems affected your activities only a little, choose a low number. Choose a high number if health problems affected your activities a great deal.

Consider only how much health problems affected your ability to do your regular daily activities, other than work at a job.

Health problems had no effect on my daily activities	0	1	2	3	4	5	6	7	8	9	10	Health problems completely prevented me from doing my daily activities

CIRCLE A NUMBER

12.17 Appendix 17: Definitions of Conditioning Regimens

Myeloablative conditioning regimen: regimens with total body irradiation single dose of ≥ 500 cGY, fractionated dose of ≥ 800 cGY, busulfan doses of > 9mg/kg, or melphalan doses of > 150 mg/m^2 given as single agents or in combination with other drugs.

Non-myeloablative (reduced-intensity) conditioning regimen: regimens with lower doses of total body irradiation, fractionated radiation therapy, busulfan, and melphalan than those used to define a myeloablative conditioning regimen (above).

Reference

Pasquini MC, Wang Z. Current use and outcome of hematopoietic stem cell transplantation: CIBMTR Summary Slides, 2009.

12.18 Appendix 18: Liver Safety Monitoring and Assessment

Drug-induced liver injury (DILI) has been the most frequent single cause of safety-related marketing withdrawals for the past 50 years. Robust monitoring and expedited regulatory reporting of DILI during clinical trials are therefore required by the FDA (FDA Guidance, 2009). However, monitoring and reporting of elevated liver function tests (LFTs) will be confounded in this trial because elevated LFTs are common in patients within 1 year after a hematopoietic cell transplant. Therefore, while LFTs will be monitored throughout this trial, extra pharmacovigilance will be performed only on those subjects that have marked elevations. Confirmed abnormalities will be characterized as marked where upper limit of normal (ULN):

	ALT or AST		Total Bilirubin
**Marked**	> 20 x ULN	Or	≥ 10 x ULN

ALT: alanine aminotransferase; AST: aspartate aminotransferase; ULN: upper limit of normal

If laboratory testing for a subject enrolled in study and with normal serum aminotransferases (AT) and TBL has an increase of serum AT > 5x ULN or total bilirubin (TBL) > 2x ULN by either local or central laboratory, the medical monitor is to be notified within 24 hours. If the enrolled subject had abnormal AT or TBL at time of enrollment and there is an increase in the AT to > 5 x baseline or the TBL to > 2x baseline, the medical monitor is to be notified within 24 hours. Alerts will be generated by the central laboratory regarding abnormalities to inform the Investigator, study monitor and study team. Notification to the medical monitor only needs to be done at the onset of LFT elevations. However, if the subject has normal LFTs for > 2 weeks between the LFT elevations described above, the medical monitor is to be notified for each event.

If laboratory testing for a subject enrolled in the study reveals an increase of serum AT to > 20x ULN, or TBL ≥ 10x ULN, at least all 4 of the usual serum hepatic measures (alanine aminotransferase [ALT], aspartate aminotransferase [AST], alkaline phosphatase [ALP] and TBL) is to be repeated by the central laboratory and the medical monitor is to be notified within 24 hours of the elevated LFTs. Testing should be repeated within 48 to 72 hours of notification of the test results. Alerts will be generated by the central laboratory regarding marked liver abnormality to inform the Investigator, study monitor and study team. Subjects should be asked if they have any symptoms suggestive of hepatobiliary dysfunction.

Follow-up Procedures

Confirmed marked abnormalities in hepatic functions should be thoroughly characterized by obtaining appropriate expert consultations, detailed pertinent history, PE and laboratory tests. The site is to complete the Liver Abnormality Case Report Form (LA-CRF) or an appropriate document. Information collected will include the following:

- Diagnosis of condition causing the elevated LFTs
- Central and local laboratory measurement of elevated LFTs (including AST, ALT, TBL, direct bilirubin, and ALP)
- Concurrent medical conditions

- Concurrent medications
- Prior conditioning medications with doses
- Alcohol and illegal substance use
- Hepatitis A, B, C and CMV and EBV testing to detect active replication of virus
- Liver imaging and biopsy results, if performed

Confirmed marked abnormal LFTs should be repeated 2-3 times weekly and then weekly or less if abnormalities stabilize or the study drug has been discontinued and the subject is asymptomatic.

Study Discontinuation

In the absence of an explanation for increased LFTs, such as viral hepatitis, pre-existing or acute liver disease or exposure to other agents associated with liver injury, the subject may be discontinued from treatment. The Investigator may determine that it is not in the subject's best interest to continue study participation.

In addition, if close monitoring for a subject with marked hepatic laboratory tests is not possible, drug must be discontinued.

DMC Monitoring

In addition to the above, the DMC will assess unblinded events between the ASP0113 and placebo groups to determine if there appears to be a signal that the frequency or severity of liver events is greater in ASP0113 than placebo or/and than that reported in the literature.

Reference

Guidance for Industry titled "Drug-Induced Liver Injury: Premarketing Clinical Evaluation" issued by FDA on July 2009.

12.19 Appendix 19: Pharmacogenomics (PGx) Sub-Study

INTRODUCTION/BACKGROUND

PGx research aims to provide information regarding how naturally occurring changes in a subject's gene and/or expression based on genetic variation may impact what treatment options are best suited for the subject. Through investigation of PGx by technologies such as genotyping, gene sequencing, statistical genetics and Genome-Wide Association Studies (GWAS), the relationship between gene profiles and a drug's kinetics, efficacy or toxicity may be better understood. PGx research may identify which genes are involved in determining the way a subject may or may not respond to a drug.

STUDY OBJECTIVES

The PGx research that may be conducted in the future with acquired saliva samples is exploratory. The objective of this research will be to analyze or determine genes of relevance to clinical response, and toxicity/safety issues.

By analyzing genetic variations, it may be possible to predict an individual subject's response to ASP0113 in terms of efficacy and/or toxicity.

STUDY POPULATION

Subjects who have consented to participate in this study may also participate in the optional pharmacogenomics sub-study. As part of this sub-study, subjects must provide separate written informed consent prior to providing any saliva samples that will be used at a later time for the pharmacogenomic analysis.

STUDY DESIGN

Site Sample Collection and Storage

Subjects who consent to participate in the PGx sub-study will provide 1 saliva sample at baseline (V2) prior to dosing. Each sample will be identified with a unique identifier. Samples will be collected and shipped to the Sponsor designated central laboratory for prolonged storage. Detailed collection, storage, and shipment procedures will be included in the Laboratory Manual for the study.

Central Laboratory and Research Laboratory Processing and Storage / Sample Coding

Once received at the banking CRO, the samples will be assigned a unique sample code (second code) and stored frozen. A table linking the subject number (first code) with the newly-assigned sample code (second code) will be kept by the banking CRO. PGx analysis will be conducted using the second code only.

PHARMACOGENOMIC ANALYSIS

Details on the potential PGx analysis cannot be established yet. Astellas may initiate the PGx analysis in case evidence suggests that genetic variants may be influencing the drug's kinetics,

efficacy and/or safety. Prior to initiating any analysis on the banked samples, the Astellas ethical committee (AREC) must approve the analysis plan.

DISPOSAL OF PHARMACOGENOMIC SAMPLES/DATA

All PGx samples collected will be stored for a period of up to 15 years following study database hardlock. If there is no requirement for analysis, the saliva sample will be destroyed after the planned storage period. The subject has the right to withdraw consent at any time. When a subject's withdraw notification is received, the PGx sample will be destroyed. The results of any PGx analysis conducted on a sample prior to its withdrawal will be retained at Astellas indefinitely.

INFORMATION DISCLOSURE TO THE SUBJECTS

Retrospective PGx analysis may be conducted following the conclusion of the clinical study, if applicable. The results of the genetic analysis will not be provided to any investigators or subjects, nor can the results be requested at a later date. Any information that is obtained from the PGx analysis will be the property of Astellas.

12.20 Appendix 20: Long-term Follow-up Questionnaire

Subjects will be contacted by telephone annually after the primary study period (Day 365) through 5.5 years post-transplant for long-term safety related to the DNA vaccine. Items to be assessed by telephone interview and available patient records include mortality, development of any new/recurrent cancer, development of infection requiring hospitalization, and local immune-mediated reactions.

The following information will be collected at each of the contact time points:

1. Subject mortality, including primary cause of death and date of death (if applicable).
2. Development of any new/recurrent cancer (name).
3. Development of infection requiring hospitalization or resulting in death (name of infecting agent, or medications or laboratory data leading to diagnosis).
    - The subject will be asked whether he/she has been diagnosed with any new medical conditions requiring hospitalization to solicit this information.
4. Local immune-mediated reactions.
    - The subject will be asked if he/she has had any redness or swelling at the sites of the study drug injections.

12.21 Appendix 21: Health Economic Assessment (Inpatient and Outpatient Utilization)

Are there any Inpatient Care visits to report? □Yes □No

If Yes, please record the number of days the patient was admitted to:

Admission Unit/Floor

ICU Days_____________
# of Days

Step-down Unit Days______________
# of Days

Hospital General Medical/Surgical Ward Days_____________
# of Days

Was there an Emergency Room visit greater than 24 hours (without admittance)? □Yes □No

If yes, please indicate the number of Emergency Room days____________
# of days

Primary Reason (Diagnosis) for Admission:___________________

If a US site, please insert the ICD-9 code for the diagnosis.

If more than 1 Inpatient Care visit to report, please record diagnosis/ICD-9 code for each admission, and the number of days as noted above.

Are there any Non- Protocol- related Physician visits to report? □ Yes □No

If Yes, please indicate the number of visits:

Physician Visits_____________
# of visits

Are there any Emergency Room visits less than or equal to 24 hours to report? □ Yes □No

If Yes, please indicate the number of visits:

ER visits (≤ 24 hours)__________
# of visits

12.22 Appendix 22: Karnofsky Performance Scale

**100%**	Normal. No complaints. No evidence of disease.
**90%**	Able to carry on normal activity. Minor signs of disease.
**80%**	Normal activity with effort. Some signs and symptoms of disease.
**70%**	Cares for self. Unable to carry on normal activity or do active work.
**60%**	Requires occasional assistance, but is able to care for most personal needs.
**50%**	Requires considerable assistance and frequent medical care.
**40%**	Disabled. Requires special care and assistance.
**30%**	Severely disabled. Hospitalization is indicated, although death not imminent.
**20%**	Hospitalization necessary, very sick, active support treatment is necessary.
**10%**	Moribund. Fatal processes progressing rapidly.
**0%**	Dead.

Reference:

Karnofsky DA, Burchenal JH. The clinical evaluation of chemotherapeutic agents in cancer, in Macleod cm (ed): Evaluation Of Chemotherapeutic Agents. New York, Columbia University Press, 1949 p 199-205.

13 ATTACHMENT 1: COUNTRY-SPECIFIC NONSUBSTANTIAL AMENDMENT 2 [JP]

I. The purpose of this amendment is:

**Non-Substantial Changes**
**1. Update Contract Research Organization (CRO) Contact Information**
DESCRIPTION OF CHANGE:
Update the CRO name, telephone and fax number for serious adverse event (SAE) reporting in Japan.
RATIONALE:
Due to a change in CRO for managing the study sites in Japan, the 24-hour contact information for SAEs for sites in Japan requires an update.
**2. Update Key Sponsor Contact Information**
DESCRIPTION OF CHANGE:
Update the name and contact information for the Astellas Pharma Inc. medical monitor/medical expert and study manager assigned to this study.
RATIONALE:
Due to resource changes at Astellas Pharma Inc., the medical monitor/medical expert and study manger assigned to the Japanese sites for this study requires updates.

II. Amendment Summary of Changes:

**II. CONTACT DETAILS OF KEY SPONSOR PERSONNEL**<br>*24h-Contact for Serious Adverse Events (SAEs)*
WAS:
For sites in Japan only:<br>Please fax the JUTOKUNA YUUGAIJISHOU HOUKOKUSHO to the delegated CRO:<br>PPD [redacted]
IS AMENDED TO:
For sites in Japan only:<br>Please fax the JUTOKUNA YUUGAIJISHOU HOUKOKUSHO to the delegated CRO:<br>PPD [redacted]

PPD

II. CONTACT DETAILS OF KEY SPONSOR PERSONNEL<br>*Medical Monitor/Medical Expert:*
WAS:
PPD MD<br>PPD<br>Immunology, Transplant, Infectious Disease, CNS and Pain,<br>Astellas Pharma Global Development, Inc.<br>PPD
IS AMENDED TO:
PPD MD**, PhD**<br>PPD<br>~~Immunology, Transplant, Infectious Disease, CNS and Pain,~~<br>PPD **Medical & Development**<br>Astellas Pharma Global Development, Inc.<br>PPD

II. CONTACT DETAILS OF KEY SPONSOR PERSONNEL<br>*Clinical Research Contacts:*
WAS:
For sites in Japan only:<br>PPD<br>Japan/Asia Clinical Development 1,<br>Astellas Pharma Inc.<br>PPD
IS AMENDED TO:
For sites in Japan only:<br>PPD<br>Japan/Asia Clinical Development 1,<br>Astellas Pharma Inc.

PPD

**5 TREATMENTS AND EVALUATION**<br>*5.5.6 Reporting of SAEs*
WAS:
For contact details for each country/region, see Section II Contact Details of Key Sponsor's Personnel. Please fax the SAE Worksheet (Specific to sites in Japan: JUTOKUNA YUUGAIJISHOU HOUKOKUSHO) to:<br>PPD
IS AMENDED TO:
For contact details for each country/region, see Section II Contact Details of Key Sponsor's Personnel. Please fax the SAE Worksheet (Specific to sites in Japan: JUTOKUNA YUUGAIJISHOU HOUKOKUSHO) to:<br>PPD

III. Non-Substantial Amendment Rationale:

Rationale for Non-Substantial Designation
All revisions made to the protocol are administrative in nature and do not impact the safety or scientific value of the clinical study.

14 SPONSOR'S SIGNATURES
