# Supplementary figures and images for: A randomised, placebo-controlled phase 3 study to evaluate the efficacy and safety of ASP0113, a DNA-based CMV vaccine, in seropositive allogeneic haematopoietic cell transplant recipients
Source: eClinicalMedicine. 2021 Mar 19;33:100787. doi: 10.1016/j.eclinm.2021.100787 (PMC8020145; doi:10.1016/j.eclinm.2021.100787)

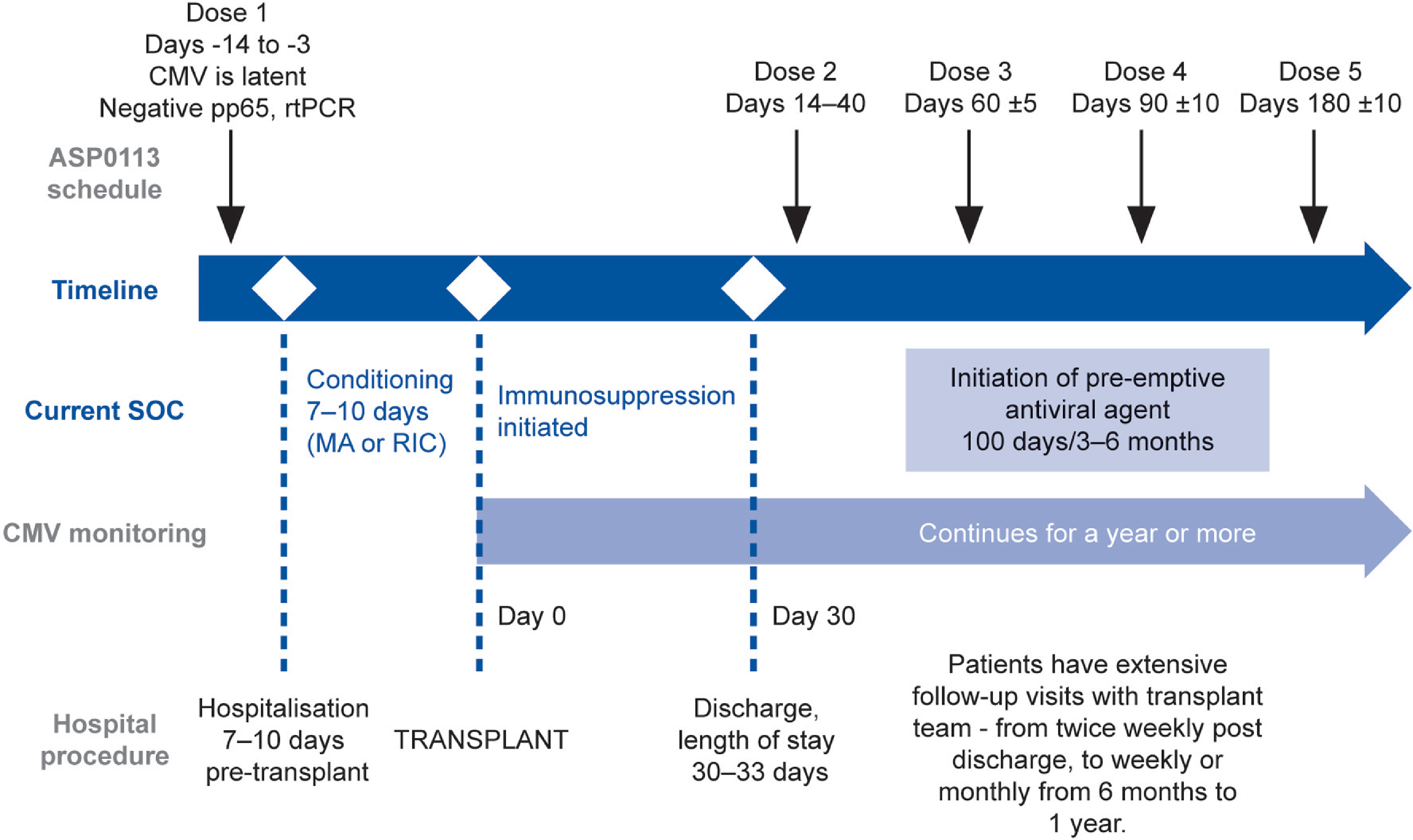

Supplement: Supplementary file 2 [file mmc2.jpg]
